# Revealing the Reasons for Degeneration of Resonance-Assisted Hydrogen Bond on the Aromatic Platform: Calculations of Ortho-, Meta-, Para-Disubstituted Benzenes, and (*Z*)-(*E*)-Olefins

**DOI:** 10.3390/molecules28020536

**Published:** 2023-01-05

**Authors:** Andrei V. Afonin, Danuta Rusinska-Roszak

**Affiliations:** 1 A. E. Favorsky Irkutsk Institute of Chemistry, Siberian Division, Russian Academy of Sciences, Irkutsk, 664033 Russia; 2 Institute of Chemical Technology and Engineering, Poznan University of Technology, 60-965 Poznan, Poland

**Keywords:** resonance-assisted hydrogen bond, intramolecular hydrogen bond energy, decomposition energy into components, conductivity of push-pull effect, molecular tailoring approach, isomers of disubstituted benzenes and olefins

## Abstract

The energies of the O−H∙∙∙O=C intramolecular hydrogen bonds were compared quantitatively for the series of ortho-disubstituted benzenes and *Z*-isomers of olefins via a molecular tailoring approach. It was established that the hydrogen bond energy in the former series is significantly less than that in the latter one. The reason for lowering the hydrogen bond energy in the ortho-disubstituted benzenes compared to the *Z*-isomers of olefins is the decrease in the π-contribution to the total energy of the complex interaction, in which the hydrogen bond *per se* is enhanced by the resonance effect. By the example of the para- and meta-disubstituted benzenes, as well as *E*-isomers of olefins, it was explicitly shown that the aromatic ring is a much poorer conductor of the resonance effect compared to the double bond. The hydrogen bond in the ortho-disubstituted benzenes has a lower energy than a typical resonance-assisted hydrogen bond because the aromatic moiety cannot properly assist the hydrogen bond with a resonance effect. Thus, a hydrogen bond on an aromatic platform should fall into a special category, namely an aromaticity-assisted hydrogen bond, which is closer by nature to a simple hydrogen bond rather than to a resonance-assisted one.

## 1. Introduction

Resonance-assisted hydrogen bonding (RAHB) is one of the well-established concepts of modern theoretical and structural chemistry, developed in 1989 due to the works of Gilli et al. [1,2,3,4]. Although the RAHB concept has been disputed and revisited many times [5,6,7,8,9,10,11,12], it is still a valid and commonly accepted theory [13,14,15,16,17,18,19,20,21,22,23,24,25,26,27]. Nevertheless, an in-depth study of the RAHB nature remains an urgent challenge, since the presence of such a hydrogen bond affects the structure, physicochemical properties, and reactivity of molecules. Compounds with the RAHB find application in targeted organic synthesis, crystal engineering, medicinal chemistry, and materials science, as well as in supramolecular chemistry, molecular recognition, solvatochromism, etc. [28]. Moreover, the RAHB concept was recognized as a milestone in the development of hydrogen bond design principles in the recent review by Wu et al., dedicated to the 100th anniversary of the Hydrogen Bond [29].

RAHBs belong to the category of especially strong H-bonds and are identified by their specific spectral and structural features. The IR and ^1^H NMR spectra of the O−H∙∙∙O=C RAHB-containing substances reveal an extraordinary decrease in the ν_O–H_ vibration frequency to 2550–2675 cm^−1^ and a large downfield shift of the bridging hydrogen resonance to 15–17 ppm, respectively [2,30]. In addition, the HRD data show a shortening in the O∙∙∙O distance to 2.4–2.6 Å [1,2,3]. According to the RAHB concept, the strength of the hydrogen bond increases due to the resonance assistance during the transfer of the electron density from the H-donor atom to the H-acceptor one [1,2,3,4].

The relationship between aromaticity and H-bond strength in the RAHB-containing structures has been extensively investigated. In this regard, one should distinguish the inter- and intramolecular RAHBs, the study of which require different approaches. With regard to intermolecular RAHBs, the important concept of aromaticity-modulated hydrogen bonding (AMHB) was introduced by Wu and Jackson et al. [31,32,33]. In keeping with the AMHB concept, in the H-bonding compounds the aromaticity gain strengths the H-bond, while the aromaticity loss weakens the H-bond [31,32,33]. The AMHB concept can be applied not only to conventional H-bonds, but also to nonclassical ones [34]. More importantly, the AMHB concept is supported not only by theoretical calculations, but also by experimental data [33].

The energy of the intermolecular RAHB [E(inter_RAHB)] between a donor molecule D and an acceptor molecule A can be quantified directly from Equation (1a):

*E*(inter_RAHB) = *E*(D•A) – [*E*(D) + *E*(A)] (1a)

where *E*(D•A) is the total energy of the complex D•A, *E*(D), and *E*(A) are the energy of donor molecule D and acceptor molecule A, respectively.

When using Equation (1a), the basis set superposition error (BSSE correction) should be taken into account. However, this approach is not suitable for the quantification of the intramolecular RAHB energy, since this would require the division of a whole molecule into parts. The energy of an intramolecular hydrogen bond (IMHB) is an inseparable contribution to the total energy of a molecule [35,36,37]. Essentially, the IMHB energy is a virtual quantity that can neither be measured experimentally nor calculated directly [35,36,37]. Nevertheless, two methods have been developed to indirectly estimate the IMHB energy. The first one can be designated as the function-based approach (FBA), which uses the functional dependence (2a) of the IMHB energy [*E*(IMHB)] on the H-bond descriptor (denotes as P parameter) [37,38]

*E*(IMHB) = *f*(P) (2a)

The *ρ*_BCP_ (electron density at the H-bond critical points), *V*_BCP_ (potential energy density at the H-bond critical point), *r*_O···H_ (H-bond distance), ∆ν (shift of the X–H bond vibration due to X–H∙∙∙Y H-bond) and ∆δ (change in the chemical shift of a bridging hydrogen atom in the ^1^H NMR spectra due to H-bond) parameters are conventionally used as the H-bond descriptors [39,40,41,42,43,44,45,46,47].

The second method for quantifying the IMHB energy is the molecular tailoring approach (MTA), which is based on the fragmentation of molecules (*vide infra*). Currently, this method is effectively employed to evaluate the IMHB energy in medium and large molecules [37,38,48,49,50,51,52,53,54,55,56,57,58,59,60].

RAHB is a complex interaction, consisting of σ- and π-components, which are responsible for the H-bond *per se* and resonance assistance, respectively. Application of the FBA method allows the energy of the RAHB σ-component to be estimated since this method is based on the properties of the σ-skeleton of molecules [37,38,60]. The MTA method estimates the total energy of intramolecular RAHB [38,60]. The difference between the RAHB energies, quantified by the FBA and MTA methods, gives an estimation of the π-contribution to the total RAHB energy [38,60]. In this way, the separation of the total RAHB energy into σ- and π-components was successfully carried out for classical RAHB structures such as β-diketones [38].

At the same time, the RAHB interaction is assumed to be homogeneous in its essence, i.e., independent of the nature of the unsaturated fragment connecting the H-bond donor and acceptor atoms (whether this fragment is a double bond or an aromatic ring). In the papers dealing with the study of IMHB on an aromatic platform, such IMHB is considered from the same positions as classical RAHBs in β-diketones [7,16,61,62,63,64,65,66]. However, the MTA assessment of the total RAHB energy in compounds with aromatic moiety evidence that it is significantly lower than in those with a double bond [58,59]. The relationship between various structural parameters, as well as bond indices and the intramolecular RAHB energy in compounds on an aromatic platform, was studied by the MTA method [59]. The lengths of covalent bonds within the chelate ring and the qHOMA (quasi-Harmonic Oscillator Model of Aromaticity) index [67] were used as structural parameters, while the Wiberg’s bond index [68] and the index obtained from the DDEC (Density Derived Electrostatic and Chemical) approach [69] were employed as bond indices [59]. It was concluded [59] that the IMHB in compounds on an aromatic platform should be distinguished from classical RAHBs and referred to as aromaticity-assisted hydrogen bonds (arom-AHB). However, in this work, the separation of the total arom-AHB energy into σ- and π-components was not made. Hence, the reasons for the decrease in the total arom-AHB energy with respect to the classical RAHB remained unclear. In order to understand why arom-AHB and classical intramolecular RAHB differ significantly in energy, in the present paper, the total arom-AHB energy was separated into σ- and π-components using the FBA and MTA methods. In addition, the σ- and π-components of arom-AHB were compared with those of RAHB.

**Figure 1:**
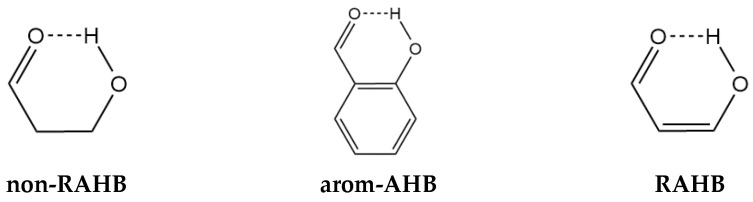
Scheme for comparing the arom-AHB interaction with the non-RAHB and RAHB ones.

To quantify how arom-AHB differs from the conventional RAHB, the following methodology was applied. On the one hand, a very large series of the hydroxycarbonyl aliphatic compounds as non–conjugated structures were studied in detail and their O−H···O=C IMHB energies were estimated using the MTA method [56]. For simplicity, these non-conjugated structures were referred to as the non-RAHB structures throughout the article. On the other hand, an equally extensive series of the β-diketones and related compounds (RAHB structure) were thoroughly studied and their O−H···O=C IMHB energies were also evaluated by the same MTA method [57]. Finally, the O−H···O=C IMHB energies were assessed by the MTA method for representative series of the ortho-hydroxybenzaldehydes, phenones, and quinones, in which the arom-AHB is realized [58]. Thus, a vast number of data on the quantitative IMHB energies was obtained for three different series of compounds representing the non-RAHB-, RAHB-, and arom-AHB structures. The former two series are two diametrically opposite poles of compounds carrying IMHB of different natures, which can serve as a benchmark for comparison and establishing the character of the H-bonds (whether the H-bond is RAHB or not) in other compounds. The latter series of compounds, placed between the above two poles, allows one to accurately determine the degree of similarity (or dissimilarity) of the arom-AHB interaction with the RAHB one (see Figure 1)

The aim of the work is not only to reveal the difference between the arom-AHB and conventional RAHB interactions, but also to explain the reasons for this difference, if it is significant. The MTA method permits both to divide the total energy of the RAHB interaction into σ- and π-contributions and to quantify the pure resonance π-component using isomeric compounds without an H-bond in form of push-pull effect (PPE) energy [70,71]. The compounds with the arom-AHB studied in this article are mainly ortho-disubstituted benzenes, while the compounds with the RAHB are mainly the *Z*-isomers of olefins. To visually trace the differences between the π-components for the arom-AHB and RAHB, we examined the pure resonance π-contribution in the form of PPE in the isomeric para- and meta-disubstituted benzenes, as well as in the *E*-isomers of olefins.

## 2. Results and Discussion

### 2.1. Specifications of the Studied Compounds

In total, 576 different compounds were studied in this work. Some compounds with the O−H···O=C IMHB were already investigated individually, but they were not considered in aggregate, and general patterns of the changes in the IMHB strength depending on the molecular structure were not established. Other compounds exhibiting PPE were studied in this paper for the first time. Taking into account the wide variety of compounds under investigation, we have carried out their detailed specification.

### 2.2. Previously Studied Compounds with the O−H···O=C Intramolecular Hydrogen Bonding

**Scheme 1:**
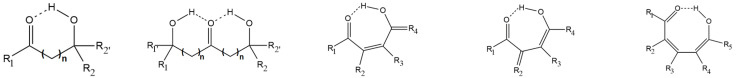
The structure of the typical compounds from the non-RAHB cluster.

**Scheme 2:**
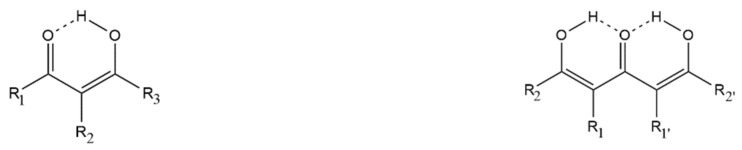
The structure of the typical compounds from the RAHB cluster.

**Scheme 3:**
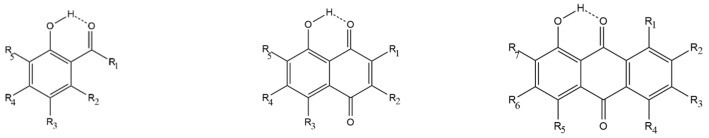
The structure of the typical compounds from the arom-AHB cluster.

In total, 153 Aliphatic hydroxy carbonyl compounds with the O−H···O=C IMHB (non-RAHB structures), 232 β-diketones as well as related compounds with the O−H···O=C IMHB (RAHB structures), and 137 ortho-hydroxybenzaldehydes, phenones, and quinones with the O−H···O=C IMHB (arom-AHB structures), studied in [56,57,58], were not considered in this paper as individual substances. Hence, they were not numbered and were integrated into the non-RAHB, RAHB, and arom-AHB clusters, respectively. Typical compounds from the non-RAHB, RAHB, and arom-AHB clusters are presented in Scheme 1, Scheme 2 and Scheme 3, respectively.

Nevertheless, some clarification regarding compounds from non-RAHB, RAHB, and arom-AHB clusters needs to be made. As noted in [56,57], the compounds with a bifurcated H-bond (second compounds from left to right in Scheme 1 and Scheme 2), exhibit an anticooperative effect. However, the magnitude of the anticooperative effect is insignificant, so they can be considered in the common range of non-RAHB or RAHB structures [56,57]. For the compounds with a bifurcated H-bond, the fragmentation scheme includes six fragments to account for both H-bonds (see Scheme S1, Supplementary Materials, p. S2). It was recognized that the RAHB is implemented only if the 4n + 2 Hückel aromaticity rule is fulfilled for π-conjugated chain [60]. In the rightmost compound in Scheme 1, the eight electrons take part in π-conjugation, so this compound is of the non-RAHB type. The dependences of the IMHB energy quantified by the MTA on the ν_O-H_ frequency and the chemical shift of the hydrogen-bonded proton were previously considered for non-RAHB, RAHB, and arom-RAHB structures [56,57,58]. In addition, the IMHB energies estimated by the MTA method were compared with the IMHB energies estimated by the difference in the energies of conformers with and without an H-bond for the RAHB and arom-AHB structures [57,58].

The compounds belonging to the non-RAHB, RAHB, and arom-AHB clusters are fully decoded in Tables S1–S7, S8–S10, and S11–S15, respectively (see Supplementary Materials, pp. S3–S20). The −*E*_HB_(MTA) values and characteristics of the O−H···O=C IMHB for the compounds from the non-RAHB, RAHB, and arom-AHB clusters are collected in Tables S16–S18, respectively, (Supplementary Materials, pp. S21–S31).

### 2.3. Newly Studied Compounds Exhibiting Push-Pull Effect

Studied compounds **1**–**54** exhibiting the push-pull effect are the R_1_–*Don*-π-*Acc*–R_2_ systems where the *Don* unit is the pyrrole ring, the *Acc* unit is the carbonyl group and the π-linker is the ethenyl or phenyl moiety. The R_1_ and R_2_ substituents at the ends of the *Don*-π-*Acc* system tune the PPE strength. Depending on the nature of the π-linker, compounds **1**–**54** are divided into three subseries. Compounds **1–18** where the π-linker is a double bond form subseries **I**. Compounds **19–36** and **37–54**, where the π-linker is the para- and meta-disubstituted phenyl ring, constitute subseries **II** and **III**, respectively. In turn, each subseries has parts **a** and **b**, depending on which substituent R_1_ or R_2_ varies or fixes. Compounds **1–18**, **19–36,** and **37–54** from subseries **Ia,b**, **IIa,b,** and **IIIa,b** are presented in Table 1 together with the −*E*_π_(PPE) values. The bond length, vibration frequency, HOMO and LUMO energy, and HOMO–LUMO energy gap for calculated compounds **1–54** are collected in Tables S19–S23 (Supplementary Materials, pp. S31–S33). The atom coordinates of compounds **1–54** are given in Supplementary Materials (pp. S77–S104).

### 2.4. Dependences of Hydrogen Bond Energy on Magnitudes of Potential Energy Density at Hydrogen Bond Critical Point for the Non-RAHB, RAHB, and Arom-AHB Clusters

**Figure 2:**
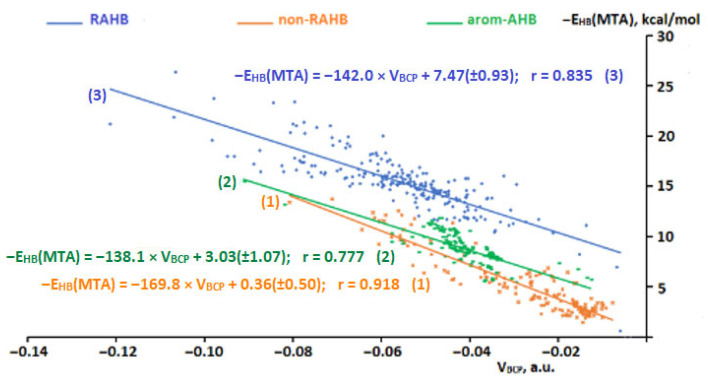
Dependence of the −*E*_HB_(MTA) energy for the non-RAHB, RAHB, and arom-AHB clusters on the *V*_BCP_ values.

The value of the potential energy density at the (3,−3) critical point of an H-bond (*V*_BCP_) is the most important descriptor of an H-bond, since it is widely used to estimate the energy of an H-bond due to the Espinosa-Molins-Lecomte equation and its modified versions [40,45,46,47]. The −*E*_HB_(MTA) values taken from Tables S1–S15 (Supplementary Materials, pp. S3–S20) correlate with the *V*_BCP_ values for compounds from the non-RAHB, RAHB, and arom-AHB clusters (see Equations (1)–(3) on Figure 2). The value of intersection point *B* in linear dependence (1) of −*E*_HB_(MTA) on *V*_BCP_ for compounds from the non-RAHB cluster is equal to 0.36(±0.50) where 0.50 is a 99% confidence interval for the *B* constant. Thus, the statistical error in determining the *B* constant is greater than the value of the *B* constant itself. This means that the −*E*_HB_(MTA) value for compounds from the non-RAHB cluster approaches zero as the *V*_BCP_ value tends to zero, taking into account the statistical uncertainty in determining the *B* constant. However, the magnitude of intersection point *B* in linear dependence (2) of −*E*_HB_(MTA) on *V*_BCP_ for compounds from the arom-AHB cluster rises to 3.03(±1.07). This reveals the presence of an additional contribution of 3(±1) kcal/mol to the −*E*_HB_(MTA) value for compounds from the latter cluster compared to the former one. At last, the magnitude of intersection point *B* in linear dependence (3) of the −*E*_HB_(MTA) values on the *V*_BCP_ ones for compounds from the RAHB cluster grew further to 7.47(±0.93), showing the presence of an additional contribution of 7.5(±1) kcal/mol to the –*E*_HB_(MTA) value for compounds from the RAHB cluster.

In order to interpret these findings, it is necessary to recall that the non-RAHB interaction is a simple H-bond consisting of only σ-component (H-bond *per se*). The RAHB interaction is complex interaction comprising σ- (H-bond *per se*) and π-components (resonance effect). Thus, the appearance of an additional contribution of 7.5(±1) kcal/mol to the −*E*_HB_(MTA) value for compounds from the RAHB cluster can be considered as the presence of a π-contribution to the total RAHB energy. At the same time, this π-contribution reduces to 3(±1) kcal/mol on going to compounds from the arom-AHB cluster. Therefore, the π-contribution to the arom-AHB should be significantly lower compared with the RAHB. This assumption is more fully substantiated in the next section using a wider set of H-bond descriptors, the values of which are divided into subranges.

### 2.5. Comparison of the Hydrogen Bond Energy for Compounds of the Non-RAHB, RAHB, and Arom-AHB Clusters within the Same Range of Hydrogen Bond Descriptors

**Figure 3:**
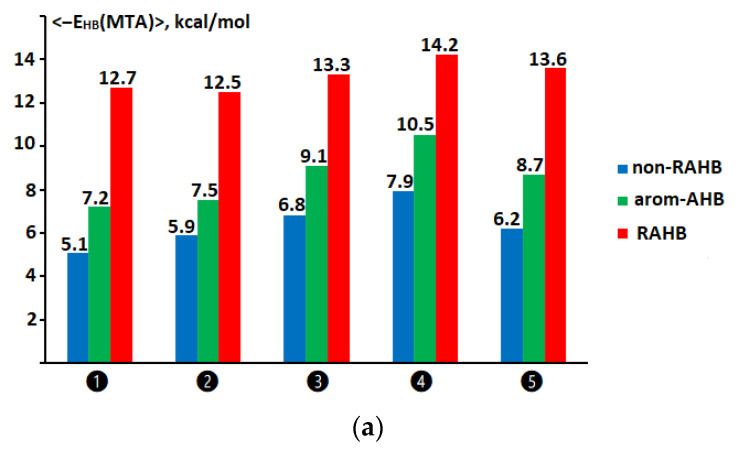

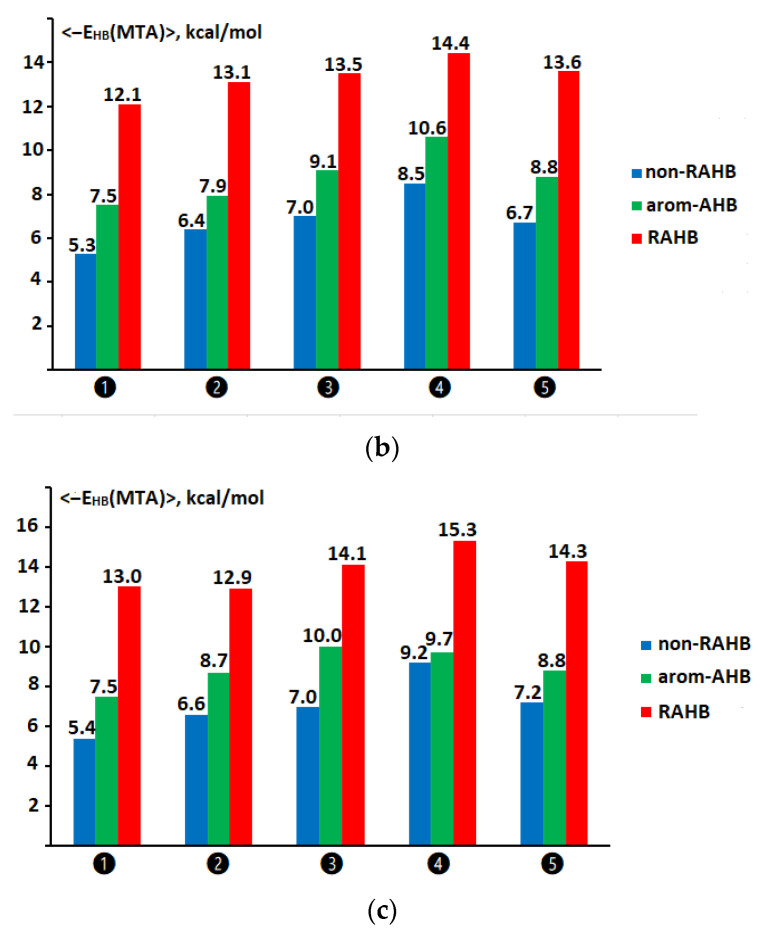
The average <–*E*_HB_(MTA)> IMHB energy for compounds of the non-RAHB (blue), arom-AHB (green), and RAHB (red) clusters estimated via MTA method: range *ρ*_BCP_ and *V*_BCP_—**❶** 0.030–0.035 a.u., **❷** 0.035–0.040 a.u., **❸** 0.040–0.046 a.u., **❹** 0.046–0.051 a.u., **❺** 0.030–0.051 a.u. (total range), (**a**) and (**b**), respectively; range *r*_O∙∙∙H_—**❶** 1.75–1.80 Å, **❷** 1.70–1.75 Å, **❸** 1.65–1.70 Å., **❹** 1.60–1.65 Å, **❺** 1.60–1.80 Å (total range), (**c**).

To quantify differences in the O−H···O=C IMHB energies for compounds of the non-RAHB, RAHB, and arom-AHB cluster, the −*E*_HB_(MTA) values for each of the clusters were compared with the values of the *ρ*_BCP_, *V*_BCP_, and *r*_O···H_ parameters as the most widely used H-bond descriptors [40,42,43,44,45,46,47]. The total intersecting ranges of change in the *ρ*_BCP_, *V*_BCP_, and *r*_O···H_ parameters in the compounds of clusters under investigation are 0.030–0.051 a.u. for both the former and 1.60–1.80 Å for the latter parameter (see Tables S16–S18, Supplementary Materials, pp. S21–S31). In order to more accurately compare the −*E*_HB_(MTA) values with the *ρ*_BCP_, *V*_BCP_, and *r*_O···H_ parameters, these parameters were divided into four subranges (0.030–0.035, 0.035–0.040, 0.040–0.046, and 0.046–0.051 a.u. for both the former and 1.60–1.65, 1.65–1.70, 1.70–1.75, and 1.75–1.80 Å for the latter parameter). The comparison was carried out in such a way that the –*E*_HB_(MTA) energy values would average for all subranges and the total *ρ*_BCP_, *V*_BCP_, and *r*_O···H_ ranges. The results of the comparison are given in Tables S22–S66 (Supplementary Materials, pp. S34–S73) and presented in Figure 3a–c. It can be seen from Figure 3a–c that the average <−*E*_HB_(MTA)> values within the denoted subranges and the total *ρ*_BCP_, *V*_BCP_, and *r*_O···H_ range for compounds of the arom-AHB cluster are closer to those for compounds of the non-RAHB cluster, rather than of the RAHB one.

The difference between the <–*E*_HB_(MTA)> values for compounds of the arom-AHB and non-RAHB clusters is 0.5–3.0 kcal/mol, while the <–*E*_HB_(MTA)> values for compounds of the arom-AHB clusters are less compared with those for compounds of the RAHB clusters by 3.7–5.6 kcal/mol. At the same time, the <–*E*_HB_(MTA)> values for compounds of the RAHB cluster are much higher (by 5.9–7.6 kcal/mol) than those for compounds of the non-RAHB cluster (Figure 3a–c). The <–*E*_HB_(MTA)> values for compounds of the RAHB cluster are significantly larger than that for compounds of the non-RAHB cluster, since the O–H∙∙∙O=C RAHB interaction in the former includes the σ- and π-contributions, while the O–H∙∙∙O=C IMHB in the latter has only the σ-contribution [37,38,60]. The closer similarity of the <–*E*_HB_(MTA)> values for compounds of the arom-AHB cluster with those for compounds of the non-RAHB cluster rather than with the RAHB cluster suggests that the resonance contribution in the arom-AHB interaction is noticeably smaller compared with the RAHB interaction.

### 2.6. Comparison of Hydrogen Bond Energy Values for Compounds of the Non-RAHB, RAHB, and Arom-AHB Clusters Assessed via Molecular Tailoring and Function-Based Approaches

The estimation and comparison of the π-contribution to the O–H∙∙∙O=C arom-AHB and RAHB in compounds of the arom-AHB and RAHB clusters can be implemented in an explicit form using the FBA method which relies on the values of the *ρ*_BCP_, *V*_BCP_, and *r*_O···H_ parameters as the H-bond descriptors and evaluate a pure σ-component of the total RAHB energy (see Introduction). The use of the FBA method for estimating the σ-contribution to the total energy of the RAHB interaction has the following grounds. The critical point for the O–H···O=C H-bond of (3,–3) character is located on the pathway between the oxygen of the C=O group and hydrogen atoms, where the π-cloud of the molecule has a node. For this reason, the *ρ*_BCP_ electron density and the *V*_BCP_ potential energy density, calculated at the (3,–3) H-bond critical point, are determined by the properties of the σ-orbitals. To demonstrate the relationship between the *ρ*_BCP_ and *V*_BCP_ values and the σ-properties of molecules, we performed the Natural Bond Order (NBO) analysis [72] of a sample of 21 compounds in total from the non-RAHB, RAHB, and arom-AHB clusters. The NBO analysis data for compounds **1**, **8a**, **11**, **33a**, **43**, **62**, **88b,** and **94** from the non-RAHB cluster, compounds **6**, **17**, **36**, **42**, **50**, **78,** and **107** from the RAHB cluster, and compounds **13**, **14**, **50**, **62**, **70,** and **104** from the arom-AHB cluster are presented in Table S67 (Supplementary Materials, p. S74).

In the framework of the NBO analysis, the O–H···O=C IMHB in the above compounds appears as the hyper conjugative interactions between two σ-type lone pairs (LP-1 and LP-2) localized on the oxygen atom of C=O group and the antibonding orbital of the O–H bond (the (LP-1(O)_σ_ → σ*(O–H) and (LP-2(O)_σ_ → σ*(O–H) interactions) [42,73,74]. The energies of these LP-1(O)_σ_ → σ*(O–H) and LP-2(O)_σ_ → σ*(O–H) interactions are indicated in Table S67. It is found that the *ρ*_BCP_ and *V*_BCP_ values are directly proportional to the strength of the LP-1(O)_σ_ → σ*(O–H) and (LP-2(O)_σ_ → σ*(O–H) interactions for the sample of compounds from the non-RAHB, RAHB, and arom-AHB clusters [see Equations (3a), (4a), (5a) and (6a)]:

*ρ*_BCP_ = 0.00092 × *E*[(LP-1(O)_σ_ → σ*(O–H)] + 0.0207, r = 0.977, n = 21 (3a)

|*V*_BCP_| = 0.00121 × *E*[(LP-1(O)_σ_ → σ*(O–H)] + 0.0150, r = 0.991, n = 21 (4a)

*ρ*_BCP_ = 0.00867 × *E*[(LP-2(O)_σ_ → σ*(O–H)] + 0.0135, r = 0.942, n = 21 (5a)

|*V*_BCP_| = 0.01102 × *E*[(LP-2(O)_σ_ → σ*(O–H)]+ 0.0065, r = 0.929, n = 21 (6a)

where *E*[(LP-1(O)_σ_ → σ*(O–H)] and *E*[(LP-2(O)_σ_ → σ*(O–H)] are the energy of corresponding hyperconjugative interactions.

Equations (3a)–(6a) suggest that an enhancement in the *ρ*_BCP_ values and *V*_BCP_ absolute values are associated with a magnification in the strength of the LP-1,2 (O)_σ_ → σ*(O–H) hyper conjugative interactions in the σ-system of a molecule.

The LP-1,2 (O)_σ_ → σ*(O–H) hyper conjugative interactions have been recognized [42,73,74] to be accompanied by a diminution in the n(LP-1) and n(LP-2) orbital populations of both oxygen lone pairs and growth in the electronic population of the antibonding σ*(O–H) orbital due to a charge transfer from the former orbital to the latter one. The n(LP-1) and n(LP-2) values are also given in Table S67. It turned out that a gain in the *ρ*_BCP_ value and *V*_BCP_ absolute value is directly proportional to the reduction in the sum of the n(LP-1) and n(LP-2) orbital occupancies of both the oxygen lone pairs and to the increase in the n[σ*(O–H)] electron occupancy of the antibonding σ*(O–H) orbital for the sample of compounds under discussion [see Equations (7a)–(10a)]:

*ρ*_BCP_ = 0.694 × <n[σ*(O–H)]> + 0.0155, r = 0.949, n = 21 (7a)

*ρ*_BCP_ = 0.694 × <n[σ*(O–H)]> + 0.0155, r = 0.949, n = 21 (8a)

*ρ*_BCP_ = −0.508 × <Σ[n(LP-1) + n(LP-2)]> + 2.0055, r = 0.729, n = 21 (9a)

|*V*_BCP_| = −0.650 × <Σ[n(LP-1) + n(LP-2)]> + 2.5568, r = 0.725, n = 21 (10a)

where Σ[n(LP-1) + n(LP-2)] is the sum of the n(LP-1) and n(LP-2) orbital occupancies of the oxygen lone pairs.

Equations (7a)–(10a) show that an enhancement in the *ρ*_BCP_ values and *V*_BCP_ absolute values is related to an increase in the charge transfer from the oxygen lone pairs to the antibonding σ*(O–H) orbital in the σ-system of a molecule. A rather poor correlation coefficient in Equations (9a) and (10a) is due to the fact that both oxygen lone pairs, in addition to interacting with the antibonding σ*(O–H) orbital, participate in other orbital interactions, which also results in a change in the n(LP-1,2) populations.

The *r*_O···H_ distance reflects the degree of overlap between the σ-orbitals of the oxygen lone pair and the antibonding σ*(O–H) orbital due to the O–H···O=C IMHB. The shorter the *r*_O···H_ distance, the greater the degree of these orbitals overlapping. The *r*_O···H_ distances for the sample of compounds in question correlates with the energy of the LP-1,2 (O)_σ_ → σ*(O–H) hyper conjugative interactions, the sum of the n(LP-1) and n(LP-2) orbital occupancies of both the oxygen lone pairs of the C=O group and the n[σ*(O–H)] electron occupancy of the antibonding σ*(O–H) orbital [see Equations (11a)–(14a)]:

*r*_O···H_ = −0.0869 × [*E*(LP-1(O)_σ_ → σ*(O–H)] + 1.9896, r = 0.862, n = 21 (11a)

*r*_O···H_ = −0.0931 × [*E*(LP-2(O)_σ_ → σ*(O–H)] + 2.0932, r = 0.949, n = 21 (12a)

*r*_O···H_ = −6.614 × <n[σ*(O–H)]> + 2.0415, r = 0.849, n = 21 (13a)

*r*_O···H_ = −0.0869 × <Σ[n(LP-1) + n(LP-2)]> + 1.9896, r = 0.736, n = 21 (14a)

The Equations (14a)–(16a) show the same relationship between the *r*_O···H_ values and the σ-interaction parameters derived from the NBO analysis, as shown above for the *ρ*_BCP_ and *V*_BCP_ values.

Thus, the findings above indicate that the *ρ*_BCP_, *V*_BCP_ values, and *r*_O···H_ distance used in the FBA method can serve as descriptors for estimating the energy of the σ-component in the total RAHB interaction. The π-component of the RAHB interaction is related to the resonance interaction in the π-system of molecules, which arises due to the charge transfer from the π-type lone pair of the donor oxygen atom of the O–H group to the C=O double bond through the π-system of the unsaturated fragment. The presence of the π-component of the RAHB interaction is detected using the MTA method as an additional increase in the H-bond energy compared to that estimated by the FBA method [38,60].

Within the framework of the FBA method, a family of Equations (15a)–(17a) was generated:

−*E*_HB_(*r*) = −22.39 × *r*_O∙∙∙H_^2^ + 96.89 × *r*_O∙∙∙H_ − 107.44 (15a)

−*E*_HB_(*ρ*) = 191.4 × *ρ*_BCP_ – 1.78 (16a)

−*E*_HB_(*V*) = 0.277 × *V*_BCP_ + 0.45 (17a)

where *ρ*_BCP_ is in a.u., but *V*_BCP_ is in kcal/mol.

Equations (15a)–(17a) were presented in [45,56], respectively. Equation (17a) is a modified version of the well-known Espinosa-Molins-Lecomte equation [40]. The slope of the linear dependence (17a) is 0.277 instead of 0.5 as in the original equation. Preference was given to the modified version of the Espinosa-Molins-Lecomte equation due to its better agreement with the IMHB energies evaluated by the MTA method [58]. Equation (18a) was used to average the FBA method energies from Equations (15a)–(17a) and to obtain a more reliable value of −*E*_HB_(FBA) IMHB energy [37,38,45,46]:

<–*E*_HB_(FBA)> = −[*E*_HB_(*r*) + *E*_HB_(*ρ*) + *E*_HB_(*V*)]/3 (18a)

**Figure 4:**
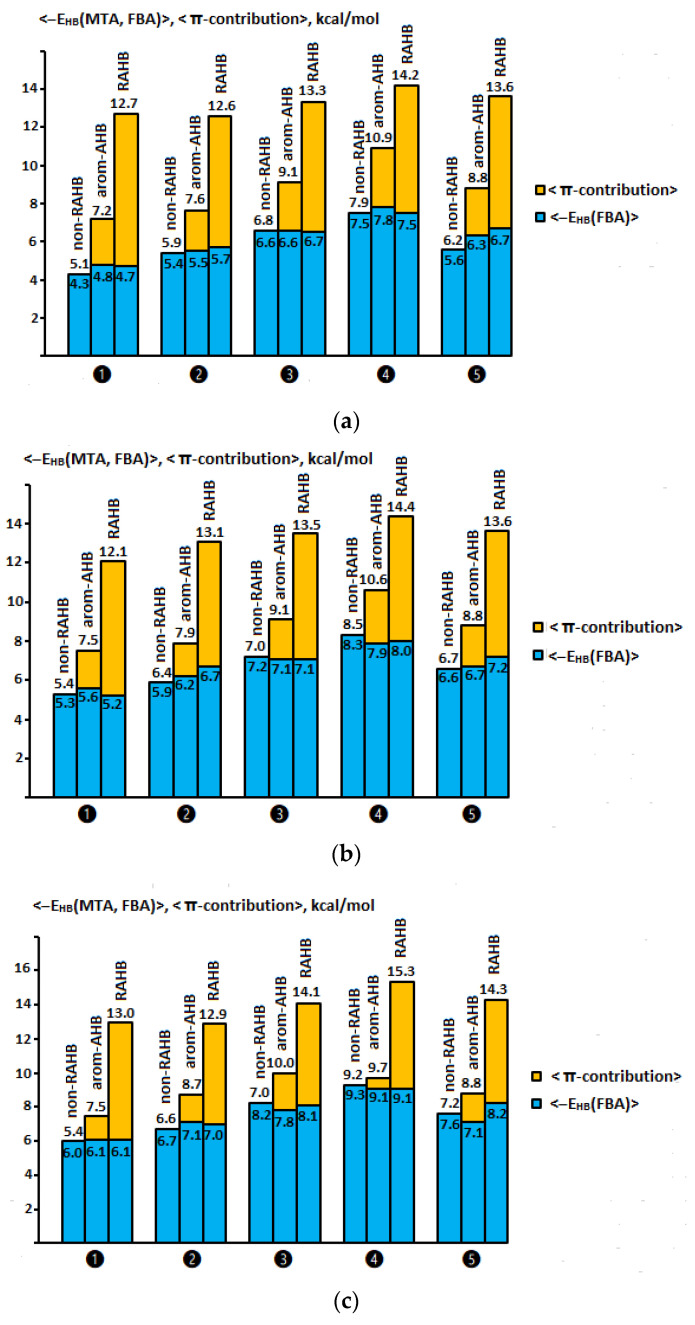
The average <−*E*_HB_(FBA)> IMHB energy estimated via the FBA method (blue) and the average <−*E*_HB_(MTA)> IMHB energy estimated via the MTA method (blue as σ-contribution and yellow as π-contribution) for compounds of the non-RAHB, arom-AHB, and RAHB clusters: range *ρ*_BCP_ and *V*_BCP_—**❶** 0.030–0.035 a.u., **❷** 0.035–0.040 a.u., **❸** 0.040–0.046 a.u., **❹** 0.046–0.051 a.u., **❺** 0.030–0.051 a.u. (total range), (**a**) and (**b**), respectively; range *r*_O∙∙∙H_—**❶** 1.75–1.80 Å, **❷** 1.70–1.75 Å, **❸** 1.65–1.70 Å., **❹** 1.60–1.65 Å, **❺** 1.60–1.80 Å (total range), (**c**).

The −*E*_HB_(FBA) energies for compounds of the non-RAHB, RAHB, and arom-AHB clusters are given in Tables S22–S66 (Supplementary Materials, pp. S34–S73). To compare the −*E*_HB_(FBA) values with the −*E*_HB_(MTA) ones, the former were averaged over the same *ρ*_BCP_, *V*_BCP_, and *r*_O···H_ subranges as the latter. The <−*E*_HB_(FBA)> magnitudes averaged within these subranges are shown in Tables S22–S66 and Figure 4a–c. As seen from the comparison of Figure 4a–c with Figure 3a–c, the ratio between the <−*E*_HB_(FBA)> values for compounds of the non-RAHB, RAHB, and arom-AHB clusters radically differs from the ratio between the <−*E*_HB_(MTA)> values in the same clusters. The <−*E*_HB_(FBA)> values are similar for all three clusters under consideration because these values are an estimate of only the σ-component of the O–H∙∙∙O=C IMHB. The difference between the <−*E*_HB_(FBA)> and <−*E*_HB_(MTA)> values for compounds of the non-RAHB cluster randomly varies within 1 kcal/mol. However, the <−*E*_HB_(FBA)> values for compounds of the arom-AHB and RAHB clusters are smaller than the <−*E*_HB_(MTA)> values by 0.6–3.1 and 5.9–8.0 kcal/mol, respectively, revealing the presence of π-contribution in the arom-AHB and RAHB interactions. At the same time, these data indicate that the π-contribution to the arom-AHB interaction is much lower than that to the RAHB interaction.

The emergence of the π-contribution of 2–3 kcal/mol to the total energy of IMHB on going from non-RAHB structures to arom-AHB ones agrees with the higher energy of the intermolecular H-bond for dimers of the aromatic heterocycles compared with the saturated analogues [29,31,32,33].

### 2.7. Comparison of π-Contributions to the Total Energy of Arom-AHB and RAHB Interactions

**Figure 5:**
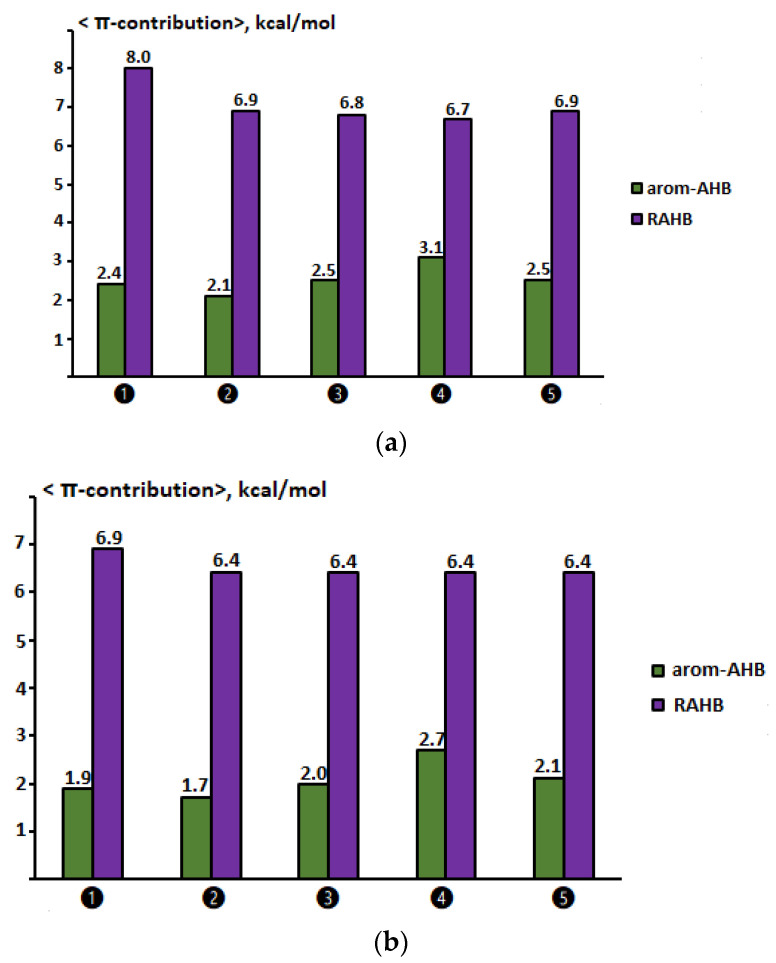

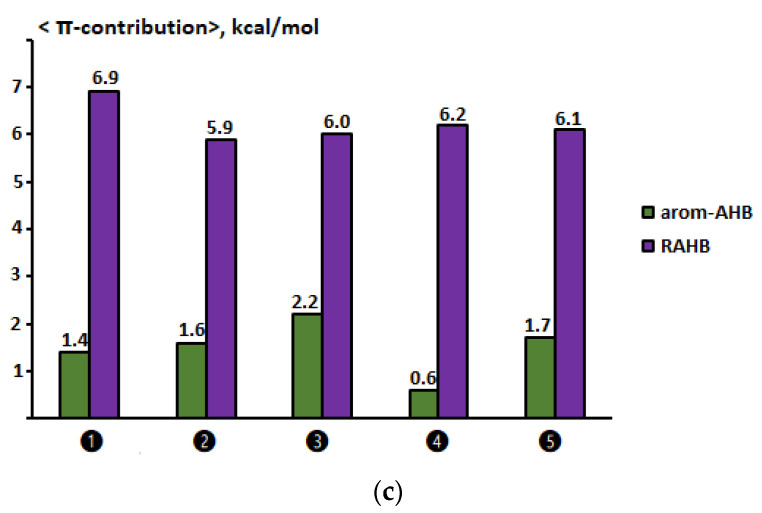
The average value of the π-contribution for the total hydrogen bond energy for compounds of the arom-AHB and RAHB clusters: range *ρ*_BCP_ and *V*_BCP_—**❶** 0.030–0.035 a.u., **❷** 0.035–0.040 a.u., **❸** 0.040–0.046 a.u., **❹** 0.046–0.051 a.u., **❺** 0.030–0.051 a.u. (total range), (**a**) and (**b**), respectively; range *r*_O∙∙∙H_—**❶** 1.75–1.80 Å, **❷** 1.70–1.75 Å, **❸** 1.65–1.70 Å., **❹** 1.60–1.65 Å, **❺** 1.60–1.80 Å (total range), (**c**).

The π-contribution to the arom-AHB and RAHB interactions can be estimated as the difference between the <−*E*_HB_(MTA)> and <−*E*_HB_(FBA)> values [38]. The values of the π-contribution for compounds of the arom-AHB and RAHB clusters within the considered range of the *ρ*_BCP_, *V*_BCP_, and *r*_O···H_ parameters are shown in Figure 5a–c and Tables S37–S66 (Supplementary Materials, pp. S40–S73). As follows from Figure 5a–c, the values of the π-contribution for the arom-AHB interaction are much lower compared with those of the RAHB interaction. The average value of the π-contribution for the arom-AHB interaction is 2.0 kcal/mol, while for the RAHB one it is 6.6 kcal/mol. Thus, the value of the π-contribution increases by almost three and a half times on going from the arom-AHB to RAHB interaction.

The main difference between the arom-AHB and RAHB interactions is that the resonance effect is transmitted through the aromatic moiety in the former case, while this effect is transmited across the olefinic bridge in the latter case. Hence, the most likely reason for the sharp decrease in the π-contribution to the arom-AHB interaction compared to the RAHB one can be the poor transfer of the resonance effect across the aromatic ring compared to the olefinic fragment. In order to explicitly compare the ability of an aromatic ring and a double bond to transmit a resonance effect, it is necessary to use the para-, meta-disubstituted benzenes and *E*-isomers of olefinic compounds.

### 2.8. Testing the Ability of the Aromatic Ring and the Double Bond to Conduct the Resonance Effect

To test and compare the ability of the aromatic ring and the olefin moiety to conduct the resonance effect, the *Don*-π-*Acc* systems were used, where the *Don* unit is the pyrrole ring, the *Acc* unit is the C=O group and the π-bridge is the phenyl ring or double bond. It is shown that the PPE arising from the addition of electron-donating and electron-withdrawing substituents to conjugated molecules significantly changes their properties in the ground and excited states [75,76,77]. The PPE energy, which is quantified using the MTA method [70,71], can serve as a measure of the conductivity of the resonance effect. The PPE strength can be varied by attaching the R_1_ substituent to the *Don* unit and the R_2_ substituent to the *Acc* unit. To test the conductivity of the resonance effect, compounds **1–54** were divided into three subseries, each of which consists of parts **a** and **b** (see Table 1). The double bond serves as the π-bridge in subseries **Ia** and **b**, while the para- and meta-disubstituted phenyl ring acts as the π-bridge in subseries **IIa,b** and **IIIa,b**, respectively. The R_1_ substituent varies, and the R_2_ one is fixed in part **a** in subseries **I**, **II**, and **III**. The R_1_ substituent is fixed, and the R_2_ one varies in part **b** of these subseries.

A quantitative criterion for the PPE strength is the −*E*_π_(PPE) value, and the higher the −*E*_π_(PPE) value, the stronger the PPE and vice versa [70,71]. The PPE strength was controlled by varying the R_1_ and R_2_ substituents with known *σ*_p_ Hammett constants [78]. The R_1_ and R_2_ substituents are given in Table 1 and their *σ*_p_ Hammett constants are shown in Table S68 (Supplementary Materials, p. S75). The −*E*_π_(PPE) values are known to reflect the structural and spectral properties of molecules, as well as their reactivity. The double bonds of the π-spacer between the *Don* and *Acc* units lengthen, while the single bonds shorten, and the frequency of the C=O vibration decreases with increasing the −*E*_π_(PPE) value [70,71]. The HOMO and LUMO energies lower, and the HOMO-LUMO energy gap narrows as the −*E*_π_(PPE) value enhances [71].

**Figure 6:**
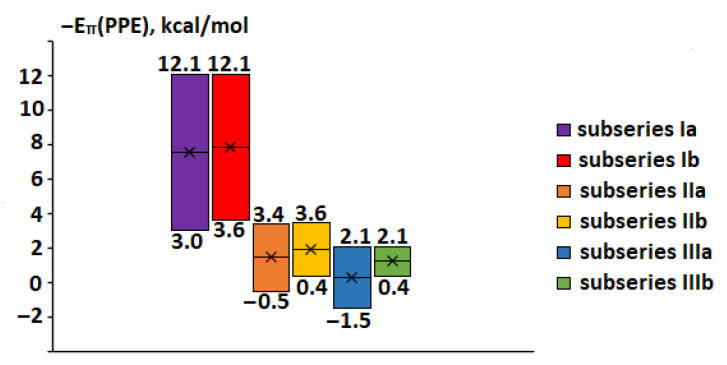
Ranges of changes for the −*E*_π_(PPE) values: subseries **Ia,b**; **IIa,b**, and **IIIa,b**—double bond, para-phenyl ring, and meta-phenyl ring, respectively, as the π-bridge. The R_1_ substituent is fixed and the R_2_ substituent varies in part **a** of the subseries, the R_2_ substituent is fixed and the R_1_ substituent varies in part **b** of the subseries.

Figure 6 shows the total ranges of changes in the −*E*_π_(PPE) values for subseries **Ia,b**–**IIIa,b** of compounds **1**–**54**. As follows from Figure 6, the ranges of change in the −*E*_π_(PPE) values for subseries **Ia,b** shift significantly towards larger magnitudes with respect to those of **IIa,b** and **IIIa,b** (3.0–12.1, 3.6–12.1 vs. –0.5 ÷ 3.3, 0.4–3.6 and –1.5 ÷ 2.1, and 0.4–2.1 kcal/mol, respectively). It suggests that the phenyl ring as a π-bridge conducts the push-pull effect much worse compared to the double bond.

The −*E*_π_(PPE) values were shown [70,71] to be linearly dependent on the *σ*_p_ values. The −*E*_π_(PPE) values in subseries **Ia,b**–**IIIa,b** of compounds **1**–**54** also reveal reliable linear dependence on the *σ*_p_ Hammett constants in form (19a):

−*E*_π_(PPE) = *A* × *σ*_p_ + *B* (19a)

where the *A* slope coefficients characterize the sensitivity of the PPE strength to the *σ_p_* Hammett constants, and the larger the *A* value, the higher the sensitivity; the *B* intersection points reveal the PPE strength for this subseries at zero value of the *σ_p_* Hammett constant.

**Figure 7:**
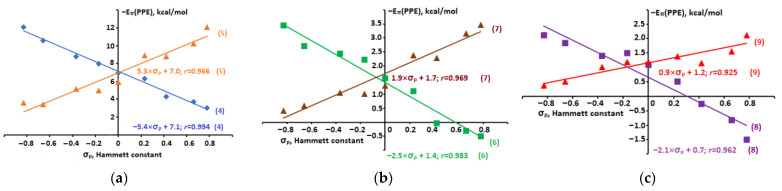
Dependence of the −*E*_π_(PPE) values on the *σ*_p_ Hammett constants for subseries **Ia,b** with the double bond as the π-bridge (**a**), subseries **IIa,b** with the para-phenyl ring as the π-bridge (**b**) and subseries **IIIa,b** with the meta-phenyl ring as the π-bridge (**c**).

**Figure 8:**
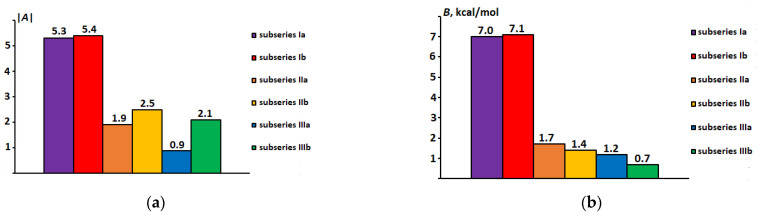
Slope *A* (**a**) and intersection point *B* (**b**) in equations −*E*_π_(PPE) = *A* × σ_p_ + *B* for subseries of compounds with different bridges: subseries **Ia,b; IIa,b** and **IIIa,b**—double bond, para-phenyl ring, and meta-phenyl ring, respectively, as the π-bridge. The R_1_ substituent is fixed and the R_2_ substituent varies in part **a** of the subseries, the R_2_ substituent is fixed and the R_1_ substituent varies in part **b** of the subseries.

Equations (4)–(9) given in Figure 7a–c show the linear dependence of the –*E*_π_(PPE) values on the *σ*_p_ Hammett constants for subseries **Ia,b**–**IIIa,b** of compounds **1**–**54**. Equations (4), (6), and (8) indicate that the –*E*_π_(PPE) energy decreases with an increase in the electron-withdrawing property of the R_1_ substituent attached to the *Don* unit of the *Don*-π-*Acc* system. Equations (5), (7), and (9) reveal that the –*E*_π_(PPE) energy augments with an increase in the electron-withdrawing property of the R_2_ substituent attached to the *Acc* unit of the *Don*-π-*Acc* system. Analysis of the *A* and *B* coefficients in Equations (4)–(9) shown in Figure 8a,b, respectively, allows one to compare the conductivity of PPE between the phenyl ring and the double bond as the π-bridges in the *Don*-π-*Acc* system. Figure 8a demonstrates that the absolute value of *A* coefficient in Equations (4) and (5) for subseries **Ia,b** is significantly higher compared to that of Equations (6)–(9) for subseries **IIa,b** and **IIIa,b**, respectively, (5.3 and 5.4 vs. 1.9, 2.5 and 0.9, 2.1, respectively). This means that the sensitivity of the PFE strength towards the substituent electronic effect, which is defined by the value of the *σ*_p_ Hammett constant, is by several times higher for the double bond as a π-bridge compared to the phenyl ring. Figure 8b displays that the *B* coefficient in Equations (4) and (5) for subseries **Ia,b** is also much larger in comparison with that of Equations (6)–(9) for subseries **IIa,b** and **IIIa,b**, respectively, (7.0 and 7.1 vs. 1.7, 1.4 and 1.2, 0.7, respectively). It follows that the PPE strength in the *Don*-π-*Acc* system, without taking into account the influence of the electronic effect of substituents at the *Don* and *Acc* units, is much higher again by several times, when the π-bridge is a double bond rather than a phenyl ring.

The above findings indicate that the phenyl ring is a poor PPE conductor with respect to the double bond. Therefore, the transmission of the resonance effect through the phenyl ring is a hindrance. This implies that the phenyl ring does not exert the same pronounced resonance effect assisting the hydrogen bond as the double bond. This explains the decrease in the π-contribution to the total energy on going from the arom-AHB to RAHB interaction by three and a half times. Additionally, this confirms the need to include the arom-AHBs into a separate class of hydrogen bonds, which differ conspicuously from a usual RAHBs and are closer to ordinary hydrogen bonds without resonance assistance.

### 2.9. Relationship of −E_π_(PPE) Values with Structural and Spectral Parameters and Frontier Orbitals Energy

This section shows that the PPE strength reflects real structural changes in the studied molecules, as well as changes in their spectral characteristics and reactivity. The π-spacer bonds lengths (double bond or phenyl ring) between the pyrrole ring and C=O group, the C=O vibrational frequency, the HOMO and LUMO energies [*E*(HOMO) and *E*(LUMO)], and the HOMO-LUMO energy gap [Δ*E*(HOMO-LUMO)] were chosen as the *P* parameter characterizing the molecular properties. The dependence of the *P* parameter on the −*E*_π_(PPE) values was established in the form of the linear relation (20a):

*P* = *A* × [−*E*_π_(PPE)] *+ B* (20a)

where *P* is listed above bond lengths, vibrational frequency, and molecular orbital energy.

The *A* and *B* coefficients of dependence (20a) of the π-spacer bond lengths, *ν*_C=O_ vibration frequency, and the molecular orbital energies on the −*E*_π_(PPE) values are given in Tables S69–S71, respectively (Supplementary Materials, pp. S75–S76).

#### 2.9.1. Dependence of π-Spacer Bonds Lengths on −E_π_(PPE) Values

**Scheme 4:**
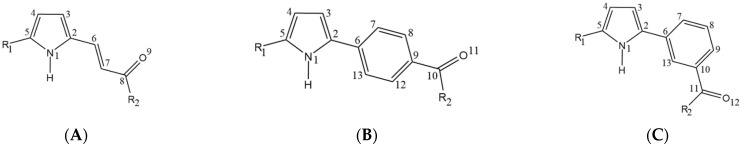
Atom numbering for compounds **1** (**A**), **19** (**B**), and **37** (**C**).

The positive coefficient *A* in Equation (20a) means an elongation of the corresponding covalent bond, while the negative one means its shortening. One can anticipate that an increase in the −*E*_π_(PPE) values for the studied compounds, reflecting an enhancement in the strength of conjugation, is accompanied by the contraction of the single bonds and the elongation of the double bonds [79]. Indeed, the C_2_–C_6_ and C_7_–C_8_ single bonds are shortened, whereas the C_6_=C_7_ and C_8_=O_9_ double bonds are lengthened with the −*E*_π_(PPE) increasing in subseries **Ia** and **b** of studied compounds (see structure **A** in Scheme 4).

**Figure 9:**
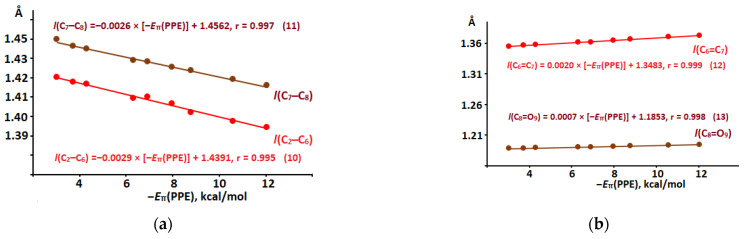
Dependencies of the *l*(C_2_–C_6_) and *l*(C_7_–C_8_) bond length on −*E*_π_(PPE) (**a**) and *l*(C_6_=C_7_) and *l*(C_8_=O_9_) bond length on −*E*_π_(PPE) (**b**) for subseries **Ia** and **b** of the studied compounds.

These trends are reliably displayed by the dependencies (10)–(13) on Figure 9a,b, dependencies (S1) and (S3) of *l*(C_2_–C_6_) and *l*(C_7_–C_8_) on −*E*_π_(PPE) from Table S69, in which the *A* slope is negative, and the dependency (S2) of *l*(C_6_=C_7_) on −*E*_π_(PPE) with the positive *A* (see Table S69). The lengths of the C_8_=O_9_ double bond do not correlate with the −*E*_π_(PPE) for subseries **IIa**. This can be the case due to through-space interaction between the C_8_=O_9_ bond orbitals with orbitals of the neighboring R_2_ substituent [70].

The C_2_–C_6_, C_7_–C_8_, C_9_–C_10_, and C_12_–C_13_ bonds are shortened, the C_6_–C_7_, C_8_–C_9_, C_10_=O_11_, C_9_–C_12_, and C_6_–C_13_ bonds (see structure **B** in Scheme 4) are lengthened with the −*E*_π_(PPE) increasing in subseries **IIa** and **b** (negative *A* coefficient in Equations (S4), (S6), (S8), (S11), (S13), (S15), (S17) and (S19), positive *A* coefficient in Equations (S5), (S7), (S9), (S10), (S12), (S14), (S16) and (S18), Table S69). The C_2_–C_6_, C_7_–C_8_, and C_10_–C_11_ bonds are shortened, the C_6_–C_7_, C_8_–C_9_, C_9_–C_10_, C_10_–C_13_, and C_6_–C_13_ bonds (see structure **C** in Scheme 4) are lengthened with the –*E*_π_(PPE) increasing in subseries **IIIa** (negative *A* coefficient in equations (S21), (S23), and (S26), positive *A* coefficient in Equations (S22), (S24), (S25), (S27), and (S28), Table S69). The C_2_–C_6_, and C_10_–C_11_ bonds are shortened with the −*E*_π_(PPE) increasing in subseries **IIIb** (negative *A* coefficient in Equation (S29) and (S30), Table S69). No correlation of other bond lengths with the −*E*_π_(PPE) value is observed in subseries **IIIb**. It should be recalled that there are no single and double bonds inside the phenyl ring due to the electronic delocalization.

#### 2.9.2. Dependence of C=O Vibration Frequency on −E_π_(PPE) Value

**Figure 10:**
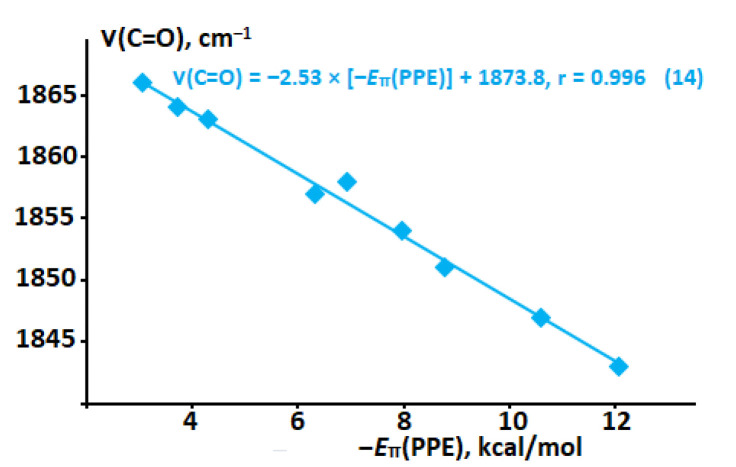
Dependencies of the *ν*_C=O_ vibrational frequency on −*E*_π_(PPE) for subseries **Ia** of the studied compounds.

Strengthening conjugation within the molecules under study should cause the re shift of the *ν*_C=O_ vibration frequency [79]. The decrease in the *ν*_C=O_ values with the −*E*_π_(PPE) enhancement takes place in subseries **Ia**, **IIa**, and **IIIa** of the studies compounds. This regularity is shown by Equation (14) in Figure 10 and dependencies (S31) and (S32) of *ν*_C=O_ on −*E*_π_(PPE) from Table S70, in which the *A* slope is negative.

Similarly to the C=O bond length, the *ν*_C=O_ values do not show a correlation with the −*E*_π_(PPE) values in the subseries **Ib**, **IIb**, and **IIIb** of the studies compounds for the same reason.

#### 2.9.3. Dependence of HOMO and LUMO Energy on −E_π_(PPE) Value

**Figure 11:**
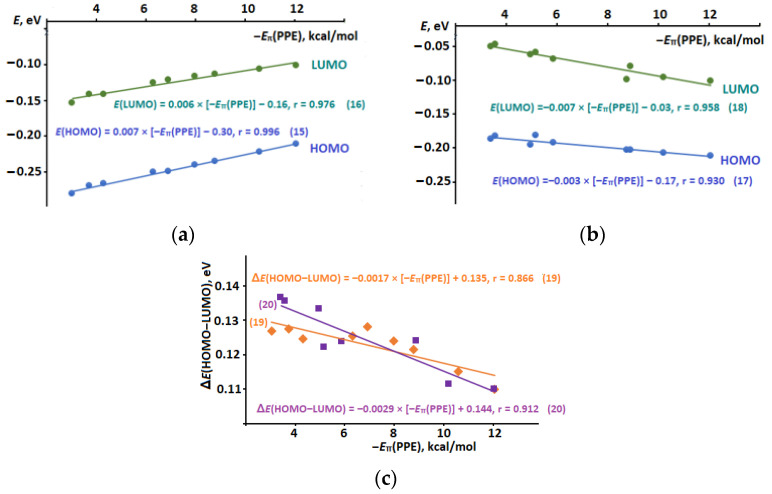
Dependencies of the *E*(HOMO) and *E*(LUMO) energies on −*E*_π_(PPE) for subseries **Ia** (**a**) and **Ib** (**b**); dependency of Δ*E*(HOMO–LUMO) energy gap on −*E*_π_(PPE) for subseries **Ia,b** (**c**) of studies compound.

The *E*(HOMO) and *E*(LUMO) energies of frontier molecular orbitals are an important characteristic of the orbital structure, since they reflect the ability of a molecule to donate and accept electrons. The data obtained recognize that the *E*(HOMO) and *E*(LUMO) values vary in a regular manner with a change in the −*E*_π_(PPE). The *E*(HOMO) and *E*(LUMO) energies rise with the −*E*_π_(PPE) growth in subseries **Ia**, **IIa**, and **IIIa** while the *E*(HOMO) and *E*(LUMO) energies lower with the −*E*_π_(PPE) increase in subseries **Ib**, **IIb**, and **IIIb**. These tendencies are shown by Equations (15)–(18) on Figure 11a,b, Equations (S33), (S34), (S39), and (S40) with the positive *A* coefficient and Equations (S36), (S37), (S42), and (S43) with the negative *A* coefficient (Table S71). However, the Δ*E*(HOMO–LUMO) energy gap narrows in all studied subseries **Ia**,**b**–**IIIa**,**b**. It follows from Equations (19) and (20) on Figure 11c and Equations (S35), (S38), (S41), and (S44) from Table S71 having negative *A* slope. This result is of special importance since the optical (absorption, fluorescent, phosphorescent) properties of molecules depend on the Δ*E*(HOMO–LUMO) energy gap value.

## 3. Methods and Computational Details

**Scheme 5:**
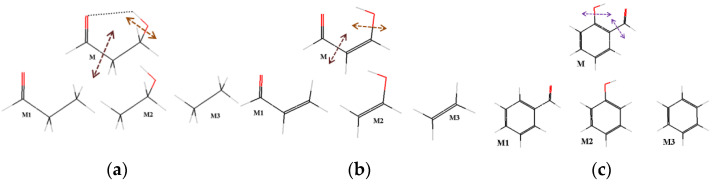
Fragmentation scheme for the *E*_HB_(MTA) value calculation in compounds with the non-RAHB (**a**), RAHB (**b**), and arom-AHB (**c**).

The energy of the O−H···O=C IMHB and PPE in the studied compounds was estimated in accordance with the following fragmentation schemes. For both cases, the entire M molecule is the main “fragment”. The M1 fragment presents the M molecule without the H-bond donor, while the M2 fragment is the M molecule without the H-bond acceptor. As the excess atoms appear when the M1 and M2 fragments are superimposed, an additional M3 fragment is introduced to compensate for them (see Scheme 5a–c for compounds with the non-RAHB, RAHB, and arom-AHB, respectively). At the cutting site of the entire M molecule, the hydrogen atoms are placed at a distance of 1.1 Å from the corresponding carbon atom (see [48,56] for more details). The *E*_HB_(MTA) values of the IMHB energy obtained via the MTA method are calculated by Equation (21a):

*E*_HB_(MTA) = [*E*(M) + *E*(M3)] − [*E*(M1) + *E*(M2)] (21a)

where the *E*(M), *E*(M1), *E*(M2), and *E*(M3) values are the energy of the entire M molecule and the M1, M2, and M3 fragments, respectively.

**Scheme 6:**
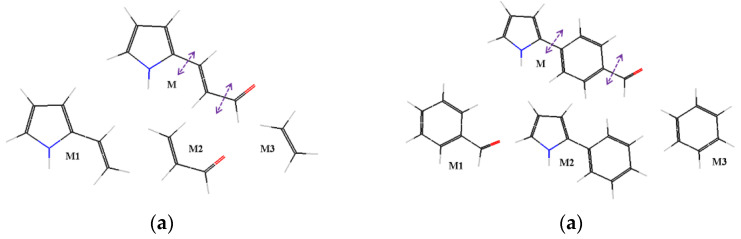
Fragmentation scheme for the *E*_π_(PPE) value calculation for compound with the double bond as π-bridge (**a**) and the phenyl ring as π-bridge (**b**).

When calculating the PPE energy, the fragmentation scheme looks similar. The entire molecule exhibiting the PPE phenomena is the *Don*-π-*Acc* system, in which intramolecular charge transfer from the *Don* unit to the *Acc* unit via the π-linker takes place (more details in [70,71]). The M molecule without the *Don* unit forms the M1 fragment, whereas the M molecule without the *Acc* unit creates the M2 fragment. To compensate for excess atoms due to the overlap of the M1 and M2 fragments, an additional M3 fragment is again introduced (see Scheme 6a,b for a compound with the double bond as π-bridge and the phenyl ring as π-bridge, respectively.

Similarly to the IMHB calculating, at the cutting site of the entire M molecule, the hydrogen atoms are placed at the distance of 1.1 Å from the corresponding carbon atom. By analogy with the calculation of the IMHB energy, the *E*_π_(PPE) values of the PPE energy are calculated by Equation (22a):

*E*_π_(PPE) = [*E*(M) + *E*(M3)] − [*E*(M1) + *E*(M2)] (22a)

where the *E*(M), *E*(M1), *E*(M2), and *E*(M3) values are the energy of the entire M molecule and the M1, M2, and M3 fragments, respectively.

As both the IMHB and PPE interactions make a negative contribution to the total energy of the molecule, the *E*_HB_(MTA) and *E*_π_(PPE) values are always negative. For the sake of simplifying the discussion of the results, we use the inverse value of the *E*_HB_(MTA) and *E*_π_(PPE) parameters from Equations (21a) and (22a), respectively, throughout the article, i.e., −*E*_HB_(MTA) and −*E*_π_(PPE) values. Thus, an increase in the −*E*_HB_(MTA) and −*E*_π_(PPE) values corresponds to the IMHB and PPE strengthening and *vice versa*.

The Gaussian 09 program package [80] (Revision B.01; Gaussian Inc., Wallingford, CT, USA) was used to carry out the calculations. Initially, the geometries of the M molecules were optimized at the B3LYP/6–311++G(d,p) level. After that, the M molecules were cut into M1, M2, and M3 fragments without changing the optimized geometry of the entire M molecules. As the last step, the single-point energy calculations at the level MP2(full)/6–311++G(2d,2p) of the entire M molecule and M1, M2, and M3 fragments were used for the energy correction of geometries optimized at the B3LYP level. In order to make sure that a local minimum of energy was found, the vibrational frequencies were checked for the absence of imaginary ones. The *ρ*_BCP_ and V_BCP_ topological properties from Bader quantum theory “Atoms in Molecules” (OTAIM) [81] were calculated using the AIMAll program package [82]. NBO characteristics were obtained through the NBO program implemented in the Gaussian 09 at MP2/6-311++G(2d,2p) level. All calculations were carried out for the gas phase.

## 4. Conclusions

Using the molecular tailoring approach, intramolecular hydrogen bonds energy has been quantitatively compared between the ortho-disubstituted benzenes, where the hydrogen bond donor and hydrogen bond acceptor are separated by an aromatic moiety (arom-AHB interaction), and the *Z*-olefins, where the hydrogen bond donor and hydrogen bond acceptor are separated by a double bond (RAHB interaction). The energy of the arom-AHB interaction is shown to be significantly less than the energy of RAHB interaction. The total energy of these interactions is divided into the σ- and π-components using molecular tailoring and function-based approaches. Although the σ-components of the arom-AHB and RAHB interactions are the same, the π-component of the former is almost three and a half times smaller than that of the latter. The reason for the sharp decrease in the π-contribution to the arom-AHB interaction is the poor conductivity of the resonance effect by the aromatic ring compared to the double bond. The molecular tailoring approach suggests that the push-pull effect as part of the resonance effect is poorly conducted in the para- and meta-disubstituted benzenes as compared with the *E*-olefins. The energy of the push-pull effect is several times lower for the *Don*-π-*Acc* systems, where the π-bridge is the aromatic ring instead of the double bond. The sensitivity of the push-pull energy to the electronic effect of substituents at the *Don* and *Acc* units is also lower by several times in the case of the aromatic ring as the π-bridge than that of the double bond. These data reveal that the aromatic ring cannot provide the effective transfer of the resonance effect, which results in a sharp decrease in the π-component of the arom-AHB interaction and a reduction in the total energy of the arom-AHB interaction relative to the RAHB one. It makes the arom-AHB interaction more similar to a conventional hydrogen bond than to the RAHB interaction.

## Figures and Tables

**Table 1:** Studied subseries **Ia,b**–**IIIa,b** of compounds **1–54** and −*E*_π_(PPE) values for them (in kcal/mol).

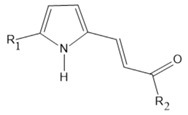			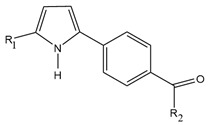			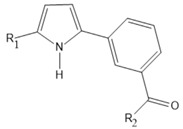		
subseries **Ia,b**			subseries **IIa,b**			subseries **IIIa,b**		
compounds **1–18**			compounds **19–36**			compounds **37–54**		
**subseries Ia,** **R_2_ = NO_2_, R_1_ varies**			**subseries IIa,** **R_2_ = NO_2_, R_1_ varies**			**subseries IIIa,** **R_2_ = NO_2_, R_1_ varies**		
**No**	**R_1_**	**−*E*** **_π_** **(PPE)**	**No**	**R_1_**	**−*E*** **_π_** **(PPE)**	**No**	**R_1_**	**−*E*** **_π_** **(PPE)**
**1**	N(CH_3_)_2_	12.06	**19**	N(CH_3_)_2_	3.44	**37**	N(CH_3_)_2_	2.12
**2**	NH_2_	10.60	**20**	NH_2_	2.71	**38**	NH_2_	1.84
**3**	OH	8.79	**21**	OH	2.43	**39**	OH	1.39
**4**	CH_3_	7.98	**22**	CH_3_	2.22	**40**	CH_3_	1.50
**5**	H	6.92	**23**	H	1.56	**41**	H	1.09
**6**	Cl	6.31	**24**	Cl	1.11	**42**	Cl	0.51
**7**	CHO	4.31	**25**	CHO	–0.05^a^	**43**	CHO	–0.26 ^a^
**8**	CN	3.73	**26**	CN	–0.30^a^	**44**	CN	–0.82 ^a^
**9**	NO_2_	3.04	**27**	NO_2_	–0.51^a^	**45**	NO_2_	–1.50 ^a^
**subseries Ib,** **R_1_ = N(CH_3_)_2_, R_2_ varies**			**subseries IIb,** **R_1_ = N(CH_3_)_2_, R_2_ varies**			**subseries IIIb,** **R_1_ = N(CH_3_)_2_, R_2_ varies**		
**No**	**R_2_**	**−*E*** **_π_** **(PPE)**	**No**	**R_2_**	**−*E*** **_π_** **(PPE)**	**No**	**R_2_**	**−*E*** **_π_** **(PPE)**
**10**	N(CH_3_)_2_	3.59	**28**	N(CH_3_)_2_	0.40	**46**	N(CH_3_)_2_	0.37
**11**	NH_2_	3.42	**29**	NH_2_	0.59	**47**	NH_2_	0.50
**12**	OH	5.17	**30**	OH	1.05	**48**	OH	1.02
**13**	CH_3_	4.96	**31**	CH_3_	1.01	**49**	CH_3_	1.19
**14**	H	5.88	**32**	H	1.30	**50**	H	1.19
**15**	Cl	8.90	**33**	Cl	2.38	**51**	Cl	1.39
**16**	CHO	8.77	**34**	CHO	2.28	**52**	CHO	1.15
**17**	CN	10.22	**35**	CN	3.15	**53**	CN	1.56
**18**	NO_2_	12.06	**36**	NO_2_	3.45	**54**	NO_2_	2.12

^a^ A negative −*E*_π_(PPE) values are related to the *anti*-push-pull effect manifestation [71].

## Data Availability

The data presented in this work are available in the article and Supplementary Materials.

## Supplementary Materials

The following supporting information can be downloaded at: https://www.mdpi.com/article/10.3390/molecules28020536/s1, Scheme S1: Fragmentation scheme for the *E*_HB_(MTA) value calculation in compounds with bifurcated H-bond; Tables S1–S7: Structure of compounds from the non-RAHB cluster; Tables S8–S10: Structure of compounds from the RAHB cluster; Table S11–S15: Structure of compounds from the arom-AHB cluster; Table S16–S18: Characteristics of the O–H∙∙∙O=C hydrogen bond for studied compounds from the non-RAHB, RAHB and arom-AHB clusters; Tables S19–S21: The calculated E*_π_*(PPE) energy, bond length, vibration frequency, HOMO and LUMO energy, HOMO–LUMO energy gap for studied compounds; Tables S22–S36: The *E*_HB_(MTA) and *E*_HB_(FBA) hydrogen bond energy assessed via molecular tailoring and function-based approaches for compounds from non-RAHB cluster within the different ranges of *r*_O∙∙∙H_, *ρ*_BCP_ and *V*_BCP_ parameters; Tables S37–S51: The *E*_HB_(MTA) and *E*_HB_(FBA) hydrogen bond energy assessed via molecular tailoring and function-based approaches for compounds from RAHB cluster within the different ranges of *r*_O∙∙∙H_, *ρ*_BCP_ and *V*_BCP_ parameters; Tables S52–S66: The E_HB_(MTA) and E_HB_(FBA) hydrogen bond energy assessed via molecular tailoring and function-based approaches for compounds from arom-AHB cluster within the different ranges of *r*_O∙∙∙H_, *ρ*_BCP_ and *V*_BCP_ parameters; Table S67: NBO characteristics for studied compounds; Table S68: The R_1_ and R_2_ substituents and their Hammett constants for the studied compounds; Tables S69–S71: Parameters of linear dependencies of bond length, vibrational frequency, HOMO and LUMO energy on E*_π_*(PPE) values; Atoms coordinates for newly calculated compounds.

## Sample Availability

Samples of the compounds are not available from the authors.
