# Supplementary material for: Revealing the Reasons for Degeneration of Resonance-Assisted Hydrogen Bond on the Aromatic Platform: Calculations of Ortho-, Meta-, Para-Disubstituted Benzenes, and (Z)-(E)-Olefins
Source: Molecules. 2023 Jan 5;28(2):536. doi: 10.3390/molecules28020536 (PMC9860835; doi:10.3390/molecules28020536)
Supplement: Supplementary file 1 [file molecules-28-00536-s001.zip › molecules-2015324-supplementary.pdf]

Supplementary material for

Revealing the Reasons for Degeneration of Resonance-Assisted Hydrogen Bond on the Aromatic Platform: Calculations of ortho-, meta-, para-Disubstituted Benzenes and (*Z*)-(*E*)-Olefins

Andrei V. Afonin,[1] Danuta Rusinska-Roszak[2]

[1]*Siberian Division of Russian Academy of Sciences, A. E. Favorsky Irkutsk Institute of Chemistry, 664033 Irkutsk, Russia; E-mail: andvalaf@irioch.irk.ru*

[2]*Institute of Chemical Technology and Engineering, Poznan University of Technology, Pl. M. Sklodowskiej-Curie 2, 60–965 Poznan, Poland; Email: danuta.rusinska-roszak@put.poznan.pl*

**Contents**	**Page**

**Scheme S1.** Fragmentation scheme for the E_{HB}(MTA) value calculation in compounds with bifurcated H-bond.	S2
**Tables S1 – S7.** Structure of compounds from the non-RAHB cluster.	S3 – S9
**Tables S8 – S10.** Structure of compounds from the RAHB cluster.	S9 – S16
**Tables S11 – S15.** Structure of compounds from the arom-AHB cluster.	S17 – S20
**Tables S16 – S18**. Characteristics of the O–H···O=C hydrogen bond for studied compounds from the non-RAHB, RAHB and arom-AHB clusters.	S21 – S31
**Tables S19 – S21**. The calculated E_{π}(PPE) energy, bond length, vibration frequency, HOMO and LUMO energy, HOMO–LUMO energy gap for subseries **Ia** and **Ib**, **IIa** and **IIb**, **IIIa** and **IIIb** of studied compounds **1 – 18**, **19 – 36**, **37 – 54**.	S31 – S33
**Tables S22–S36.** The E_{HB}(MTA) and E_{HB}(FBA) hydrogen bond energy assessed via molecular tailoring and function-based approaches for compounds from non-RAHB cluster within the different ranges of $r_{O\cdots H}$, ρ_{BCP} and V_{BCP} parameters.	S34 – S40
**Tables S37–S51.** The E_{HB}(MTA) and E_{HB}(FBA) hydrogen bond energy assessed via molecular tailoring and function-based approaches for compounds from RAHB cluster within the different ranges of $r_{O\cdots H}$, ρ_{BCP} and V_{BCP} parameters.	S40 – S55
**Tables S52–S66.** The E_{HB}(MTA) and E_{HB}(FBA) hydrogen bond energy assessed via molecular tailoring and function-based approaches for compounds from arom-AHB cluster within the different ranges of $r_{O\cdots H}$, ρ_{BCP} and V_{BCP} parameters.	S55 – S73
**Table S67.**Energy of the NBO hyperconjugative interactions, the ρ_{BCP} electron density at the hydrogen bond critical point, the V_{BCP} potential energy density at the hydrogen bond critical point, the $r_{O\cdots H}$ hydrogen bond length the n(LP-1) and n(LP-2) lone pairs orbital population and the n[σ*(O–H)] bond electronic population for sampling compounds from non-RAHB, RAHB, and arom-AHB clusters.	S74
**Table S68**. The R_1 and R_2 substituents and their Hammett constants for the studied compounds **1–54**.	S75
**Table S69.** Parameters of linear dependencies (S1)–(S30), $l = A \times [-E_{\pi}(PPE)] + B$, of bond lengths on the $-E_{\pi}$(PPE) values for subseries **Ia,b – IIIa,b** of studied compounds **1 – 54.**	S75
**Table S70.** Parameters of linear dependencies (S31) and (S32), $\nu_{C=O} = A \times [-E_{\pi}(PPE)] + B$, of the C=O frequency on the $-E_{\pi}$(PPE) values for subseries **Ia,b** – **IIIa,b** of studied compounds **1** – **54**.	S76
**Table S71.** Parameters of linear dependencies (S33)–(S44), E(HOMO), E(LUMO) and ΔE (HOMO-LUMO) = $A \times [-E_{\pi}(PPE)] + B$, of molecular orbital energy on the $-E_{\pi}$(PPE) values for subseries **Ia,b** – **IIIa,b** of studied compounds **1** – **54**.	S76
**Atoms coordinates for newly calculated compounds.**	S77 – S104

a) **b)**

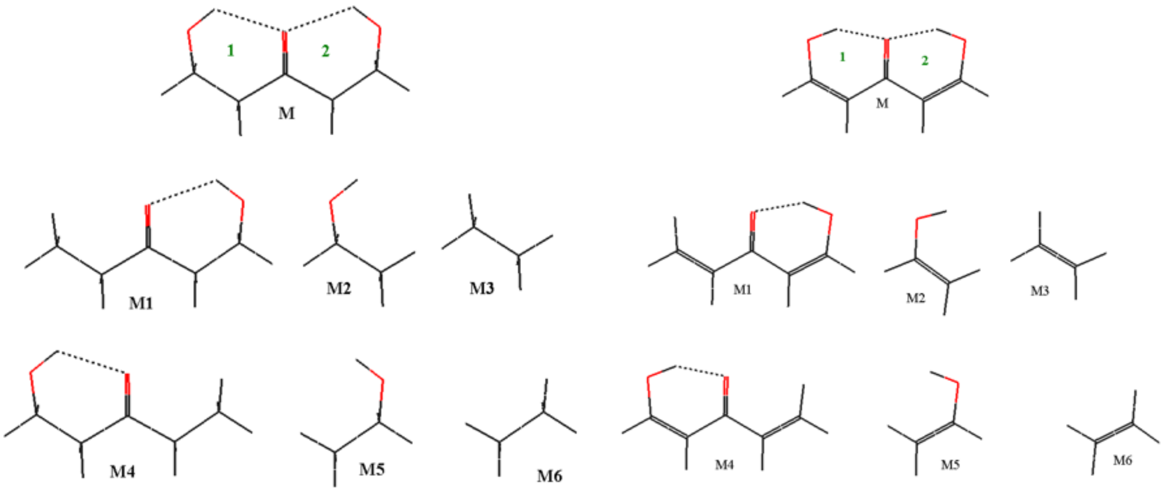

Scheme S1. Fragmentation scheme for the E_{HB}(MTA) value calculation in compounds with bifurcated H-bond: non-RAHB structure (**a**), RAHB structure (**b**).

$E_{HB}(1) = [E(M) + E(M3)] - [E(M1) + E(M2)]$

$E_{HB}(2) = [E(M) + E(M6)] - [E(M4) + E(M5)]$

Table S1. Structure of **1-non-RAHB – 43-non-RAHB** compounds. Taken from Ref.56.

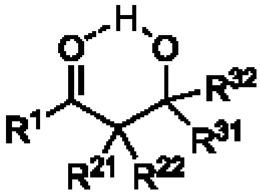

	C1[a]	C2[b,c]		C3[c,d]		*R/S*[e]	conf[f]	E_{rel}[g]	E_{HB}
	R^1	R^{21}	R^{22}	R^{31}	R^{32}				
**1**	H	H	H	H	H	–	-G g	–	**2.0**[h]
**2a**	H	H	H	Me eq	H	*R*	-G g	0.0	**2.5**
**2b**	H	H	H	H	Me ax	*S*	-G g	1.3	**1.4**
**3**	H	H	H	Me eq	Me ax	-	-G g	–	**1.9**
**4**	Me	H	H	H	H	–	-G g	–	**2.8**[i]
**5a**	Me	H	H	Me eq	H	*R*	-G g	0.0	**3.2**
**5b**	Me	H	H	H	Me ax	*R*	G -g	1.2	**2.1**
**6a**	Me	H	H	Et eq t	H	*R*	-G g	0.0	**3.3**
**6b**	Me	H	H	Et eq g	H	*R*	-G g	0.6	**3.3**
**6c**	Me	H	H	Et eq-g	H	*R*	-G g	0.6	**3.1**
**6d**	Me	H	H	H	Et ax t	*R*	G -g	1.2	**2.1**
**6d**	Me	H	H	H	Et ax g	*S*	-G g	1.4	**2.2**
**6f**	Me	H	H	H	Et ax -g	*S*	-G g	2.7	**2.3**
**7**	Me	H	H	H	CH_2CH_2OH ax t	*R*	G -g	–	**2.6**
**8a**	Me	H	H	Et eq t	Me ax	*R*	-G g	0.0	**2.7**
**8b**	Me	H	H	Me eq	Et ax g	*S*	-G g	0.2	**2.6**
**9a**	Ph	H	H	H	H	–	-G g	0.0	**2.8**
**9b**	H	H	H	Ph eq	H	*R*	G -g	5.5	**2.6**
**10a**	Ph	H	H	H	Ph ax	*R*	-G g	0.0	**3.0**
**10b**	Ph	H	H	Ph eq	H	*S*	-G g	2.3	**3.4**
**11**	Ph	H	H	H	Me eq	*R*	-G g	–	**3.2**
**12**	Me	H	H	Me eq	Me ax	–	-G g	–	**2.6**
**13a**	H	OH	H	H	H	*R*	G -g	0.0	**1.9**
**13b**	H	OH -g	H	H	H	*R*	-G g	2.1	**2.1**
**13c**	H	OH t	H	H	H	*R*	-G g	2.5	**2.1**
**14a**	OH trans	H	H	H	H	–	-G g	0.0	**2.3**
**14b**	OH cis	H	H	H	H	–	-G g	4.9	**2.5**
**15**	NH_2	H	H	H	H	–	-G g	–	**3.7**
[illegible]	[illegible]	[illegible]	[illegible]	[illegible]	[illegible]	[illegible]	[illegible]	[illegible]	[illegible]

16	CH_2CH_2OH	H	H	H	H	–	-G g	–	2.5
17a	OH trans	H	H	Me eq	H	*R*	-G g	0.0	2.7
17b	OH cis	H	H	Me eq	H	*R*	-G g	4.9	3.0
18	NH_2	H	H	Me eq	H	–	-G g	–	4.2
19a	OMe trans	H	H	Me eq	H	*R*	-G g	0.0	3.1
19b	OMe cis	H	H	Me eq	H	*R*	-G g	7.2	3.4
20	OMe trans	H	H	=O		–	c c	–	5.8
21	H	H	H	=O		–	c c	–	5.5[j]
22	Me	H	H	=O			c c	–	6.4
23	F	H	H	=O		–	c c	–	3.5[k]
24a	OH trans	H	H	=O		–	-G c	0.0	5.2[l]
24b	OH cis	H	H	=O		–	-G c	4.9	5.9[m]
25	OH trans	H	H	H	COOH	*R*	-G g	–	2.8
26	-CH=CH-CH_2-CH_2-		H	H	H	*R*	-G g	–	3.3[n]
27	H	H	-CH_2-CH=CH-CH_2-		H	*R R*	-G g	–	2.2
28	H	=CH-C(CH_2)-CH=				–	c c	–	5.6[o]
29	H	=CH-C($CHNH_2$)-CH=				–	c c	–	6.8[p]
30a	H	H	-$(CH_2)_4$-		H	*S S*	G -g	0.0	2.4
30b	H	H	-$(CH_2)_4$-		H	*S R*	-G g	0.1	2.1
31a	H	H	-CH=CH-CH=CH-		H	*S S*	G -g	0.0	2.6
31b	H	H	-CH=CH-CH=CH-		H	*S R*	-G g	1.2	1.8
32a	-CH=CH-CH=CH-		H	H	H	*S*	G -g	–	3.5
32b	-$(CH_2)_4$-		H	H	H	*R*	-G g	–	3.2
33a	OH trans	H	C_4H_3S ax	=O		–	-G c	0.0	5.0
33b	OH cis	C_4H_3S eq	H	=O		–	-G g	2.8	5.3
33c	OH cis	C_4H_3S eq	H	=O		–	-G c	2.4	5.9
33d	OH trans	H	C_4H_3S ax	=O		–	-G g	0.2	4.9
33e	OH trans	C_4H_3S eq	H	=O		–	-G g	3.4	4.6
34[q]		$C_{10}H_{12}O_2$		=O		–	c c	–	12.5
35[r]		$C_{11}H_7O$		=O		–	c c	–	11.2
36[s]		$C_{13}H_9O$		=O		–	c c	–	9.4
37a	H	=CH-OH s-cis		=O		–	c c	0.0	9.3
37b	H	=CH-OH s-trans		=O		–	c c	14.8	6.9
38[t]	OH trans	H	H	COOH eq	-CH_2-C_6H_4(OH)-	*R*	G -g	0.0	2.4
39[u]	$C_9H_{11}O_2$	H	H	H	C_5H_{11} eq	*S*	G -G	–	3.3
40[v]	-CH=CH-CO-CH=			H	H	–	-G g	–	2.8

41a[w]	-CH=C-CH$_2$(OH)-CO-CH=		H	H	–	G -g/-G g	0.0	2.8
41b[x]	-CH=CH-CO-C(CH$_2$OH)=		H	H	–	G -g/G -g	1.8	3.0
42[y]	-C$_6$H$_4$-CH$_2$-C(OH)=		=O		–	–	–	6.4
43[z]	Me	tBu	Me		–	–	–	13.7

Table S2. Structure of **44-non-RAHB – 59-non-RAHB** compounds. Taken from Ref.56.

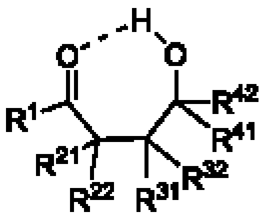

No	C1[a]	C2-C3		C4[b]		R/S[c]	conform[d]	E rel[e]	E_{HB}
	R[1]	R[21]=R[22]	R[31]=R[32]	R[41]	R[42]				
44a	H	H	H	H	H	–	-G -g	0.0	2.7
44b	H	H	H	H	H	–	-G g	0.8	3.3
45a	H	H	H	Me eq	H	*S*	G -g	0.0	3.1
45b	H	H	H	H	Me ax	*R*	G -g	0.5	3.3
46	H	H	H	Me eq	Me ax	–	-G g	–	3.1
47a	H	H	-CH=CH-CH=CH-			–	c g	0.0	4.6
47b	H	=CH-CH=CH-CH=		H	H	–	-G g	2.4	4.1
48	Me	H	H	H	H	–	-G g	–	4.0
49a	Me	H	H	Me eq	H	*R*	-G g	0.0	3.8
49b	Me	H	H	H	Me ax	*S*	-G g	0.5	3.9
50	Me	H	H	Me eq	Me ax	–	-G g	–	3.8
51	Me	=CH-CH=CH-CH=		H	H	–	-G g	–	4.7
52[f]	Me	H	H	=O		–	-G c	–	5.9
53a	OH trans	H	H	H	H	–	-G g	0.0	3.3
53b	OH cis	H	H	H	H	–	-G g	5.1	3.4
53c	OH cis	H	H	H	H	–	-G -g	3.9	2.7
54a	OMe trans	H	H	H	H	–	-G g	0.0	3.9
54b	OMe cis	H	H	H	H	–	-G g	7.8	3.7
55[g]	C$_3$H$_6$OH	H	H	H	H	–	-G g	–	4.1
[illegible]	[illegible]	[illegible]	[illegible]	[illegible]			[illegible]	[illegible]	[illegible]

56a[h]	OH trans	H	H	=O	–	-G c	0.0	5.1
56b[h]	OH cis	H	H	=O	–	-G c	5.0	5.4
57a[i]	OH trans	=CH_2	H	=O	–	-G c	0.0	5.5
57b[i]	OH trans	H	=CH_2	=O	–	-G -g	2.3	5.8
58[j]	OH trans	H	[j]	=O	*R*	c -g	1.5	5.9
59[k]	OH trans	H	[k]	=O	*S*	-G c	–	6.3

Table S3. Structure of **60-non-RAHB – 69-non-RAHB** compounds. Taken from Ref.56.

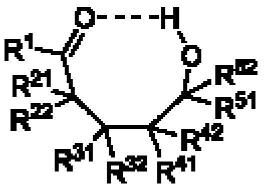

No	C1[a]	C2-C3		C4	C5		*R/S*[b]	conf[c]	E_{rel}[d]	$-E_{HB}$
	R^1	$R^{21}=R^{22}$	$R^{31}=R^{32}$	$R^{41}=R^{42}$	R^{51}	R^{52}				
60	H	H	H	H	H	H	–	-G -g	–	3.1
61	Me	H	H	H	H	H	–	-G -g	–	3.8
62	Me	H	=CH-CH=CH-CH=		H	H	–	-G -g	–	4.1
63a[e]	OH trans	H	H	H	=O		–	G c	0.0	5.9
63b[e]	OH cis	H	H	H	=O		–	G c	5.0	6.0
64a[f]	OH trans	H	-CH[C(CH_3)=CH_2]-C_3H_5-CH_2-NH-		=O		all *S*	-G c	0.0	6.2
64b[f]	OH cis	H	-CH[C(CH_3)=CH_2]-C_3H_5-CH_2-NH-		=O		all *S*	G c	6.9	6.5
64c[f]	OH cis	H	-CH[(C=CH_2)(CH_3)]-C_3H_5-CH_2-NH-		=O		all *S*	G c	7.1	6.4
65	Me	instead of C2C3C4C5 is -O-CH=CH-CO-						–	–	4.4
66[g]	Me	instead of C2C3C4C5 is -O-C_4H_4-ortho-CO-						–	–	4.4
67a	OH trans	H[h]	-CH_2-CH_2- ax-eq	H	=O		1*S* 3*R*	−G c	0.0	6.0
67b	OH cis	H[h]	-CH_2-CH_2- ax-eq	H	=O		1*S* 3*R*	−G c	5.0	6.2
68a	OH trans	H[i]	-CH_2-CH_2-CH_2- ax-ax	H	=O		1*S* 3*R*	c -g	0.0	3.7
68b	OH cis	H[i]	-CH_2-CH_2-CH_2- ax-ax	H	=O		1*S* 3*R*	c -g	5.3	4.3
69a[j]	OH trans	-$(CH_2)_4$-			=O			G c	0.0	5.3
69b[j]	OH cis	-$(CH_2)_4$-			=O			G c	5.3	5.3

Table S4. Structure of **70-non-RAHB – 78-non-RAHB** compounds. Taken from Ref.56.

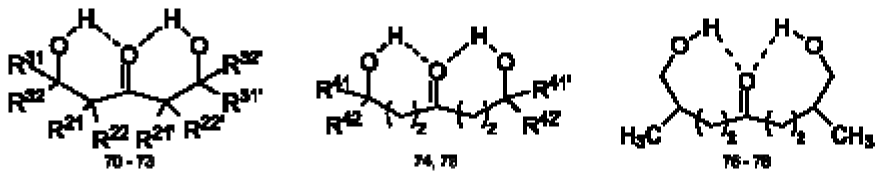

No	C2		C3'[a]		C4'[a]		conform[b]	C2'		C3'[a]		C4'[a]		conform[b]	E_{rel}[c]	$-E_{HB}$
	R^{21}	R^{22}	R^{31}	R^{32}	R^{41}	R^{42}	(config)	$R^{21'}$	$R^{22'}$	$R^{31'}$	$R^{32'}$	$R^{41'}$	$R^{42'}$	(config)		
70[d]	H	H	H	H	–	–	–G g	–	–	H	H	–	–	c c	–	2.0[d]
71a	H	H	H	H	–	–	–G g	H	H	H	H	–	–	–G g	0.0	2.3[e]
71b	H	H	H	H	–	–	–G g	H	H	H	H	–	–	G –g	0.2	2.2
72a	H	H	Me eq	H	–	–	–G g (*R*)	H	H	Me eq	H	–	–	G –g (*S*)	0.0	2.6
72b	H	H	Me eq	H	–	–	G –g (*S*)	H	H	Me eq	H	–	–	G –g (*S*)	0.3	2.8
73a	H	H	Me	Me	–	–	–G g	H	H	Me	Me	–	–	G –g	0.0	2.0
73b	H	H	Me	Me	–	–	–G g	H	H	Me	Me	–	–	–G g	0.5	2.2
74[f]	C_3H_2O		H	H	–	–	–G g	C_3H_2O		H	H	–	–	–G g	–	2.6
75[g]	$C_3H_6O_3$		H	H	–	–	–G g	$C_3H_6O_3$		H	H	–	–	–G g	–	2.6
76	H	H	H	H	H	H	G g	H	H	H	H	H	H	G g	–	3.2
77	H	H	H	H	Me	Me	G g	H	H	H	H	Me	Me	G g	–	3.3
78	H	H	H	H	Me eq	H	–G –g (*S*)	H	H	H	H	Me eq	H	–G –g (*S*)	–	2.9

Table S5. Structure of **79-non-RAHB – 88-non-RAHB** compounds. Taken from Ref.56.

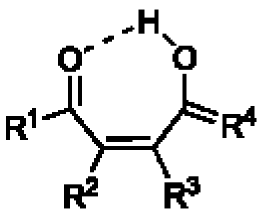

No	R^{1a}	R^2	R^3	R^4	E_{HB} rel[b]	$-E_{HB}$
79	H	H	H	=O	–	6.6
80	H	H	H	$=CH_2$	–	5.8
81a	OH trans	H	H	$=CH_2$	0.0	5.3
81b	OH cis	H	H	$=CH_2$	5.4	5.7
82a[c]	OH trans	H	H	=O	0.0	6.4
82b[c]	OH cis	H	H	=O	5.7	7.0
83a[d]	OH trans	Ph	-O-CO-CPh-		0.0	9.2
83b[d]	OH cis	Ph	-O-CO-CPh-		0.6	10.6

**84a**	OH trans	H	-O-CO-CH-		0.0	**9.2**
**84b**	OH cis	H	-O-CO-CH-		5.1	**10.1**
**85a**	OH trans	Ph	H	=CHPh	0.0	**6.3**
**85b**	OH cis	Ph	H	=CHPh	1.6	**7.1**
**86a**	NH(Me) trans	H	H	=O	0.0	**9.6**
**86b**	NH(Me) cis	H	H	=O	3.5	**9.9**
**87**	NH_2	H	H	=O	–	**9.0**
**88a**[e]	$CMe{=}CH_2$ s-cis	OMe cis	H	=O	0.0	**4.8**
**88b**[e]	$CMe{=}CH_2$ s-trans	OMe cis	H	=O	1.6	**5.4**

Table S6. Structure of **89-non-RAHB – 94-non-RAHB** compounds. Taken from Ref.56.

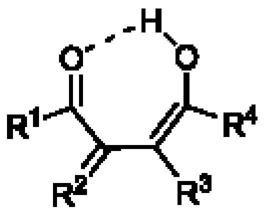

**No**	**R**[1]	**R**[2]	**R**[3]	**R**[4]	**$-E_{HB}$**
**89**	H	H H	H	H	**6.3**
**90**	H	$=CH_2$	H	H	**8.1**
**91**	Me	H H	H	Me	**6.1**
**92**	Me	$=CH_2$	H	Me	**8.2**
**93**	H	=CH(OH)	CHO	H	**13.4**
**94**	Me	=CMe(OH)	CMeO	Me	**9.6**

Table S7. Structure of **95-non-RAHB – 102-non-RAHB** compounds. Taken from Ref.56.

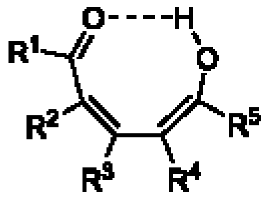

No	R^1	R^2	R^3	R^4	R^5	$-E_{HB}$
95	H	H	H	H	H	12.3
96	H	H	[a]	H	H	12.1
97	Me	Me	H	Me	Me	11.5
98	-CH_2-CH=CH-		H	-CH=CH-CH_2-		11.5
99	-CH(Me)-CH=C(Me)-		H	-CH(Me)-CH=C(Me)-		11.8
100	-CH_2-N=CH-		H	-CH=N-CH_2-		10.7
101	-CH(Me)-N=C(Me)-		H	-CH(Me)-N=C(Me)-		12.1
102a	-CH_2-CH_2-CH_2-C(OH)=		H, Me	-CO-CH_2-CH_2-CH_2-		11.3
102b	-CH_2-CH_2-CH_2-C(OH)=		H, Me	-CO-CH_2-CH_2-CH_2-		11.8

Table S8. Structure of **1-RAHB – 171-RAHB** compounds. Taken from Ref.57.

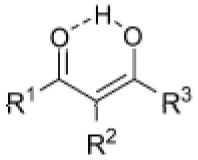

no	R^{1a}	R^2	$R^{3a,b}$	E_{rel}^{c}	$-E_{HB}$
1	H	H	H	—	14.5
2	H	H	Me	—	15.1
3	Me	H	H	—	14.6
4	H	Me	H	—	14.0
5	Me	H	Me	—	15.2
6	Me	Me	H	—	15.2
7	H	Me	Me	—	15.1
8	Me	Me	Me	—	16.2

**9a**	iPr (H c)	H	iPr (H c)	0.0	16.0
**9b**	iPr (H g)	H	iPr (H c)	0.4	15.4
**10**	iPr	Me	iPr	—	16.5
**11**	Me	H	iPr	—	15.5
**12**	iPr	H	Me	—	15.6
**13a**	tBu[d]	H	tBu[d]	0.0	15.3
**13b**	tBu[e]	H	tBu[e]	0.4	17.3
**14**	Me	H	tBu	—	15.2
**15**	tBu	H	Me	—	17.2
**16**	H	tBu	H	—	16.4
**17**	Me	iPr	Me	—	17.0
**18**	H	tBu	Me	—	16.4
**19**	Me	tBu	H	—	15.8
**20**	Ph	H	H	—	15.2
**21**	H	Ph	H	—	14.5
**22**	H	H	Ph	—	15.3
**23**	Ph	H	Ph	—	16.0
**24**	Ph	Me	Ph	—	15.8
**25**	Ph	H	Me	—	15.9
**26**	Me	H	Ph	—	15.3
**27**	Me	Ph	Me	—	15.1
**28**	Me	4-OMeC_6H_4	Me	—	15.1
**29a**	H	-N=N-Ph s-trans	H	0.0	14.0
**29b**	H	-N=N-Ph s-cis	H	1.3	15.4
**30**	Me	$-CH_2-CH_2-CH_2-CH_2-$		—	15.0
**31**	H	$-CH_2-CH_2-CH_2-CH_2-$		0.0	14.1
**32**	$-CH_2-CH_2-CH_2-CH_2-$		H	1.3	16.2
**33**	Me	$-CH_2-CH_2-CH_2-$		—	15.8
**34**	H	$-CH_2-CH_2-CH_2-$		1.3	15.3
**35**	$-CH_2-CH_2-CH_2-$		H	0.0	13.8
**36**	H	$-CH_2-CH_2-$		3.0	11.1
**37**	$-CH_2-CH_2-$		H	0.0	8.2
**38**	F	H	H	—	11.1
**39**	H	H	F	—	23.4
**40**	H	F	H	—	13.7

**41**	Me	F	Me	—	14.4
**42**	Cl	H	H	—	11.3
**43**	H	H	Cl	—	19.5
**44**	H	Cl	H	—	13.9
**45**	Me	Cl	Me	—	15.9
**46**	Br	H	H	—	11.4
**47**	H	H	Br	—	18.9
**48**	H	Br	H	—	13.7
**49**	Me	Br	Me	—	16.1
**50**	F	H	F	—	14.5
**51**	F	F	H	—	11.4
**52**	H	F	F	—	20.0
**53**	Cl	H	Cl	—	13.8
**54**	Cl	Cl	H	—	12.6
**55**	H	Cl	Cl	—	16.6
**56**	Br	H	Br	—	13.5
**57**	Br	Br	H	—	12.6
**58**	H	Br	Br	—	16.3
**59**	$CH=CH_2$ s-cis	H	Me	—	16.3
**60**	CF_3	H	Me	—	14.1
**61**	Me	H	CF_3	—	14.7
**62**	CF_3	H	tBu	—	14.3
**63**	tBu	H	CF_3	—	14.8
**64**	CF_2Cl	H	Me	—	14.4
**65**	Me	H	CF_2Cl	—	15.0
**66**	CF_3	H	Ph	—	14.3
**67**	Ph	H	CF_3	—	15.2
**68**	2-C_4H_3S	H	CF_3	—	14.6
**69**	CF_3	H	2-C_4H_3S	—	15.8
**70**	2-C_4H_3O	H	CF_3	—	14.3
**71**	CF_3	H	2-C_4H_3O	—	16.0
**72**	CF_3	H	CF_3	—	13.4
**73**	NH_2	H	H	—	13.3
**74**	H	H	NH_2	—	20.3
**75**	NH_2	H	Me	—	13.8
**76**	Me	H	NH_2	—	23.8
**77**	NH_2	NO_2	H	—	18.8

**78**	NH_2	NO_2	NH_2	—	21.2
**79a**	H	H	OH trans	0.0	26.3
**79b**	H	H	OH cis	2.4	21.0
**80**	Me	H	OMe cis	—	21.3
**81**	Ph	H	OEt cis	—	20.3
**82a**	Me	H	OCH_2Ph cis $\perp$	0.0	21.2
**82b**	Me	H	OCH_2Ph cis $\parallel$	1.6	21.0
**83a**	OH trans	H	H	0.0	11.7
**83b**	OH cis	H	H	4.8	13.0
**84a**	OH trans	H	OH trans	0.0	16.1
**84b**	OH cis	H	OH trans	4.4	18.2
**84c**	OH trans	H	OH cis	2.0	15.2
**84d**	OH cis	H	OH cis	7.1	17.0
**85a**	OMe cis	H	OH cis	0.0	14.8
**85b**	OMe cis	H	OH trans	3.3	20.9
**86a**	OH trans	H	OMe trans	0.0	17.6
**86b**	OH trans	H	OMe cis	0.4	14.1
**87a**	OMe trans	H	OH trans	0.0	15.8
**87b**	OH cis	H	OMe trans	4.9	19.9
**88a**	OMe trans	H	OMe trans	0.0	17.1
**88b**	OMe cis	H	OMe trans	4.9	23.3
**88c**	OMe trans	H	OMe cis	0.2	13.8
**88d**	OMe cis	H	OMe cis	6.2	17.5
**89a**	COOMe s-trans, trans	H	OMe cis	0.0	19.2
**89b**	COOMe s-cis, trans	H	OMe cis	1.5	19.8
**89c**	COOMe s-trans, cis	H	OMe cis	6.6	18.1
**90a**	OMe trans	H	Me	0.0	11.7
**90b**	OMe cis	H	Me	6.4	15.0
**91a**	OEt trans	H	Me	0.0	11.8
**91b**	OEt cis	H	Me	5.6	15.0
**92a**	OCH_2Ph trans $\perp$	H	Me	0.0	11.8
**92b**	OCH_2Ph trans $\parallel$	H	Me	1.0	11.8
**92c**	OCH_2Ph cis $\perp$	H	Me	5.1	14.5
**92d**	OCH_2Ph cis $\parallel$	H	Me	7.3	15.1
[illegible]	[illegible]	[illegible]	[illegible]	[illegible]	[illegible]

**93a**	OEt trans	H	Ph	0.0	11.9
**93b**	OEt cis	H	Ph	5.8	15.2
**94a**	OMe trans	H	COOMe s-cis, trans	0.0	11.9
**94b**	OMe trans	H	COOMe s-trans, trans	0.7	11.6
**94c**	OMe cis	H	COOMe s-cis, trans	6.0	15.1
**94d**	OMe cis	H	COOMe s-trans, trans	7.1	14.8
**94e**	OMe trans	H	COOMe s-cis, cis	8.2	11.9
**94f**	OMe cis	H	COOMe s-cis, cis	14.2	15.4
**95a**	OMe trans	$-CH_2-CH_2-CH_2-$		0.0	12.6
**95b**	OMe cis	$-CH_2-CH_2-CH_2-$		10.1	15.4
**96a**	OMe trans	$-CH_2-CH_2-CH_2-CH_2-$		0.0	11.7
**96b**	OMe cis	$-CH_2-CH_2-CH_2-CH_2-$		11.4	12.8
**97**[f]	OH cis	$-C_{10}H_{12}O_2-$		—	14.9
**98**[g]	OH cis	$-C_{11}H_6O-$		—	16.1
**99**[h]	OH cis	$-C_{13}H_8O-$		—	15.9
**100**[i]	OH trans	C2 = N	H	—	11.6
**101a**	OH cis	$-2-C_6H_4-CO-$		0.0	15.1
**101b**	OH trans	$-2-C_6H_4-CO$		3.2	13.8
**102**	$-O-C(CH_3)_2-O-CO-$		Me	—	15.2
**103**	H	CHO	OH s-cis	—	17.7
**104**	CH_3	COMe s-cis	OH s-cis	—	19.5
**105**	C_3H_6-CO-		OH s-cis	—	19.0
**106**	C_2H_4-CO-		OH s-cis	—	16.0
**107**	C_2H_2-CO-		OH s-cis	—	10.3
**108a**	$-C_6H_4-2-CO-$		OH cis	0.0	11.6
**108b**	$-C_6H_4-2-CO-$		OH trans	7.0	16.6
**109**	$-C_6H_4-2-CO-$		Me	—	11.8
**110a**	OH cis	CHO s-trans	H	0.0	16.9
**110b**	OH trans	CHO s-trans	H	6.8	13.2
**111a**	H	CHO s-trans	H	0.0	14.5
**111b**	H	CHO s-cis	H	1.0	16.3
**112a**	H	COMe s-trans	H	0.0	14.6
**112b**	H	COMe s-cis	H	0.7	16.4

**113a**	Me	CHO s-cis	Me	0.0	18.0
**113b**	Me	CHO s-trans	Me	0.1	17.8
**114a**	Me	COMe s-trans	Me	0.0	16.3
**114b**	Me	COMe s-cis	Me	0.1	17.1
**115a**	Me	COOMe s-cis	Me	0.0	17.5
**115b**	Me	COOMe s-trans	Me	0.1	17.4
**116a**	Me	COOEt s-cis	Me	0.0	17.3
**116b**	Me	COOEt-trans	Me	0.1	17.4
**117a**[j]	H	$C_3H_3O_2$	H	0.0	14.5
**117b**[k]	H	$C_3H_3O_2$	H	0.3	14.3
**118a**[l]	Me	$C_3H_3O_2$	Me	—	15.2
**118b**[l]	H	$C_5H_7O_2$	H	—	14.3
**119**[m]	Me	$C_5H_7O_2$	Me	—	15.1
**120**	H	OH	H	—	14.2
**121**	Me	OH	Me	—	14.8
**122**	Me	OMe	Me	—	14.7
**123**	H	NH_2	H	—	14.6
**124**	Me	NH_2	Me	—	15.1
**125**	Me	SMe	Me		15.2
**126**	Me	SPh	Me	—	15.3
**127**	H	NO_2	H	—	15.5
**128**	Me	NO_2	Me	—	16.7
**129**	H	CN	H	—	14.2
**130**	Me	CN	Me	—	15.4
**131a**	tBu[c]	CN	tBu[c]	0.0	18.6
**131b**	tBu[d]	CN	tBu[d]	2.4	16.4
**132**	Me	-CO-O-C(CH_3)=CH-		—	15.0
**133**	Me	-CO-CH(OH)-(CH_2)$_2$-		—	16.1
**134**	Me	-CO-(CH_2)$_2$-CH(OH)-		—	16.3
**135**	H	H	CHO s-cis	—	14.4
**136**	Me	H	COMe s-cis	—	15.0
**137**	Me	H	CN	—	14.1
**138a**	H	OH cis	CHO s-cis	0.0	10.6
**138b**	H	OH trans	CHO s-cis	11.1	12.4
**139a**	Me	OH cis	COMe s-cis	0.0	11.6
**139b**	Me	OH trans	COMe s-cis	14.1	13.3
**140**	Ph	OH cis	COPh s-cis	—	12.0

**141**	NO_2	NO_2	NO_2	—	12.9
**142**[n]	H	C2=N	H	—	12.6
**143**	-CH=CH-CO-		Me	—	10.4
**144**	H	-CO-CH_2-CH_2-		0.0	13.8
**145**	-CH_2-CH_2-CO-		H	1.54	15.7
**146**	Me	-CO-CH_2-CH_2-		0.0	15.6
**147**	-CO-CH_2-CH_2-		Me	1.31	16.3
**148**	H	-CH_2-CH=CH-CH_2-		0.0	13.7
**149**	-CH_2-CH=CH-CH_2-		H	1.9	17.4
**150**	CH=CH_2	H	CH=CH_2 trans	—	15.2
**151**	CH=CHPh	H	CH=CHPh	—	15.1
**152**	Me	H	CH=CH_2 s-trans	—	14.2
**153**[o]	Me	H	$C_8H_7O_2$	—	14.2
**154**[p]	$C_9H_9O_2$	H	$C_9H_9O_2$	—	15.3
**155**[q]	Me	-$C_6H_8O_2$-		—	16.6
**156**	Me	-$C_6H_8O_2$-		—	17.0
**157**	4-$NO_2C_6H_4$	H	Ph	—	16.0
**158**	4-$MeOC_6H_4$	H	Ph	—	15.8
**159**	2,4,6-triMeC_6H_2	H	Ph	—	16.0
**160**	2,4,6-triMeC_6H_2	H	4-$NO_2C_6H_4$	—	15.7
**161**	3-$NO_2C_6H_4$	H	2,4,6-triMeC_6H_2	—	16.1
**162**	2-$NO_2C_6H_4$	H	2,4,6-triMeC_6H_2	—	16.0
**163**	4-$OMeC_6H_4$	H	3-$NO_2C_6H_4$	—	15.6
**164**	3-NO_2Ph	H	4-$MeOC_6H_4$	—	16.0
**165**	2,4,6-triMeC_6H_2	H	4-$MeOC_6H_4$	—	16.0
**166**	3-BrC_6H_4	H	3-BrC_6H_4	—	15.8
**167**	2-HOC_6H_4	H	Ph	—	14.9
**168**	4-$OMeC_6H_4$	H	4-tBuC_6H_4	—	15.9
**169**	4-tBuC_6H_4	H	4-$OMeC_6H_4$	—	18.0
**170**[r]		$C_9H_6O_6$		—	18.3
**171**[s]		$C_{12}H_{12}O_6$		—	21.9

Table S9. Structure of **172-RAHB – 176-RAHB** compounds. Taken from Ref.57.

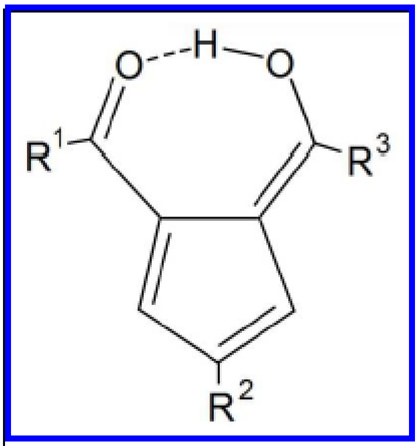

no	R1	R2	R3	−E_HB
172	H	H	H	16.7
173	Me	H	Me	17.2
174	Me	NO_2	Me	18.0
175	CH_2Cl	H	CH_2Cl	16.4
176	$CHCl_2$	H	$CHCl_2$	18.0

Table S10. Structure of **177-RAHB – 186-RAHB** compounds. Taken from ref.57.

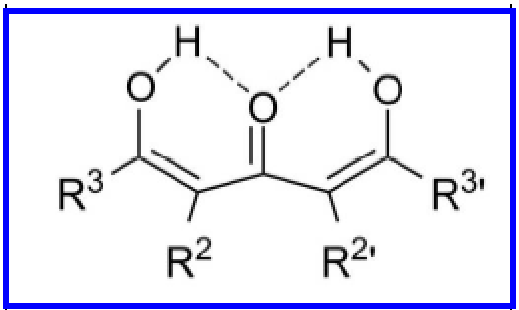

no	R2	R3	R2'	R3'	−E_HB
177	H	H	H	H	13.5
178	H	F	H	F	18.3
179	F	H	F	H	13.9
180	H	Me	H	Me	13.8
181	Me	H	Me	H	14.7
182	Me	Me	Me	Me	13.5
183	H	Ph	H	Ph	13.8
184[a]	[a]	Me	[a]	Me	15.9
185[b]	[b]	H	[b]	H	12.9
186[c]	[c]	Me	[c]	Me	11.7

Table S11. Structure of **1-arom-AHB – 18-arom-AHB** compounds. Taken from Ref.58.

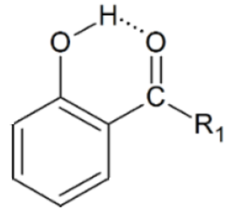

No	R_1	E_{HB}
1	H	7.9
2	Me	8.6
3	Ph	8.2
4	OH	6.8
5	OMe	7.1
6	OEt	7.2
7	OPh	7.0
8	NH_2	8.6
9	NHMe	8.1
10	NHPh	8.0
11	$N(Me)_2$	7.8
12	SH	7.0
13	SMe	7.2
14	F	5.7
15	Cl	5.7
16	Br	5.5
17	CN	6.6
18	NO_2	5.4

Table S12. Structure of **19-arom-AHB – 73-arom-AHB** compounds. Taken from ref.58.

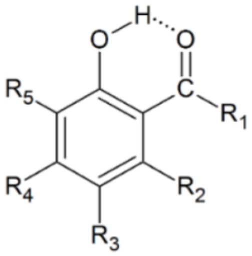

No	R_1	R_2	R_3	R_4	R_5	E_{HB}
19	H	H	Me	H	H	7.9
20	H	H	OHtrans	H	H	7.6
21	H	H	OHcis	H	H	7.9
22	H	H	NH_2	H	H	7.7
23	H	H	$N(Me)_2$	H	H	7.7
24	H	H	F	H	H	7.8
25	H	H	Cl	H	H	7.8
26	H	H	Br	H	H	7.8
27	H	H	C(O)H	H	H	7.7

**28**	H	H	H	C(O)H	H	7.9
**29**	H	H	NO_2	H	H	8.0
**30**	H	H	H	Me	H	8.1
**31**	H	H	H	OH	H	8.7
**32**	H	H	H	OMe	H	8.1
**33**	H	H	H	NH_2	H	8.7
**34**	H	H	H	$N(Me)_2$	H	8.8
**35**	H	H	H	F	H	8.3
**36**	H	H	H	Cl	H	8.1
**37**	H	H	H	Br	H	8.0
**38**	H	H	H	NO_2	H	7.9
**39**	H	H	F	H	F	7.8
**40**	H	F	H	F	H	8.9
**41**	H	OH	H	OH		9.4
**42**	H	OH trans	H	H	H	8.6
**43**	H	-O-(CO)-CH=CH-		H	H	8.9
**44**	H	OH	C(O)H	OH	C(O)H	11.3
**45**	H	H	H	NH_3^+	H	8.1
**46**	H	H	NH_3^+	H	H	8.1
**47**	H	H	H	O^-	H	10.5
**48**	H	H	O^-	H	H	7.7
**49**	Me	OHcis	H	H	H	8.4
**50**	Me	OHtrans	H	H	H	9.3
**51**	Me	OMetrans	H	H	H	9.4
**52**	Me	OH	H	Me	H	9.6
**53**	Me	H	H	OHtrans	H	9.4
**54**	Me	H	H	OMecis	H	8.8
**55**	Me	H	H	$-CH_2CH=CHCH_2-$		9.2
**56**	Ph	H	H	OMecis	H	8.5
**57**	Ph	H	Cl	Me	H	8.3
**58**	CH=CH-Ph	H	H	H	H	9.2
**59**	CH=CH-Ph	OMe	H	OMe	H	9.9
**60**	CH=C(OH)Ph	H	H	H	H	8.0
**61**	(*o*-OH)Ph	H	H	H	H	6.4
**62**	$-CH_2-CH_2-$		H	H	H	7.2
**63**	$-CH_2-CH(OH)-CH_2-$		H	H	H	8.8
**64**	-CH=CH-O-		H	H	H	9.0
**65**	$-CH_2-CH(OH)-CH_2-$		H	OH	H	9.4
**66**	$-CH_2-CH(OH)-CH_2-$		H	OH	H	9.6
**67**	$-CH_2-CH_2-C=O$		OH	H	H	9.6
**68**	$-(CH=CH)-C=CH_2$		OH	H	H	8.8
**69**	Me	OH	H	OH	H	
**70**	Me	OH	C(O)Me	OH	C(O)Me	13.1
**71**	OH	H	F	H	H	7.0
**72**	OH	H	Cl	H	H	6.9
**73**	OH	H	Br	H	H	6.9

Table S13. Structure of **74-arom-AHB – 93-arom-AHB** compounds. Taken from Ref.58.

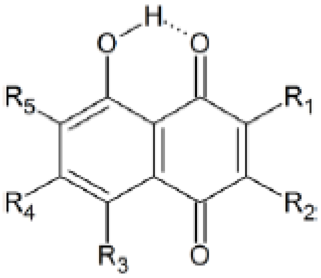

No	R_1	R_2	R_3	R_4	R_5	E_{HB}
74	H	H	H	H	H	8.3
75	H	CH_3	H	H	H	8.7
76	H	$(CH_2)_3COOH$	H	H	H	8.5
77	H	$NH(CH_2)_2COOH$	H	H	H	9.9 [a]
78a	CH_2-COOH	OH	H	H	H	8.5 [b]
78b	CH_2-COOH	OH	H	H	H	8.5 [c]
78c	CH_2-COOH	OH	H	H	H	8.8 [d]
79	$CH_2CH=C(CH_3)_2$	OH	H	H	H	9.3 [e]
80	$CH_2CH=C(CH_3)_2$	OH	H	H	H	8.7 [f]
81	H	(1,3-diOH)Ph	H	H	H	8.9
82	-O-$C(CH_3)_2$-$(CH_2)_2$-		H	H	H	8.4
83	H	H	OH	H	H	10.5
84	CH_2-CO-CH_3	CH_3	OH	H	OCH_3	11.7 [g] 11.8
85	Cl	H	OH	H	H	10.3 [g] 10.7
86	H	H	OH	Cl	H	10.3 [g] 10.7
87	Cl	Cl	OH	H	H	10.6
88	H	H	OH	Cl	Cl	10.6
89	Cl	H	OH	H	Cl	10.4 [g] 10.6
90	H	Cl	OH	H	Cl	10.9 [g] 10.2
91	Cl	Cl	OH	Cl	H	10.6 [g] 11.0
92	Cl	H	OH	Cl	Cl	10.5 [g] 10.8
93	Cl	Cl	OH	Cl	Cl	10.7

Table S14. Structure of **94-arom-AHB – 101-arom-AHB** compounds. Taken from Ref.58.

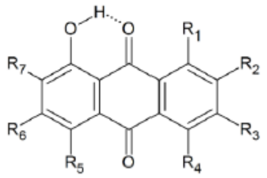

No	R_1	R_2	R_3	R_4	R_5	R_6	R_7	E_{HB}
94	H	H	H	H	H	H	H	8.8 [a]
95	H	H	H	H	H	H	OH	10.0
96	H	H	H	OH	H	H	H	9.1 [b]
97	H	H	H	H	OH	H	H	10.5 [c]
98	OH	H	H	H	H	H	H	7.6
99	OH	H	H	OH	OH	H	H	9.9
100	OH	OH	H	H	OH	H	H	9.3 [d]<br>9.5 [d]<br>11.5 [d]
101	H	OH	COOH	CH_3	OH	H	OH	11.6 [e]<br>10.9 [e]<br>5.6 [e]<br>14.9

Table S15. Structure of **102-arom-AHB – 123-arom-AHB** compounds. Taken from Ref.58.

No	Name (Customary Name)	E_{HB}
102	2-hydroxy-6-methyl [(1*R*)-2-oxo-(5*R*,6*R*)-5,6-dihydroxy-3-methyl)cyclohex-3-enoic]benzoate	5.5 [che]
103	5-hydroxy-2,2-dimethyl-8*H*-furo[3,4-g]chromen-6-one (salfredin B)	5.6
104	1,8-dihydroxyfluoren-9-one	5.8 [che]
105	1-hydroxyfluoren-9-one	6.7
106	1,8-dihydroxy-9,10-dihydroanthracen-9-one (dithranol)	7.3 [che]
107	1,8-dihydroxy-9*H*-xanthen-9-one	7.4 [che]
108	8-hydroxy-2-(1-hydroxyethylene)-3,6-dimethyl-1(2*H*)-naphthalenone	7.6 [che]
109	8-hydroxy-2-(1-hydroxyethylene)-1(2*H*)-naphthalenone	7.7 [che]
110	2,3-dihydro-2,5-dihydroxy-4*H*-benzopyran-4-one	8.2
111	3,5,7-trihydroxy-2-(4-hydroxyphenyl)-3,5,7-trihydroxy-4*H*-chromen-4-one (kemferol)	8.3 [che]
112	2-(3,4-dihydroxyphenyl)-3,5,7-trihydroxy-4*H*-chromen-4-one (quercetin)	8.4
113	(1*S**,3a*R**,9a*S**)-1,8-dihydroxy-1,2,3,3a-tetrahydrocyclopenta-[b] chromen-9(9a*H*)-one (diaporteone B)	8.7 [che]
114	1-hydroxy-9*H*-xanthen-9-one	8.7
115	9(10*H*)-1,8-dihydroxyacridinone	8.8 [che]
116	7-hydroxy-2,2-dimethyl-3,4-dihydro-2*H*-benzo[h]chromene-5,6-dione (7-hydroxylapachone)	9.3
117	5-hydroxy-4*H*-chromen-4-one	9.3
118	2-(3,4-dihydroxyphenyl)-5,7-dihydroxy-4-chromenone (luteolin)	9.8
119	7,8-dihydroxy-3-methyl-10-oxo-1*H*,10*H*-pyrano[4,3-b]chromene-9-carboxylic acid (anhydrofulvicacid)	15.4
120	5,7-dihydroxy-3-(4-hydroxyphenyl)chromen-4-one (genistein)	10.0
121	5-hydroxy-4-quinolon	11.1
122	2,3-dihydro-9,10-dihydroxy-1,4-anthracenedione (leucoquinizarin)	11.2
123	2,6-diacetyl-7,9-dihydroxy-8,9b-dimethyl-1,3(2*H*,9bh) (usinicacid)	9.9

Table S16. Characteristics of the O–H···O=C hydrogen bond for studied compounds from the non-RAHB cluster. The $-E_{HB}$ MTA energy of intramolecular hydrogen bonding (kcal/mol), the $r_{O\cdots H}$ hydrogen bond length (Å), the d_{OH} length of the O–H bond (Å), the ϑ_{HB} hydrogen bond angle (degree), the ρ_{BCP} and $\nabla^2\rho$ electron density at the bond critical point and its Laplacian, respectively (a.u.), the V_{BCP} potential energy density at the bond critical point (a.u.), the ρ_{RCP} electron density at the ring critical point, the ν_{OH} frequency of O–H stretching (cm^{-1}), the δ_{OH} ^{1}H NMR chemical shifts (ppm).

No	$-E_{HB}$	$r_{O\cdots H}$	ϕ_{HB}	d_{OH}	$r_{O\cdots O}$	ρ_{BCP}	$\nabla^2\rho$	V_{BCP}	ρ_{RCP}	ν_{OH}	$\nu_{C=O}$	δ_{OH}
**1**	**2.04**	2.2198	126.5	0.9622	2.8975	0.0152	0.0623	–0.0121	0.0129	3796	1787	1.99
**2a**	**2.47**	2.1585	129.1	0.9635	2.8658	0.0170	0.0696	–0.0145	0.0134	3783	1785	2.73
**2b**	**1.41**	2.1323	131.2	0.9640	2.8610	0.0178	0.0726	–0.0145	0.0133	3774	1786	2.44
**3**	**1.94**	2.0915	133.0	0.9650	2.8385	0.0138	0.0678	–0.0159	0.0119	3762	1785	3.07
**4**	**2.75**	2.1582	128.0	0.9628	2.8532	0.0172	0.0704	–0.0140	0.0138	3781	1765	2.55
**5a**	**3.21**	2.0917	131.1	0.9643	2.8204	0.0194	0.0793	–0.0160	0.0143	3759	1764	3.43
**5b**	**2.10**	2.0549	134.2	0.9651	2.8139	0.0208	0.0840	–0.0174	0.0141	3751	1763	3.25
**6a**	**3.26**	2.0854	131.3	0.9644	2.8164	0.0198	0.0811	–0.0163	0.0145	3760	1764	3.40
**6b**	**3.34**	2.0676	133.7	0.9709	2.8267	0.0195	0.0794	–0.0161	0.0143	3745	1757	3.40
**6c**	**3.11**	2.0805	131.5	0.9645	2.8137	0.0198	0.0811	–0.0165	0.0145	3759	1764	3.47
**6d**	**2.13**	2.0360	135.5	0.9658	2.8078	0.0215	0.0867	–0.0182	0.0143	3741	1762	2.95
**6e**	**2.19**	2.0788	133.8	0.9652	2.8339	0.0198	0.0798	–0.0182	0.0138	3749	1763	3.40
**6f**	**2.25**	1.9206	143.2	0.9667	2.7564	0.0271	0.1054	–0.0239	0.0151	3716	1761	4.64
**7**	**2.59**	1.9705	138.4	0.9687	2.7707	0.0245	0.0970	–0.0212	0.0150	3691	1760	4.26
**8a**	**2.66**	2.0018	136.8	0.9665	2.7860	0.0249	0.0740	–0.0197	0.0126	3733	1761	3.92
**8b**	**2.60**	2.0478	135.4	0.9664	2.8186	0.0210	0.0843	–0.0177	0.0140	3733	1762	3.92
**9a**	**2.81**	2.1622	126.8	0.9626	2.8454	0.0171	0.0707	–0.0140	0.0141	3785	1724	2.65
**9b**	**2.60**	2.1337	130.5	0.9643	2.8561	0.0177	0.0725	–0.0145	0.0137	3777	1783	3.18
**10a**	**3.03**	2.0639	139.8	0.9679	2.8176	0.0199	0.0776	–0.0162	0.0147	3712	1717	5.12
**10b**	**3.41**	2.0779	131.0	0.9650	2.8067	0.0200	0.0818	–0.0166	0.0148	3758	1720	4.01
**11**	**3.21**	2.1029	129.5	0.9640	2.8160	0.0191	0.0786	–0.0158	0.0146	3768	1723	3.51
**12**	**2.63**	2.0161	136.0	0.9663	2.7932	0.0190	0.0776	–0.0191	0.0124	3734	1762	3.91
**13a**	**1.93**	2.3485	120.4	0.9623	2.9547	0.0124	0.0515	–0.0100	0.0120	3807	1790	0.88
**13b**	**2.08**	2.1677	128.1	0.9629	2.8640	0.0168	0.0689	–0.0135	0.0133	3780	1786	2.25
**13c**	**2.14**	2.1425	130.3	0.9636	2.8614	0.0176	0.0712	–0.0142	0.0132	3770	1794	2.46
**14a**	**2.34**	2.1904	127.7	0.9626	2.8819	0.0167	0.0660	–0.0130	0.0134	3787	1792	2.47
**14b**	**2.54**	2.1945	126.8	0.9630	2.8768	0.0161	0.0660	–0.0130	0.0136	3783	1826	2.57
**15**	**3.69**	2.0751	131.9	0.9649	2.8127	0.0202	0.0817	–0.0168	0.0147	3739	1739	3.53
**16**	**2.54**	2.1682	127.5	0.9626	2.8579	0.0168	0.0693	–0.0137	0.0137	3782	1757	2.51
**17a**	**2.72**	2.1244	130.6	0.9639	2.8477	0.0181	0.0668	–0.0148	0.0139	3770	1789	3.29
**17b**	**2.96**	2.1280	129.7	0.9643	2.8428	0.0181	0.0743	–0.0148	0.0142	3765	1824	3.40
**18**	**4.18**	1.9987	136.0	0.9670	2.7764	0.0233	0.0933	–0.0199	0.0151	3706	1736	4.48
**19a**	**3.11**	2.0925	132.2	0.9645	2.8320	0.0193	0.0789	–0.0159	0.0142	3759	1765	3.57
**19b**	**3.40**	2.1035	129.7	0.9646	2.8190	0.0192	0.0785	–0.0158	0.0148	3763	1786	3.39
**20**	**5.79**	1.7784	149.7	0.9775	2.6684	0.0364	0.1318	–0.0341	0.0178	3492	1748	10.87
**21**	**5.53**	1.7669	151.2	0.9774	2.6651	0.0303	0.0868	–0.0351	0.0091	3503	1837	10.51
**22**	**6.40**	1.7549	149.8	0.9792	2.6477	0.0386	0.1366	–0.0366	0.0178	3459	1754	10.81
**23**	**3.45**	1.8321	149.1	0.9724	2.7125	0.0318	0.0676	–0.0292	0.0169	3604	1874	9.91
**24a**	**5.21**	1.7850	149.6	0.9763	2.6729	0.0357	0.1306	–0.0334	0.0175	3519	1840	10.67
**24b**	**5.94**	1.7551	150.2	0.9793	2.6502	0.0385	0.1367	–0.0363	0.0181	3469	1842	11.02
**25**	**2.79**	1.9830	137.2	0.9692	2.7738	0.0240	0.0950	–0.0206	0.0157	3664	1777	4.96
**26**	**3.32**	2.0768	131.8	0.9644	2.8126	0.0201	0.0815	–0.0166	0.0144	3748	1720	3.18
**27**	**2.24**	2.1335	129.0	0.9637	2.8404	0.0178	0.0737	–0.0146	0.0139	3777	1782	3.04
**28**	**5.59**	1.9557	144.3	0.9719	2.8029	0.0245	0.0937	–0.0210	0.0144	3601	1719	8.30
**29**	**6.83**	1.9328	144.9	0.9736	2.7863	0.0259	0.0975	–0.0224	0.0148	3570	1708	8.73
**30a**	**2.40**	2.1232	129.2	0.9640	2.8330	0.0182	0.0753	–0.0150	0.0140	3773	1778	2.85
**30b**	**2.09**	2.0188	132.0	0.9645	2.7589	0.0224	0.0929	–0.0192	0.0152	3763	1775	3.47
**31a**	**2.63**	2.2077	126.8	0.9637	2.8901	0.0155	0.0635	–0.0124	0.0130	3792	1789	2.38
**31b**	**1.80**	2.1571	127.4	0.9644	2.8477	0.0171	0.0708	–0.0139	0.0137	3774	1788	2.27

**32a**	**3.56**	1.9889	140.6	0.9679	2.8047	0.0233	0.0904	–0.0195	0.0153	3700	1703	4.13
**32b**	**3.15**	2.1594	127.6	0.9629	2.8508	0.0172	0.0706	–0.0140	0.0140	3779	1758	2.41
**33a**	**5.00**	1.8001	148.6	0.9762	2.6820	0.0347	0.1273	–0.0324	0.0177	3526	1763	10.55
**33b**	**5.31**	1.8041	146.4	0.9781	2.6743	0.0347	0.1260	–0.0322	0.0183	3479	1784	10.88
**33c**	**5.93**	1.7469	149.2	0.9804	2.6373	0.0394	0.1385	–0.0376	0.0184	3423	1782	11.59
**33d**	**4.86**	1.8321	146.8	0.9752	2.7016	0.0323	0.1207	–0.0296	0.0174	3547	1763	10.16
**33e**	**4.46**	1.7897	147.0	0.9761	2.6619	0.0356	0.1310	–0.0334	0.0181	3503	1749	10.84
**34**	**12.48**	1.6100	154.4	1.0002	2.5489	0.0553	0.1625	–0.0563	0.0202	3078	1632	14.22
**35**	**11.15**	1.6274	153.6	0.9962	2.5582	0.0528	0.1608	–0.0533	0.0199	3129	1646	14.10
**36**	**9.35**	1.6192	151.6	0.9926	2.5367	0.0536	0.1662	–0.0549	0.0203	3089	1718	14.19
**37a**	**9.28**	1.7338	151.3	0.9869	2.6425	0.0407	0.1372	–0.0387	0.0184	3318	1699	12.08
**37b**	**6.92**	1.7455	152.0	0.9802	2.6513	0.0394	0.1376	–0.0377	0.0180	3421	1720	11.21
**38**	**2.41**	2.0581	135.5	0.9673	2.8300	0.0204	0.0819	–0.0170	0.0142	3715	1780	4.17
**39**	**3.30**	2.0816	131.4	0.9646	2.8138	0.0198	0.0811	–0.0165	0.0144	3748	1756	3.55
**40**	**2.82**	2.2132	125.9	0.9629	2.8855	0.0155	0.0634	–0.0124	0.0130	3789	1715	2.11
**41a**	**2.76**	2.2212	125.6	0.9628	2.8901	0.0152	0.0625	–0.0122	0.0129	3790	1708	2.09
**41b**	**3.00**	2.2522	123.7	0.9627	2.8994	0.0145	0.0599	–0.0117	0.0131	3799	1715	1.88
**42**	**6.43**	2.0065	148.2	0.9765	2.8824	0.0216	0.0821	–0.0180	0.0142	3565	1747	9.17
**43**	**13.71**	1.5474	149.2	1.0003	2.4574	0.0651	0.1844	–0.0712	0.0223	2880	1625	15.82
**44a**	**2.66**	2.1957	129.9	0.9626	2.9082	0.0151	0.0613	–0.0119	0.0094	3787	1791	0.50
**44b**	**3.32**	1.8980	157.7	0.9654	2.8151	0.0262	0.1006	–0.0224	0.0086	3710	1789	3.81
**45a**	**3.11**	1.8837	160.4	0.9661	2.8125	0.0291	0.1007	–0.0233	0.0069	3706	1789	4.30
**45b**	**3.27**	1.8965	156.2	0.9665	2.8126	0.0265	0.1012	–0.0227	0.0086	3696	1788	3.90
**46**	**3.14**	1.8782	159.3	0.9672	2.8038	0.0275	0.1041	–0.0239	0.0087	3693	1788	4.34
**47a**	**4.59**	1.8983	157.2	0.9707	2.8182	0.0264	0.0976	–0.0228	0.0106	3613	1768	4.06
**47b**	**4.12**	1.9983	139.5	0.9644	2.8023	0.0224	0.0891	–0.0187	0.0103	3735	1742	3.48
**48**	**3.95**	1.8639	159.4	0.9665	2.7895	0.0284	0.1073	–0.0247	0.0090	3688	1768	4.21
**49a**	**3.75**	1.8491	162.6	0.9673	2.7872	0.0293	0.1091	–0.0259	0.0093	3681	1767	4.76
**49b**	**3.90**	1.8689	157.2	0.9674	2.7860	0.0282	0.1074	–0.0257	0.0091	3673	1766	4.34
**50**	**3.78**	1.8506	159.3	0.9681	2.7772	0.0300	0.1099	–0.0259	0.0089	3671	1766	4.78
**51**	**4.73**	1.9940	137.8	0.9651	2.7858	0.0228	0.0915	–0.0192	0.0132	3734	1722	4.04
**52**	**5.90**	1.8155	157.0	0.9748	2.7394	0.0319	0.1153	–0.0285	0.0107	3525	1756	9.82
**53a**	**3.34**	1.8933	156.9	0.9650	2.8066	0.0263	0.1026	–0.0102	0.0087	3720	1789	3.89
**53b**	**3.39**	1.8982	155.1	0.9652	2.8033	0.0260	0.1020	–0.0077	0.0090	3717	1825	4.00
**53c**	**2.73**	2.3094	124.0	0.9625	2.9570	0.0132	0.0527	–0.0102	0.0108	3796	1830	5.59
**54a**	**3.89**	1.8696	158.1	0.9661	2.7894	0.0279	0.1075	–0.0243	0.0087	3703	1766	4.20
**54b**	**3.74**	1.8895	154.7	0.9655	2.7930	0.0265	0.1038	–0.0229	0.0095	3713	1788	4.04
**55**	**4.12**	1.8553	160.2	0.9669	2.7841	0.0290	0.1091	–0.0254	0.0090	3682	1760	4.34
**56a**	**5.13**	1.8419	156.5	0.9728	2.7616	0.0298	0.1106	–0.0263	0.0104	3571	1775	9.49
**56b**	**5.38**	1.8413	155.7	0.9734	2.7577	0.0298	0.1105	–0.0264	0.0106	3560	1830	9.58
**57a**	**5.45**	1.8443	157.9	0.9735	2.7704	0.0296	0.1091	–0.0260	0.0105	3557	1744	9.63
**57b**	**5.82**	1.7668	169.0	0.9774	2.7325	0.0356	0.1240	–0.0322	0.0108	3474	1771	10.61
**58**	**5.91**	1.7329	160.2	0.9809	2.6764	0.0391	0.1334	–0.0365	0.0114	3351	1761	11.57
**59**	**6.31**	1.9792	121.2	0.9793	2.7021	0.0367	0.1279	–0.0336	0.0103	3438	1760	10.18
**60**	**3.08**	2.1839	159.1	0.9638	3.1032	0.0130	0.0408	–0.0099	0.0060	3769	1787	1.63
**61**	**3.75**	2.0861	166.1	0.9648	3.0314	0.0146	0.0484	–0.0125	0.0131	3722	1767	3.14
**62**	**4.14**	1.9012	167.7	0.9681	2.8545	0.0251	0.0947	–0.0210	0.0052	3674	1768	3.97
**63a**	**5.88**	1.8802	173.7	0.9726	2.8489	0.0254	0.0963	–0.0215	0.0083	3585	1775	9.72
**63b**	**6.05**	1.8758	173.0	0.9730	2.8440	0.0256	0.0972	–0.0217	0.0085	3583	1808	9.89
**64a**	**6.20**	1.8392	173.6	0.9750	2.8103	0.0280	0.1045	–0.0243	0.0086	3542	1769	10.10
**64b**	**6.50**	1.8565	173.8	0.9750	2.8278	0.0271	0.1082	–0.0231	0.0072	3556	1802	9.88
**64c**	**6.40**	1.8066	172.6	0.9749	2.7762	0.0304	0.1124	–0.0267	0.0074	3534	1803	10.24
**65**	**4.44**	1.8896	154.6	0.9718	2.7988	0.0261	0.0997	–0.0225	0.0091	3561	1789	8.96
**66**	**4.03**	1.9376	150.5	0.9710	2.8232	0.0240	0.0924	–0.0204	0.0084	3616	1784	8.36
**67a**	**6.01**	1.8172	177.2	0.9749	2.7913	0.0301	0.1091	–0.0264	0.0067	3543	1765	9.95
**67b**	**6.23**	1.8070	177.4	0.9756	2.7819	0.0308	0.1112	–0.0272	0.0087	3530	1816	10.22

**68a**	**3.71**	1.8437	147.4	0.9708	2.7123	0.0292	0.1131	–0.0263	0.0099	3541	1768	10.57
**68b**	**4.26**	1.7948	151.1	0.9731	2.6882	0.0324	0.1220	–0.0297	0.0101	3509	1808	10.97
**69a**	**5.31**	1.9523	169.5	0.9704	2.9118	0.0202	0.0816	–0.0165	0.0065	3632	1771	8.69
**69b**	**5.30**	1.9578	170.4	0.9703	2.9190	0.0197	0.0802	–0.0161	0.0064	3637	1804	8.74
**70**	**2.03**	2.1947	126.7	0.9622	2.8747	0.0162	0.0657	–0.0129	0.0134	3788	1755	2.61
**71a**	**2.32**	2.1796	126.8	0.9623	2.8610	0.0165	0.0680	–0.0134	0.0135	3790	1744	2.43
**71b**	**2.16**	2.1967	126.2	0.9620	2.8715	0.0160	0.0659	–0.0130	0.0134	3797	1746	2.26
**72a**	**2.61**	2.1321	129.0	0.9634	2.8385	0.0180	0.0741	–0.0148	0.0139	3781	1743	3.08
**72b**	**2.79**	2.1108	130.0	0.9637	2.8280	0.0186	0.0768	–0.0154	0.0139	3775	1742	3.20
**73a**	**2.04**	2.0443	133.9	0.9651	2.8009	0.0205	0.0865	–0.0180	0.0134	3765	1740	3.77
**73b**	**2.17**	2.0341	134.8	0.9654	2.7991	0.0216	0.0875	–0.0183	0.0142	3753	1739	3.72
**74**	**2.57**	2.2240	125.1	0.9626	2.8867	0.0152	0.0626	–0.0122	0.0129	3797	1706	2.13
**75**	**2.60**	2.2550	122.8	0.9624	2.8922	0.0145	0.0602	–0.0117	0.0131	3804	1682	1.76
**76**	**3.22**	2.3189	122.8	0.9622	2.9531	0.0130	0.0521	–0.0101	0.0109	3800	1754	0.87
**77**	**3.33**	2.2613	125.4	0.9640	2.9276	0.0143	0.0579	–0.0112	0.0116	3788	1749	0.28
**78**	**2.87**	2.2123	158.7	0.9636	3.1296	0.0125	0.0492	–0.0094	0.0138	3747	1749	2.57
**79**	**6.56**	1.6509	172.3	0.9826	2.6280	0.0473	0.1541	–0.0459	0.0097	3325	1739	12.75
**80**	**5.77**	1.6682	170.6	0.9800	2.6399	0.0456	0.1491	–0.0437	0.0094	3334	1727	10.66
**81a**	**5.25**	1.6764	170.1	0.9769	2.6440	0.0440	0.1492	–0.0422	0.0094	3422	1741	10.40
**81b**	**5.66**	1.6615	169.9	0.9788	2.6307	0.0458	0.1526	–0.0442	0.0095	3383	1768	10.74
**82a**	**6.41**	1.6538	171.9	0.9809	2.6286	0.0464	0.1547	–0.0450	0.0097	3372	1790	12.39
**82b**	**7.01**	1.6357	171.8	0.9834	2.6127	0.0487	0.1588	–0.0477	0.0099	3323	1797	12.77
**83a**	**9.22**	1.5645	168.1	0.9934	2.5447	0.0587	0.1737	–0.0606	0.0099	3085	1709	13.78
**83b**	**10.62**	1.5470	168.9	0.9986	2.5341	0.0617	0.1747	–0.0644	0.0101	2990	1733	14.45
**84a**	**9.20**	1.6053	167.7	0.9907	2.5819	0.0528	0.1626	–0.0525	0.0096	3150	1732	12.60
**84b**	**10.07**	1.5865	167.9	0.9940	2.5669	0.0555	0.1661	–0.0559	0.0098	3083	1753	13.14
**85a**	**6.32**	1.6212	169.4	0.9821	2.5928	0.0506	0.1631	–0.0504	0.0098	3301	1715	12.20
**85b**	**7.09**	1.6064	169.8	0.9853	2.5820	0.0527	0.1655	–0.0529	0.0100	3242	1740	12.66
**86a**	**9.61**	1.5896	173.5	0.9920	2.5776	0.0553	0.1672	–0.0558	0.0101	3158	1694	13.93
**86b**	**9.88**	1.5787	173.2	0.9931	2.5675	0.0568	0.1716	–0.0578	0.0102	3131	1696	14.11
**87**	**9.02**	1.5981	173.1	0.9900	2.5836	0.0540	0.1661	–0.0541	0.0101	3196	1712	13.60
**88a**	**4.84**	1.7767	155.3	0.9732	2.6915	0.0344	0.1263	–0.0317	0.0103	3492	1692	10.31
**88b**	**5.36**	1.7350	157.6	0.9749	2.6623	0.0379	0.1359	–0.0357	0.0101	3468	1717	10.81
**89**	**6.32**	1.7243	164.6	0.9755	2.6773	0.0394	0.1377	–0.0366	0.0087	3507	1780	9.17
**90**	**8.13**	1.6842	165.1	0.9807	2.6439	0.0437	0.1465	–0.0415	0.0090	3376	1732	10.81
**91**	**6.05**	1.8639	155.1	0.9738	2.7775	0.0287	0.1044	–0.0251	0.0109	3542	1744	8.34
**92**	**8.19**	1.6327	166.4	0.9821	2.5976	0.0495	0.1617	–0.0488	0.0097	3338	1706	11.86
**93**	**13.36**	1.4887	168.8	1.0149	2.4921	0.0734	0.1748	–0.0808	0.0101	2647	1696	16.49
**94**	**9.58**	1.6390	161.6	0.9906	2.5978	0.0499	0.1537	–0.0489	0.0127	3068	1647	12.55
**95**	**12.32**	1.5511	171.2	0.9980	2.5418	0.0604	0.1710	–0.0619	0.0063	2727	1696	14.03
**96**	**12.07**	1.5896	163.9	0.9883	2.5538	0.0548	0.1696	–0.0555	0.0082	2988	1703	8.29
**97**	**11.46**	1.5709	165.8	0.9900	2.5422	0.0579	0.1735	–0.0595	0.0091	2805	1661	9.57
**98**	**11.51**	1.5571	173.9	0.9972	2.5509	0.0593	0.1688	–0.0604	0.0058	2833	1700	14.22
**99**	**11.83**	1.5518	173.5	0.9987	2.5466	0.0602	0.1692	–0.0616	0.0058	2782	1693	13.63
**100**	**10.68**	1.5829	179.3	0.9971	2.5799	0.0555	0.1614	–0.0550	0.0045	2830	1722	15.84
**101**	**12.08**	1.5619	174.5	0.9992	2.5584	0.0586	0.1655	–0.0592	0.0054	2770	1712	15.38
**102a**	**11.29**	1.6537	167.4	0.9960	2.6346	0.0464	0.1421	–0.0439	0.0140	3070	1647	12.93
**102b**	**11.76**	1.6004	168.0	0.9993	2.5861	0.0533	0.1558	–0.0525	0.0106	2959	1647	13.40

Table S17. Characteristics of the O–H···O=C hydrogen bond for studied compounds from the RAHB cluster. The $-E_{HB}$ MTA energy of intramolecular hydrogen bonding (kcal/mol), the $r_{O\cdots H}$ hydrogen bond length (Å), the d_{OH} length of the O–H bond (Å), the ϑ_{HB} hydrogen bond angle (degree), the ρ_{BCP} and $\nabla^2\rho$ electron density at the bond critical point and its Laplacian, respectively (a.u.), the V_{BCP} potential energy density at the bond critical point (a.u.), the ρ_{RCP} electron density at the ring critical point, the ν_{OH} frequency of O–H stretching (cm^{-1}), the δ_{OH} ^{1}H NMR chemical shifts (ppm).

N	$-E_{HB}$	$r_{O\cdots H}$	ϕ_{HB}	d_{OH}	$r_{O\cdots O}$	ρ_{BCP}	$\nabla^2\rho$	V_{BCP}	ρ_{RCP}	ν_{OH}	$\nu_{C=O}$	δ_{OH}
**1**	**14.51**	1.6880	147.9	0.992	2.583	0.0458	0.1496	−0.0449	0.0196	3197	1694	14.05
**2**	**15.10**	1.6507	149.9	0.996	2.562	0.0501	0.1559	−0.0501	0.0203	3192	1687	14.73
**3**	**14.57**	1.6684	148.3	0.994	2.567	0.0481	0.1539	−0.0476	0.0199	3255	1689	14.04
**4**	**14.03**	1.6854	147.6	0.990	2.577	0.0460	0.1522	−0.0455	0.0196	3218	1702	13.63
**5**	**15.16**	1.6351	150.1	0.997	2.549	0.0522	0.1593	−0.0526	0.0204	3067	1676	14.88
**6**	**15.17**	1.6394	148.5	0.995	2.541	0.0516	0.1621	−0.0523	0.0203	3164	1691	14.10
**7**	**15.09**	1.6139	150.6	0.998	2.531	0.0549	0.1654	−0.0564	0.0209	3026	1680	15.32
**8**	**16.23**	1.5745	151.4	1.003	2.502	0.0607	0.1732	−0.0640	0.0213	2928	1665	15.74
**9a**	**15.95**	1.6167	150.8	1.000	2.537	0.0546	0.1623	−0.0556	0.0208	3015	1663	15.08
**9b**	**15.44**	1.6193	150.8	0.999	2.538	0.0543	0.1625	−0.0553	0.0208	3028	1661	15.09
**10**	**16.51**	1.5474	152.1	1.006	2.481	0.0649	0.1780	−0.0700	0.0218	2877	1647	16.53
**11**	**15.46**	1.6293	150.4	0.998	2.546	0.0529	0.1600	−0.0535	0.0205	3051	1669	15.02
**12**	**15.63**	1.6225	150.4	0.999	2.540	0.0539	0.1616	−0.0547	0.0207	3040	1671	14.98
**13a**	**15.32**	1.6073	151.2	1.000	2.530	0.0558	0.1654	−0.0574	0.0210	3016	1653	15.39
**13b**	**17.28**	1.5700	152.1	1.006	2.504	0.0613	0.1707	−0.0645	0.0217	2908	1641	16.32
**14**	**15.24**	1.6156	151.2	0.999	2.537	0.0547	0.1633	−0.0558	0.0208	3021	1663	15.26
**15**	**17.2**	1.5885	151.1	1.004	2.515	0.0586	0.1675	−0.0608	0.0213	2962	1661	15.84
**16**	**16.38**	1.6036	149.4	1.001	2.542	0.0532	0.1683	−0.0584	0.0211	3000	1647	15.40
**17**	**17.03**	1.5249	152.0	1.009	2.462	0.0689	0.1813	−0.0758	0.0221	2769	1636	16.84
**18**	**16.38**	1.5223	152.5	1.009	2.462	0.0693	0.1823	−0.0766	0.0225	2765	1647	17.26
**19**	**15.82**	1.5589	149.2	1.001	2.473	0.0631	0.1810	−0.0679	0.0217	2974	1661	15.61
**20**	**15.22**	1.6451	148.7	0.996	2.549	0.0508	0.1590	−0.0511	0.0203	3104	1667	15.56
**21**	**14.52**	1.6639	148.3	0.994	2.563	0.0485	0.1556	−0.0483	0.0199	3129	1695	14.47
**22**	**15.29**	1.6253	151.1	0.999	2.546	0.0534	0.1601	−0.0541	0.0208	3016	1668	15.92
**23**	**15.98**	1.5920	151.5	1.003	2.520	0.0579	0.1669	−0.0600	0.0212	2923	1650	16.49
**24**	**15.77**	1.55469	151.6	1.005	2.486	0.0636	0.1775	−0.0683	0.0216	2847	1624	14.57
**25**	**15.91**	1.6110	150.6	1.0005	2.531	0.0553	0.1642	−0.0566	0.0208	3018	1660	15.76
**26**	**15.26**	1.6138	151.2	1.0000	2.536	0.0550	0.1627	−0.0561	0.0208	2909	1657	16.31
**27**	**15.05**	1.5971	150.9	1.000	2.518	0.0573	0.1686	−0.0595	0.0208	2953	1658	15.92
**28**	**15.12**	1.59641	150.9	1.000	2.518	0.0574	0.1687	−0.0596	0.0208	3119	1686	14.39
**29a**	**13.95**	1.6781	147.7	0.994	2.573	0.0469	0.1515	−0.0463	0.0194	3135	1698	14.25
**29b**	**15.35**	1.6510	149.0	0.998	2.559	0.0501	0.1553	−0.0500	0.0201	3063	1692	14.53
**30**	**14.96**	1.5970	150.5	0.999	2.515	0.0573	0.1698	−0.0596	0.0210	3012	1644	15.28
**31**	**14.08**	1.6496	149.7	0.994	2.557	0.0502	0.1582	−0.0505	0.0203	3119	1686	14.39
**32**	**16.22**	1.6206	148.9	0.998	2.529	0.0541	0.1646	−0.0553	0.0207	3151	1681	14.57
**33**	**15.76**	1.7191	146.9	0.992	2.607	0.0428	0.1404	−0.0410	0.0191	3193	1675	13.24
**34**	**15.26**	1.7504	146.3	0.990	2.631	0.0397	0.1336	−0.0374	0.0186	3242	1686	12.98
**35**	**11.9**	1.7475	148.2	0.988	2.639	0.0396	0.1354	−0.0376	0.0187	3290	1721	12.72
**36**	**11.1**	2.1289	135.9	0.976	2.910	0.0173	0.0666	−0.0137	0.0131	3583	1707	9.39
**37**	**8.22**	2.0767	144.9	0.976	2.929	0.0186	0.0731	−0.0153	0.0141	3607	1774	8.62
**38**	**11.08**	1.8141	143.7	0.980	2.667	0.0339	0.1255	−0.0313	0.0181	3465	1779	10.75
**39**	**23.39**	1.5048	152.8	1.034	2.470	0.0730	0.1610	−0.0796	0.0235	2501	1698	17.11
**40**	**13.73**	1.7626	145.8	0.985	2.635	0.0384	0.1346	−0.0363	0.0179	3328	1721	11.51
**41**	**14.35**	1.6849	148.6	0.990	2.583	0.0462	0.1504	−0.0455	0.0191	3183	1695	12.66
**42**	**11.28**	1.8270	141.1	0.983	2.665	0.0330	0.1230	−0.0303	0.0178	3464	1744	10.77
**43**	**19.52**	1.5448	152.4	1.021	2.495	0.0656	0.1658	−0.0695	0.0215	2646	1668	16.42
**44**	**13.89**	1.7202	144.8	0.993	2.596	0.0424	0.1436	−0.0411	0.0189	3262	1699	12.94

**45**	**15.9**	1.6073	150.3	0.999	2.525	0.0559	0.1663	–0.0575	0.0207	3002	1655	14.76
**46**	**11.41**	1.7941	143.5	0.980	2.647	0.0354	0.1299	–0.0331	0.0183	3489	1750	10.43
**47**	**18.94**	1.5551	152.2	1.019	2.502	0.0638	0.1656	–0.0672	0.0223	2609	1663	16.64
**48**	**13.66**	1.7093	146.8	0.988	2.593	0.0434	0.1464	–0.0423	0.0192	3259	1691	13.20
**49**	**16.08**	1.5847	150.8	1.002	2.508	0.0591	0.1707	–0.0618	0.0212	2977	1645	15.24
**50**	**14.53**	1.7318	145.8	0.991	2.611	0.0411	0.1390	–0.0391	0.0200	3275	1775	11.78
**51**	**11.39**	1.8629	141.9	0.976	2.699	0.0303	0.1160	–0.0273	0.0169	3524	1789	8.68
**52**	**20.01**	1.5905	150.6	1.014	2.522	0.0584	0.1597	–0.0597	0.0213	2789	1733	14.45
**53**	**13.81**	1.6989	147.2	0.992	2.589	0.0443	0.1464	–0.0431	0.0201	3257	1724	12.13
**54**	**12.63**	1.7411	147.7	0.982	2.599	0.0401	0.1435	–0.0387	0.0191	3420	1715	10.87
**55**	**16.55**	1.5864	151.1	1.008	2.517	0.0588	0.1656	–0.0609	0.0215	2784	1671	15.47
**56**	**13.54**	1.7002	147.1	0.992	2.589	0.0441	0.1464	–0.0429	0.0201	3281	1728	11.76
**57**	**12.61**	1.726	143.8	0.983	2.585	0.0415	0.1473	–0.0405	0.0194	3426	1709	11.03
**58**	**16.29**	1.5726	151.4	1.010	2.507	0.0608	0.1683	–0.0637	0.0218	2740	1659	15.77
**59**	**16.29**	1.6110	150.9	1.001	2.533	0.0553	0.1632	–0.0565	0.0209	2984	1615	15.72
**60**	**14.12**	1.6892	148.3	0.990	2.584	0.0454	0.1509	–0.0448	0.0198	3180	1683	14.30
**61**	**14.66**	1.6502	148.3	0.996	2.552	0.0502	0.1565	–0.0502	0.0202	3102	1701	14.47
**62**	**14.3**	1.6686	149.4	0.992	2.572	0.0477	0.1554	–0.0476	0.0203	3196	1685	14.22
**63**	**14.8**	1.6395	148.4	0.997	2.543	0.0515	0.1591	–0.0519	0.0205	3094	1693	13.08
**64**	**14.36**	1.6817	148.4	0.991	2.578	0.0462	0.1523	–0.0458	0.0199	3223	1687	13.84
**65**	**14.99**	1.6294	149.3	0.999	2.540	0.0528	0.1606	–0.0534	0.0206	3044	1696	14.83
**66**	**14.31**	1.6641	149.5	0.993	2.597	0.0482	0.1554	–0.0482	0.0203	3087	1666	15.15
**67**	**15.22**	1.6271	148.7	0.999	2.534	0.0530	0.1614	–0.0538	0.0207	3037	1681	15.38
**68**	**14.61**	1.6410	148.4	0.998	2.545	0.0511	0.1586	–0.0514	0.0204	3082	1618	14.70
**69**	**15.76**	1.6173	150.4	1.000	2.536	0.0541	0.1625	–0.0552	0.0211	3063	1652	15.07
**70**	**14.32**	1.6473	148.5	0.997	2.551	0.0505	0.1573	–0.0506	0.0203	3087	1685	14.74
**71**	**16.03**	1.6249	150.4	1.000	2.542	0.0531	0.1614	–0.0541	0.0210	3097	1655	14.96
**72**	**13.41**	1.7177	145.8	0.998	2.595	0.0423	0.1454	–0.0412	0.0193	3282	1722	13.26
**73**	**13.26**	1.6848	148.2	0.992	2.581	0.0461	0.1499	–0.0451	0.0196	3232	1696	13.12
**74**	**20.33**	1.5144	151.7	1.029	2.469	0.0737	0.1628	–0.0807	0.0231	2571	1642	16.99
**75**	**13.75**	1.6539	149.9	0.995	2.564	0.0497	0.1560	–0.0494	0.0200	3145	1687	13.88
**76**	**23.75**	1.4493	155.6	1.046	2.441	0.0848	0.1513	–0.0979	0.0238	2366	1614	18.24
**77**	**18.79**	1.5514	150.6	1.013	2.484	0.0642	0.1708	–0.0681	0.0218	2810	1691	15.39
**78**	**21.18**	1.3953	156.9	1.052	2.399	0.0972	0.1380	–0.1213	0.0234	2251	1731	18.14
**79a**	**26.32**	1.4266	155.2	1.059	2.429	0.0902	0.1360	–0.1066	0.0244	2194	1642	18.89
**79b**	**21.01**	1.5363	153.2	1.022	2.491	0.0672	0.1649	–0.0716	0.0227	2674	1683	16.39
**80**	**21.34**	1.5128	152.3	1.031	2.473	0.0715	0.1644	–0.0776	0.0227	2609	1635	16.83
**81**	**20.28**	1.5146	153.9	1.024	2.475	0.0710	0.1688	–0.0774	0.0229	2650	1633	17.01
**82a**	**21.15**	1.5047	154.2	1.028	2.471	0.0730	0.1665	–0.0801	0.0231	2567	1631	16.99
**82b**	**21.04**	1.5079	154.1	1.026	2.472	0.0724	0.1670	–0.0792	0.0230	2611	1632	16.82
**83a**	**11.71**	1.7576	145.6	0.984	2.629	0.0386	0.1365	–0.0366	0.0186	3374	1716	11.73
**83b**	**12.97**	1.7211	146.4	0.988	2.603	0.0421	0.1434	–0.0406	0.0193	3307	1742	12.43
**84a**	**16.1**	1.6225	149.8	1.003	2.540	0.0535	0.1574	–0.0537	0.0211	3012	1719	13.76
**84b**	**18.2**	1.5772	151.1	1.012	2.512	0.0603	0.1621	–0.0620	0.0220	2856	1731	14.81
**84c**	**15.15**	1.6549	149.6	0.997	2.565	0.0494	0.1536	–0.0489	0.0207	3137	1709	13.03
**84d**	**17.01**	1.6114	150.8	1.004	2.536	0.0553	0.1600	–0.0560	0.0215	3007	1728	13.96
**85a**	**14.8**	1.6568	149.8	0.997	2.568	0.0493	0.1533	–0.0487	0.0206	3136	1693	14.59
**85b**	**20.86**	1.5219	152.5	1.024	2.476	0.0696	0.1648	–0.0746	0.0229	2667	1658	15.91
**86a**	**17.64**	1.5970	151.4	1.007	2.528	0.0570	0.1610	–0.0581	0.0216	2948	1685	14.23
**86b**	**14.07**	1.6604	149.8	0.995	2.569	0.0487	0.1538	–0.0482	0.0206	3164	1700	12.89
**87a**	**15.75**	1.6237	150.0	1.003	2.542	0.0535	0.1213	–0.0536	0.0211	3017	1652	13.78
**87b**	**19.94**	1.5514	152.7	1.018	2.500	0.0644	0.1641	–0.0676	0.0225	2771	1703	15.37
**88a**	**17.13**	1.6003	151.6	1.007	2.531	0.0567	0.1606	–0.0576	0.0215	2956	1678	14.27
**88b**	**23.26**	1.4864	154.3	1.035	2.460	0.0765	0.1617	–0.0844	0.0237	2570	1657	16.58

**88c**	**13.76**	1.6607	150.1	0.995	2.571	0.0488	0.1538	–0.0483	0.0206	3165	1682	12.98
**88d**	**17.53**	1.5774	151.9	1.008	2.512	0.0603	0.1663	–0.0626	0.0221	2932	1683	14.62
**89a**	**19.23**	1.5541	153.0	1.016	2.502	0.0639	0.1685	–0.0677	0.0226	2853	1685	15.57
**89b**	**19.8**	1.5392	153.2	1.019	2.491	0.0665	0.1691	–0.0712	0.0228	2804	1659	15.91
**89c**	**18.13**	1.5772	152.2	1.010	2.515	0.0600	0.1669	–0.0626	0.0220	2942	1650	14.91
**90a**	**11.72**	1.7230	147.8	0.986	2.611	0.0419	0.1425	–0.0405	0.0191	3319	1690	12.44
**90b**	**14.96**	1.6471	149.2	0.995	2.553	0.0506	0.1578	–0.0507	0.0206	3158	1693	13.69
**91a**	**11.83**	1.7188	148.0	0.986	2.608	0.0426	0.1437	–0.0410	0.0192	3310	1687	12.43
**91b**	**15.03**	1.6436	149.4	0.995	2.551	0.0510	0.1583	–0.0512	0.0206	3142	1689	13.74
**92a**	**11.82**	1.7205	147.9	0.986	2.609	0.0421	0.1436	–0.0407	0.0191	3318	1684	12.47
**92b**	**11.75**	1.7224	147.9	0.986	2.610	0.0420	0.1431	–0.0406	0.0191	3318	1686	12.40
**92c**	**14.54**	1.6551	149.0	0.993	2.558	0.0496	0.1566	–0.0408	0.0204	3169	1690	13.55
**92d**	**15.12**	1.6429	149.4	0.995	2.550	0.0511	0.1584	–0.0513	0.0206	3144	1688	13.82
**93a**	**11.88**	1.6879	149.0	0.989	2.595	0.0445	0.1477	–0.0435	0.0196	3263	1675	13.05
**93b**	**15.23**	1.6242	150.3	0.998	2.540	0.0535	0.1614	–0.0542	0.021	3076	1677	14.55
**94a**	**11.88**	1.7350	147.1	0.986	2.618	0.0407	0.1406	–0.0391	0.0191	3350	1695	11.90
**94b**	**11.56**	1.7404	147.0	0.985	2.622	0.0402	0.1402	–0.0386	0.0190	3364	1697	11.88
**94c**	**15.12**	1.6599	148.5	0.995	2.561	0.0490	0.1551	–0.0488	0.0206	3186	1699	13.30
**94d**	**14.75**	1.6647	148.4	0.993	2.564	0.0483	0.1552	–0.0481	0.0205	3209	1702	13.25
**94e**	**11.93**	1.7125	147.4	0.989	2.601	0.0429	0.1441	–0.0415	0.0193	3279	1688	12.46
**94f**	**15.38**	1.6324	149.1	0.999	2.542	0.0523	0.159	–0.0527	0.0209	3087	1691	13.98
**95a**	**12.59**	1.8175	144.3	0.990	2.678	0.0338	0.1216	–0.0309	0.0177	3406	1694	10.93
**95b**	**15.42**	1.6878	146.3	0.998	2.572	0.0459	0.1501	–0.0449	0.0202	3173	1681	13.13
**96a**	**11.71**	1.6879	146.3	0.991	2.572	0.0456	0.1533	–0.0452	0.0196	3330	1687	12.57
**96b**	**12.83**	1.6412	148.6	0.991	2.540	0.0511	0.1634	–0.0520	0.0207	3180	1683	13.50
**97**	**14.86**	1.5897	151.9	1.005	2.521	0.0583	0.1632	–0.0599	0.0212	2925	1724	14.71
**98**	**16.11**	1.5646	152.5	1.009	2.503	0.0622	0.1673	–0.0651	0.0217	2860	1725	15.81
**99**	**15.88**	1.5210	153.1	1.012	2.467	0.0693	0.1767	–0.0758	0.0225	2681	1610	16.99
**100**	**11.62**	1.6766	143.9	0.991	2.545	0.0471	0.1575	–0.0473	0.0227	3271	1697	13.56
**101a**	**15.14**	1.8287	144.8	0.991	2.700	0.0332	0.1127	–0.0295	0.0177	3282	1747	12.09
**101b**	**13.78**	1.7595	145.3	0.991	2.635	0.0386	0.1287	–0.0360	0.0188	3248	1691	12.76
**102**	**15.15**	1.6030	150.9	1.001	2.524	0.0563	0.1661	–0.0580	0.0214	3015	1701	14.69
**103**	**17.70**	1.5873	152.6	1.014	2.531	0.0589	0.1586	–0.0601	0.0216	2879	1698	17.71
**104**	**19.53**	1.4505	154.9	1.033	2.426	0.0841	0.1669	–0.0984	0.0213	2557	1658	17.47
**105**	**19.04**	1.5378	153.8	1.022	2.495	0.0669	0.1642	–0.0710	0.0222	2751	1673	15.85
**106**	**15.98**	1.7919	149.6	0.998	2.700	0.0359	0.1160	–0.0323	0.0185	3207	1735	12.36
**107**	**10.29**	1.9442	146.2	0.984	2.815	0.0252	0.0912	–0.0213	0.0157	3453	1761	10.06
**108a**	**11.63**	1.8911	147.5	0.988	2.775	0.0284	0.0997	–0.0246	0.0165	3370	1743	11.06
**108b**	**16.60**	1.6104	152.5	1.013	2.552	0.0555	0.1523	–0.0554	0.0215	2850	1700	15.07
**109**	**11.81**	1.7326	150.0	0.991	2.637	0.0410	0.1361	–0.0391	0.0191	3233	1701	13.61
**110a**	**16.89**	1.6386	149.0	1.002	2.551	0.0516	0.1551	–0.0514	0.0207	3006	1747	14.56
**110b**	**13.18**	1.6812	146.5	0.992	2.567	0.0463	0.1523	–0.0456	0.0200	3216	1707	13.23
**111a**	**14.46**	1.6721	148.0	0.996	2.572	0.0476	0.1507	–0.0469	0.0199	3085	1703	14.54
**111b**	**16.25**	1.6512	148.8	1.000	2.559	0.0501	0.1544	–0.0499	0.0204	3053	1682	14.99
**112a**	**14.61**	1.6392	148.9	0.999	2.547	0.0517	0.1582	–0.0519	0.0205	3021	1695	15.14
**112b**	**16.41**	1.6313	148.9	1.000	2.540	0.0527	0.1600	–0.0532	0.0207	3040	1673	15.21
**113a**	**17.98**	1.5164	153.1	1.017	2.466	0.0705	0.1757	–0.0775	0.0224	2707	1653	17.19
**113b**	**17.78**	1.5083	153.0	1.018	2.460	0.0722	0.1747	–0.0797	0.0224	2611	1653	18.45
**114a**	**16.32**	1.5353	152.0	1.010	2.473	0.0672	0.1767	–0.0728	0.0219	2721	1645	17.10
**114b**	**17.06**	1.5266	152.4	1.011	2.467	0.0686	0.1787	–0.0750	0.0221	2725	1640	17.10
**115a**	**17.49**	1.5136	152.8	1.014	2.459	0.0710	0.1785	–0.0785	0.0223	2637	1643	17.74
**115b**	**17.37**	1.5148	152.8	1.014	2.461	0.0709	0.1776	–0.0781	0.0223	2608	1648	17.81
**116a**	**17.33**	1.5165	152.8	1.014	2.462	0.0706	0.1775	–0.0777	0.0222	2613	1649	17.71
**116b**	**17.38**	1.5170	152.7	1.013	2.461	0.0704	0.1784	–0.0775	0.0222	2646	1644	17.60

**117a**	**14.5**	1.6670	148.1	0.994	2.565	0.0481	0.1548	–0.0479	0.0199	3178	1697	14.44
**117b**	**14.28**	1.6735	147.9	0.993	2.570	0.0472	0.1534	–0.0468	0.0197	3186	1699	14.24
**118a**	**15.17**	1.5841	151.1	1.002	2.508	0.0592	0.1709	–0.0620	0.0210	2922	1658	16.13
**118b**	**14.32**	1.6753	148.0	0.993	2.571	0.0472	0.1529	–0.0468	0.0197	3182	1694	14.28
**119**	**15.12**	1.5904	150.9	1.001	2.513	0.0583	0.1697	–0.0608	0.0209	2926	1650	16.10
**120**	**14.15**	1.7226	146.8	0.988	2.606	0.0421	0.1434	–0.0407	0.0186	3269	1712	12.36
**121**	**14.79**	1.6383	149.8	0.995	2.548	0.0517	0.1610	–0.0524	0.0198	3091	1678	13.83
**122**	**14.73**	1.6443	149.7	0.994	2.553	0.0510	0.1592	–0.0514	0.0197	3079	1672	13.95
**123**	**14.60**	1.6755	148.1	0.993	2.572	0.0472	0.1528	–0.0467	0.0195	3179	1700	13.73
**124**	**15.08**	1.5964	150.9	0.999	2.516	0.0574	0.1701	–0.0597	0.0206	3001	1682	15.05
**125**	**15.23**	1.5624	151.6	1.004	2.492	0.0625	0.1751	–0.0666	0.0215	2894	1638	16.17
**126**	**15.26**	1.5706	151.4	1.004	2.499	0.0613	0.1726	–0.0648	0.0213	2886	1641	16.48
**127**	**15.52**	1.6622	147.9	0.997	2.562	0.0488	0.1532	–0.0484	0.0199	3083	1699	14.35
**128**	**16.67**	1.5675	151.3	1.006	2.497	0.0618	0.1713	–0.0652	0.0213	2742	1650	16.46
**129**	**14.23**	1.6700	147.8	0.995	2.567	0.0477	0.1527	–0.0472	0.0198	3120	1707	14.53
**130**	**15.42**	1.5925	150.9	1.003	2.517	0.0580	0.1668	–0.0600	0.0208	2908	1673	15.90
**131a**	**18.56**	1.4824	154.1	1.021	2.442	0.0770	0.1779	–0.0876	0.0228	2560	1642	18.11
**131b**	**16.42**	1.4842	153.9	1.015	2.437	0.0765	0.1828	–0.0873	0.0226	2632	1643	18.06
**132**	**14.97**	1.5590	151.5	1.007	2.49	0.0633	0.1716	–0.0671	0.0214	2873	1674	16.01
**133**	**16.13**	1.5339	152.0	1.012	2.474	0.0674	0.1748	–0.0730	0.0220	2728	1652	17.14
**134**	**16.30**	1.5311	151.5	1.012	2.468	0.0679	0.1764	–0.0738	0.0220	2755	1654	17.07
**135**	**14.44**	1.6884	147.4	0.992	2.580	0.0457	0.1487	–0.0448	0.0196	3206	1689	14.20
**136**	**14.97**	1.6513	148.9	0.996	2.557	0.0501	0.1559	–0.0500	0.0202	3120	1678	14.49
**137**	**14.08**	1.6461	148.9	0.998	2.553	0.0506	0.1570	–0.0507	0.0201	3120	1684	14.16
**138a**	**10.59**	1.7830	145.0	0.979	2.645	0.0364	0.1326	–0.0343	0.0172	3471	1746	10.18
**138b**	**12.40**	1.7194	146.7	0.983	2.598	0.0422	0.1471	–0.0413	0.0185	3352	1702	11.84
**139a**	**11.60**	1.7102	147.2	0.984	2.593	0.0432	0.1487	–0.0424	0.0183	3378	1719	11.07
**139b**	**13.33**	1.6401	149.1	0.989	2.539	0.0511	0.1658	–0.0523	0.0196	3215	1679	12.84
**140**	**12.01**	1.6442	149.1	0.989	2.543	0.0505	0.1651	–0.0516	0.0194	3217	1664	13.13
**141**	**12.93**	1.7449	143.7	0.989	2.608	0.0394	0.1387	–0.0377	0.0191	3225	1752	12.40
**142**	**12.59**	1.6635	144.7	0.993	2.540	0.0488	0.1588	–0.0493	0.0227	3208	1686	14.56
**143**	**10.38**	1.7765	148.9	0.987	2.670	0.0369	0.1274	–0.0345	0.0184	3323	1708	12.49
**144**	**13.83**	1.7711	145.7	0.990	2.649	0.0378	0.1274	–0.0351	0.0183	3235	1688	13.33
**145**	**15.72**	1.7189	149.0	0.996	2.624	0.0425	0.1372	–0.0405	0.0195	3198	1702	13.79
**146**	**15.60**	1.6966	147.3	0.997	2.592	0.0452	0.1427	–0.0435	0.0196	3105	1674	14.29
**147**	**16.28**	1.6438	151.7	1.002	2.570	0.0510	0.1524	–0.0507	0.0209	3013	1684	15.22
**148**	**13.72**	1.6538	149.4	0.993	2.559	0.0497	0.1575	–0.0499	0.0202	3133	1731	14.32
**149**	**17.44**	1.6006	149.3	1.002	2.514	0.0568	0.1680	–0.0588	0.0211	3030	1681	15.00
**150**	**15.20**	1.6185	151.0	1.000	2.540	0.0542	0.1615	–0.0551	0.0207	3022	1697	15.36
**151**	**15.13**	1.6099	151.6	1.001	2.536	0.0554	0.1621	–0.0566	0.0208	2973	1688	15.73
**152**	**14.16**	1.6440	150.2	0.995	2.556	0.0510	0.1569	–0.0511	0.0202	3101	1651	14.57
**153**	**14.15**	1.6333	150.9	0.997	2.551	0.0524	0.1586	–0.0528	0.0204	2995	1684	15.35
**154**	**15.33**	1.6030	152.0	1.002	2.532	0.0564	0.1629	–0.0578	0.0209	2946	1671	15.73
**155**	**16.63**	1.5104	152.8	1.015	2.457	0.0717	0.1769	–0.0792	0.0224	2649	1646	17.64
**156**	**17.03**	1.4992	152.7	1.016	2.446	0.0737	0.1795	–0.0825	0.0225	2650	1641	18.15
**157**	**16.04**	1.5956	151.3	1.003	2.522	0.0573	0.1670	–0.0593	0.0212	2946	1651	16.31
**158**	**15.79**	1.5923	151.6	1.003	2.520	0.0579	0.1667	–0.0599	0.0212	2899	1655	16.59
**159**	**16.01**	1.5998	151.7	1.003	2.528	0.0570	0.1642	–0.0587	0.0211	2920	1651	16.45
**160**	**15.68**	1.5975	151.5	1.004	2.525	0.0573	0.1645	–0.0590	0.0211	2941	1657	16.32
**161**	**16.09**	1.6257	150.1	1.000	2.542	0.0532	0.1617	–0.0541	0.0206	3030	1647	15.87
**162**	**16.03**	1.6391	149.9	0.999	2.553	0.0515	0.1587	–0.0519	0.0204	3084	1649	15.15
**163**	**15.63**	1.5935	151.0	1.004	2.519	0.0577	0.1662	–0.0596	0.0211	2905	1661	16.61
**164**	**15.99**	1.5952	151.5	1.002	2.522	0.0574	0.1664	–0.0593	0.0212	2924	1647	16.59
**165**	**15.96**	1.6092	150.7	1.002	2.531	0.0556	0.1642	–0.0570	0.0208	2981	1655	16.02

**166**	**15.81**	1.5976	151.1	1.003	2.522	0.0571	0.1662	–0.0589	0.0211	2946	1646	16.33
**167**	**14.33**	1.6212	149.7	1.000	2.531	0.0532	0.1652	–0.0543	0.0203	3084	1584	15.30
**168**	**15.93**	1.5715	152.4	1.005	2.506	0.0610	0.1703	–0.0641	0.0216	2897	1626	16.60
**169**	**18.01**	1.5598	151.0	1.013	2.495	0.0589	0.1672	–0.0611	0.0213	2875	1623	16.77
**170**	**18.31**	1.5836	150.5	1.009	2.511	0.0592	0.1658	–0.0613	0.0214	2900	1705	16.09
**171**	**21.89**	1.4279	155.6	1.039	2.412	0.0891	0.1616	–0.1069	0.0236	2430	1630	19.13
**172**	**16.67**	1.5270	171.2	1.020	2.540	0.0665	0.1619	–0.0693	0.0107	2619	1622	16.13
**173**	**17.20**	1.4633	174.0	1.029	2.489	0.0790	0.1642	–0.0889	0.0113	2369	1610	17.69
**174**	**17.96**	1.4506	174.2	1.033	2.481	0.0818	0.1596	–0.0934	0.0114	2273	1621	18.45
**175**	**16.39**	1.4821	172.4	1.024	2.501	0.0746	0.1669	–0.0821	0.011	2468	1615	17.17
**176**	**17.96**	1.4448	172.7	1.034	2.474	0.0824	0.1610	–0.0948	0.0113	2144	1622	18.75
**177**	**13.47**	1.6862	147.5	0.990	2.577	0.0454	0.1514	–0.0446	0.0191	3249	1682	14.68
**178**	**18.28**	1.5878	149.9	1.009	2.512	0.0577	0.1646	–0.0592	0.0211	2957	1716	14.68
**179**	**13.92**	1.7190	145.2	0.985	2.589	0.0420	0.1471	–0.0409	0.0183	3325	1702	11.64
**180**	**13.84**	1.6518	149.4	0.993	2.557	0.0493	0.1577	–0.0493	0.0196	3180	1687	14.21
**181**	**14.65**	1.6027	147.3	0.994	2.497	0.0558	0.1751	–0.0582	0.0207	3179	1675	14.45
**182**	**13.54**	1.6047	149.1	0.993	2.509	0.0552	0.1723	–0.0573	0.0205	3112	1580	14.80
**183**	**13.78**	1.6318	150.4	0.996	2.546	0.0517	0.1611	–0.0522	0.0199	3130	1579	14.85
**184**	**15.91**	1.5556	151.3	1.004	2.483	0.0624	0.1787	–0.0666	0.0212	3019	1601	15.88
**185**	**12.91**	1.6921	146.5	0.988	2.575	0.0447	0.1517	–0.0440	0.0191	3260	1675	13.39
**186**	**11.69**	1.6625	148.2	0.989	2.557	0.0479	0.1581	–0.0481	0.0197	3240	1598	13.78
**187**	**0.56**	2.5014	138.1	0.967	3.285	0.0075	0.0353	–0.0060	0.0075	3756	1831	5.24
**188**	**6.90**	2.4240	134.3	0.970	3.178	0.0092	0.0366	–0.0069	0.0086	3479	1709	9.10

Table S18. Characteristics of the O–H···O=C hydrogen bond for studied compounds from the arom-AHB cluster. The $-E_{HB}$ MTA energy of intramolecular hydrogen bonding (kcal/mol), the $r_{O\cdots H}$ hydrogen bond length (Å), the d_{OH} length of the O–H bond (Å), the ϑ_{HB} hydrogen bond angle (degree), the ρ_{BCP} and $\nabla^2\rho$ electron density at the bond critical point and its Laplacian, respectively (a.u.), the V_{BCP} potential energy density at the bond critical point (a.u.), the ρ_{RCP} electron density at the ring critical point, the ν_{OH} frequency of O–H stretching (cm^{-1}), the δ_{OH} ^{1}H NMR chemical shifts (ppm).

**No**	**$-E_{HB}$**	$r_{O\cdots H}$	ϕ_{HB}	d_{OH}	$r_{O\cdots O}$	ρ_{BCP}	$\nabla^2\rho$	V_{BCP}	ρ_{RCP}	ν_{OH}	$\nu_{C=O}$	δ_{OH}
**1**	**7.85**	1.7724	147.4	0.9791	2.650	0.0372	0.1328	–0.0352	0.0176	3427	1706	11.62
**2**	**8.57**	1.7003	148.3	0.9821	2.588	0.0443	0.1513	–0.0436	0.0186	3333	1684	12.65
**3**	**8.20**	1.7207	147.6	0.9806	2.602	0.0349	0.1551	–0.0410	0.0180	3352	1664	12.66
**4**	**6.82**	1.7610	146.7	0.9770	2.633	0.0380	0.1381	–0.0363	0.0179	3474	1728	11.24
**5**	**7.09**	1.7516	147.2	0.9779	2.627	0.0390	0.1403	–0.0374	0.0179	3462	1709	11.08
**6**	**7.23**	1.7474	147.4	0.9781	2.625	0.0394	0.1411	–0.0379	0.0180	3458	1705	11.16
**7**	**6.99**	1.7637	146.7	0.9769	2.635	0.0379	0.1378	–0.0362	0.0180	3471	1723	10.91
**8**	**8.63**	1.6980	148.6	0.9829	2.588	0.0443	0.1509	–0.0436	0.0188	3317	1699	12.46
**9**	**8.06**	1.7610	146.8	0.9794	2.635	0.0382	0.1355	–0.0363	0.0185	3362	1684	11.68
**10**	**7.99**	1.7157	148.0	0.9812	2.600	0.0424	0.1475	–0.0414	0.0186	3357	1689	12.45
**11**	**7.83**	1.7839	146.3	0.9783	2.654	0.0363	0.1303	–0.0341	0.0184	3428	1668	10.91
**12**	**6.98**	1.7375	146.8	0.9778	2.611	0.0402	0.1440	–0.0400	0.0181	3459	1679	10.99
**13**	**7.20**	1.7289	147.3	0.9785	2.606	0.0411	0.1458	–0.0400	0.0182	3446	1670	11.47
**14**	**5.69**	1.8195	144.8	0.9732	2.674	0.0333	0.1260	–0.0309	0.0173	3560	1795	9.78
**15**	**5.72**	1.7740	144.8	0.9738	2.631	0.0369	0.1376	–0.0352	0.0179	3542	1751	10.14
**16**	**5.53**	1.7755	144.5	0.9732	2.629	0.0367	0.1345	–0.0351	0.0179	3558	1757	9.91
**17**	**6.64**	1.7720	146.2	0.9767	2.640	0.0371	0.1260	–0.0355	0.0175	3456	1673	11.49
**18**	**5.38**	1.8303	144.4	0.9729	2.682	0.0322	0.1242	–0.0299	0.0170	3568	1768	10.11
**19**	**7.88**	1.7781	147.2	0.9788	2.654	0.0368	0.1318	–0.0347	0.0175	3426	1704	11.54
**20**	**7.56**	1.7840	147.0	0.9778	2.658	0.0362	0.1311	–0.0341	0.0173	3453	1709	11.09
**21**	**7.92**	1.7808	147.0	0.9781	2.655	0.0365	0.1317	–0.0344	0.0174	3446	1709	11.18
**22**	**7.74**	1.7865	147.0	0.9777	2.660	0.0360	0.1305	–0.0339	0.0173	3457	1707	11.00
**23**	**7.67**	1.7898	146.9	0.9775	2.662	0.0358	0.1298	–0.0336	0.0173	3464	1706	11.02

**24**	**7.84**	1.7769	147.0	0.9784	2.652	0.0368	0.1324	–0.0348	0.0175	3438	1712	11.38
**25**	**7.75**	1.7763	147.0	0.9788	2.652	0.0369	0.1323	–0.0348	0.0175	3429	1711	11.40
**26**	**7.75**	1.7757	147.1	0.9789	2.651	0.0370	0.1323	–0.0349	0.0175	3429	1710	11.53
**27**	**7.74**	1.7683	147.4	0.9800	2.647	0.0376	0.1333	–0.0356	0.0176	3387	1710	12.11
**28**	**7.87**	1.7709	147.1	0.9795	2.647	0.0374	0.1334	–0.0354	0.0176	3419	1710	11.67
**29**	**7.97**	1.7713	147.0	0.9799	2.648	0.0373	0.1326	–0.0353	0.0176	3375	1714	12.30
**30**	**8.11**	1.7746	147.5	0.9794	2.653	0.0371	0.1320	–0.0350	0.0176	3406	1699	11.62
**31**	**8.65**	1.7597	148.0	0.9811	2.644	0.0384	0.1343	–0.0364	0.0178	3358	1693	11.93
**32**	**8.09**	1.7690	148.1	0.9800	2.652	0.0376	0.1327	–0.0355	0.0177	3400	1696	11.55
**33**	**8.71**	1.7601	148.4	0.9811	2.646	0.0384	0.1341	–0.0364	0.0179	3349	1685	11.88
**34**	**8.83**	1.7579	148.8	0.9816	2.646	0.0387	0.1341	–0.0367	0.0179	3358	1681	11.77
**35**	**8.28**	1.7648	147.7	0.9804	2.646	0.0379	0.1339	–0.0359	0.0177	3366	1703	11.96
**36**	**8.11**	1.7647	147.6	0.9803	2.645	0.0379	0.1342	–0.0360	0.0177	3400	1704	11.84
**37**	**7.99**	1.7662	147.5	0.9802	2.646	0.0378	0.1340	–0.0350	0.0177	3403	1704	11.68
**38**	**7.87**	1.7714	147.0	0.9796	2.648	0.0373	0.1335	–0.0353	0.0176	3432	1717	11.61
**39**	**7.82**	1.7761	146.7	0.9791	2.649	0.0369	0.1328	–0.0349	0.0175	3431	1716	11.40
**40**	**8.85**	1.7310	148.3	0.9827	2.619	0.0411	0.1414	–0.0396	0.0182	3331	1699	12.46
**41**	**9.44**	1.7067	149.3	0.9848	2.603	0.0437	0.1460	–0.0425	0.0187	3263	1683	12.83
**42**	**8.60**	1.7257	148.5	0.9818	2.614	0.0417	0.1434	–0.0404	0.0183	3323	1692	12.55
**43**	**8.93**	1.7164	148.3	0.9838	2.606	0.0426	0.1449	–0.0414	0.0185	3293	1695	12.99
**44**	**11.28**	1.6523	150.7	0.9933	2.565	0.0498	0.1541	–0.0496	0.0198	3077	1687	14.47
**45**	**8.05**	1.7527	145.8	0.9835	2.625	0.0389	0.1366	–0.0370	0.0178	3354	1736	12.71
**46**	**8.07**	1.7668	145.4	0.9819	2.635	0.0343	0.1343	–0.0356	0.0176	3384	1738	12.64
**47**	**10.45**	1.7251	152.9	0.9851	2.640	0.0420	0.1387	–0.0402	0.0187	3311	1633	11.29
**48**	**7.72**	1.7937	149.1	0.9758	2.678	0.0355	0.1288	–0.0333	0.0171	3497	1661	9.57
**49**	**8.39**	1.6447	148.6	0.9828	2.536	0.0505	0.1678	–0.0518	0.0195	3276	1671	13.23
**50**	**9.25**	1.6218	149.7	0.9869	2.523	0.0536	0.1710	–0.0555	0.0198	3201	1620	13.86
**51**	**9.41**	1.6089	149.8	0.9883	2.513	0.0554	0.1741	–0.0579	0.0200	3181	1665	14.09
**52**	**9.56**	1.6210	149.9	0.9877	2.524	0.0538	0.1704	–0.0556	0.0198	3187	1674	13.83
**53**	**9.41**	1.6866	148.9	0.9847	2.581	0.0458	0.1527	–0.0453	0.0189	3271	1674	12.90
**54**	**8.81**	1.6988	148.8	0.9827	2.590	0.0445	0.1507	–0.0437	0.0186	3315	1678	12.28
**55**	**9.17**	1.6823	149.2	0.9846	2.578	0.0462	0.1541	–0.0460	0.0189	3282	1672	13.16
**56**	**8.48**	1.7106	148.4	0.9820	2.598	0.0431	0.1479	–0.0422	0.0186	3320	1643	12.68
**57**	**8.32**	1.7253	147.4	0.9807	2.606	0.0416	0.1450	–0.0405	0.0184	3347	1665	12.59
**58**	**9.17**	1.6600	149.1	0.9850	2.556	0.0487	0.1611	–0.0492	0.0192	3248	1686	13.86
**59**	**9.90**	1.6321	149.5	0.9881	2.534	0.0521	0.1661	–0.0534	0.0200	3071	1596	14.92
**60**	**7.96**	1.6797	148.1	0.9811	2.566	0.0458	0.1581	–0.0458	0.0184	3342	1584	12.91
**61**	**6.36**	1.7805	144.9	0.9748	2.638	0.0361	0.1342	–0.0343	0.0176	3478	1663	11.50
**62**	**7.24**	1.9741	145.4	0.9750	2.832	0.0235	0.0887	–0.0200	0.0145	3560	1727	9.65
**63**	**8.77**	1.6965	148.6	0.9830	2.587	0.0447	0.1516	–0.0441	0.0187	3318	1682	12.81
**64**	**8.98**	1.7133	149.7	0.9854	2.612	0.0429	0.1441	–0.0417	0.0184	3307	1692	12.57
**65**	**9.36**	1.6899	149.2	0.9846	2.585	0.0454	0.1516	–0.0448	0.0189	3271	1670	12.82
**66**	**9.57**	1.6837	149.3	0.9857	2.581	0.0461	0.1526	–0.0456	0.0190	3253	1672	12.98
**67**	**9.56**	1.7089	147.7	0.9829	2.593	0.0433	0.1494	–0.0425	0.0186	3355	1686	12.29
**68**	**8.78**	1.6783	149.2	0.9849	2.574	0.0467	0.1556	–0.0466	0.0189	3283	1659	13.04
**69**	**10.19**	1.6071	150.5	0.9903	2.516	0.0557	0.1719	–0.0579	0.0201	3127	1650	14.15
**70**	**13.11**	1.5010	154.6	1.0098	2.452	0.0733	0.1819	–0.0821	0.0221	2706	1630	17.22
**71**	**6.96**	1.7669	146.3	0.9766	2.636	0.0375	0.1370	–0.0357	0.0177	3483	1732	10.55
**72**	**6.87**	1.7650	146.3	0.9770	2.634	0.0377	0.1372	–0.0359	0.0178	3479	1732	10.73
**73**	**6.87**	1.7645	146.3	0.9770	2.634	0.0377	0.1375	–0.0359	0.0178	3478	1731	10.83
**74**	**8.31**	1.7246	147.8	0.9815	2.608	0.0416	0.1457	–0.0406	0.0182	3371	1689	12.36
**75**	**8.68**	1.7220	148.0	0.9822	2.607	0.0420	0.1457	–0.0409	0.0182	3358	1691	12.34
**76**	**8.48**	1.7240	147.9	0.9820	2.609	0.0417	0.1453	–0.0406	0.0182	3361	1689	12.36
**77**	**9.89**	1.6772	149.5	0.9873	2.577	0.0468	0.1543	–0.0465	0.0189	3255	1655	12.99

**78a**	**8.49**	1.7075	147.8	0.9823	2.592	0.0433	0.1501	–0.0427	0.0185	3356	1665	12.37
**78b**	**8.47**	1.7040	148.0	0.9825	2.590	0.0437	0.1508	–0.0431	0.0185	3348	1662	12.44
**78c**	**8.84**	1.7089	147.5	0.9826	2.592	0.0431	0.1500	–0.0425	0.0184	3364	1667	12.45
**79**	**9.27**	1.6923	148.6	0.9846	2.584	0.0450	0.1527	–0.0446	0.0187	3322	1656	12.78
**80**	**8.67**	1.6954	148.4	0.9834	2.585	0.0447	0.1524	–0.0442	0.0187	3332	1663	12.67
**81**	**8.86**	1.7156	148.3	0.9831	2.604	0.0426	0.1468	–0.0416	0.0184	3335	1675	12.51
**82**	**8.37**	1.7188	147.9	0.9818	2.603	0.0423	0.1475	–0.0414	0.0184	3375	1696	12.33
**83**	**10.46**	1.7012	148.2	0.9849	2.591	0.0440	0.1508	–0.0434	0.0186	3315	1699	12.79
**84a**	**11.73**	1.6763	149.3	0.9888	2.576	0.0468	0.1533	–0.0466	0.0192	3233	1679	13.45
**84b**	**11.78**	1.6727	149.2	0.9885	2.572	0.0471	0.1572	–0.0472	0.0193	3257	1679	13.20
**85a**	**10.28**	1.7001	147.6	0.9840	2.585	0.0440	0.1524	–0.0436	0.0188	3339	1689	12.65
**85b**	**10.71**	1.6974	148.2	0.9856	2.588	0.0444	0.1516	–0.0439	0.0187	3307	1689	12.66
**86a**	**10.29**	1.7038	148.1	0.9851	2.593	0.0437	0.1499	–0.0430	0.0186	3309	1695	12.64
**86b**	**10.66**	1.6814	148.8	0.9871	2.577	0.0461	0.1552	–0.0460	0.0191	3263	1695	13.20
**87**	**10.62**	1.6920	147.7	0.9850	2.579	0.0449	0.1543	–0.0448	0.0190	3335	1687	12.77
**88**	**10.64**	1.6753	147.4	0.9928	2.568	0.0468	0.1561	–0.0468	0.0192	3245	1693	13.56
**89a**	**10.56**	1.6981	148.2	0.9860	2.589	0.0443	0.1511	–0.0437	0.0184	3305	1684	12.69
**89b**	**10.40**	1.6799	148.3	0.9861	2.572	0.0462	0.1570	–0.0462	0.0192	3305	1684	13.32
**90a**	**10.89**	1.6755	148.9	0.9881	2.572	0.0467	0.1565	–0.0467	0.0192	3259	1683	13.24
**90b**	**10.15**	1.7009	147.7	0.9844	2.587	0.0439	0.1519	–0.0435	0.0188	3337	1683	12.54
**91a**	**10.61**	1.6923	147.8	0.9854	2.580	0.0449	0.1538	–0.0446	0.0190	3331	1680	12.67
**91b**	**10.98**	1.6702	148.4	0.9874	2.564	0.0473	0.1589	–0.0476	0.0194	3287	1681	13.35
**92a**	**10.45**	1.6747	148.7	0.9872	2.570	0.0474	0.1573	–0.0469	0.0193	3275	1679	13.35
**92b**	**10.77**	1.6699	149.2	0.9891	2.570	0.0467	0.1576	–0.0475	0.0194	3236	1680	13.54
**93**	**10.72**	1.6639	148.9	0.9885	2.561	0.0480	0.1601	–0.0484	0.0196	3265	1674	13.55
**94**	**8.82**	1.6942	148.2	0.9832	2.583	0.0448	0.1532	–0.0444	0.0187	3336	1675	13.02
**95**	**10.03**	1.6869	147.5	0.9871	2.574	0.0456	0.1522	–0.0450	0.0188	3284	1673	13.33
**96**	**9.07**	1.6904	148.3	0.9840	2.580	0.0452	0.1539	–0.0449	0.0188	3333	1668	13.12
**97**	**10.53**	1.6767	148.4	0.9856	2.569	0.0467	0.1573	–0.0468	0.0191	3300	1684	13.21
**98**	**7.59**	1.7054	147.1	0.9798	2.583	0.0432	0.1526	–0.0427	0.0182	3410	1661	12.45
**99**	**9.87**	1.6819	147.4	0.9828	2.565	0.0457	0.1579	–0.0457	0.0187	3372	1681	12.71
**100a**	**9.26**	1.6952	146.5	0.9844	2.574	0.0442	0.1518	–0.0436	0.0184	3332	1645	12.86
**100b**	**9.46**	1.6964	147.2	0.9813	2.576	0.0441	0.1549	–0.0438	0.0184	3389	1645	12.35
**100c**	**11.48**	1.6612	148.9	0.9879	2.558	0.0485	0.1601	–0.0489	0.0193	3259	1682	13.49
**101a**	**11.25**	1.6614	148.8	0.9881	2.558	0.0484	0.1613	–0.0490	0.0195	3289	1680	13.25
**101b**	**10.87**	1.6484	148.9	0.9882	2.546	0.0501	0.1631	–0.0509	0.0196	3214	1657	13.77
**101c**	**5.60**	1.7569	145.0	0.9755	2.616	0.0383	0.1409	–0.0369	0.0187	3419	1719	11.43
**102**	**5.47**	1.7568	145.1	0.9732	2.615	0.0382	0.1430	–0.0370	0.0181	3474	1670	11.18
**103**	**5.64**	2.1460	142.0	0.9723	2.973	0.0161	0.0628	–0.0128	0.0119	3654	1769	7.94
**104**	**5.82**	2.1172	143.3	0.9712	2.954	0.0169	0.0668	–0.0137	0.0122	3652	1606	8.50
**105**	**6.68**	2.0627	144.1	0.9728	2.907	0.0192	0.0745	–0.0158	0.0130	3614	1735	8.91
**106**	**7.34**	1.6954	147.5	0.9797	2.576	0.0449	0.1548	–0.0440	0.0184	3393	1670	12.67
**107**	**7.44**	1.7298	148.2	0.9803	2.613	0.0409	0.1437	–0.0396	0.0179	3416	1636	12.15
**108**	**7.55**	1.7000	147.8	0.9790	2.582	0.0430	0.1533	–0.0424	0.0179	3405	1601	12.27
**109**	**7.69**	1.7014	148.5	0.9806	2.588	0.0431	0.1514	–0.0424	0.0180	3384	1609	12.46
**110**	**8.24**	1.7339	148.3	0.9811	2.620	0.0408	0.1419	–0.0394	0.0181	3368	1690	12.07
**111**	**8.25**	1.7690	149.2	0.9821	2.660	0.0374	0.1308	–0.0352	0.0177	3390	1627	11.75
**112**	**8.36**	1.7662	149.1	0.9823	2.658	0.0376	0.1314	–0.0354	0.0177	3387	1683	11.88
**113**	**8.69**	1.7323	148.5	0.9878	2.619	0.0410	0.1425	–0.0397	0.0182	3367	1670	12.21
**114**	**8.73**	1.7100	149.4	0.9843	2.606	0.0436	0.1461	–0.0422	0.0183	3321	1680	12.95
**115**	**8.75**	1.6876	149.1	0.9828	2.581	0.0451	0.1534	–0.0447	0.0186	3354	1676	12.83
**116**	**9.32**	1.7136	148.1	0.9831	2.601	0.0428	0.1479	–0.0420	0.0186	3335	1694	12.81
**117**	**9.32**	1.7098	150.0	0.9864	2.611	0.0434	0.1442	–0.0421	0.0185	3286	1638	12.76
**118**	**9.84**	1.7032	150.5	0.9881	2.609	0.0441	0.1445	–0.0428	0.0187	3252	1694	12.78

**119**	**15.41**	1.4690	152.0	1.0175	2.415	0.0790	0.1852	−0.0913	0.0225	2745	1711	16.93
**120**	**9.95**	1.6951	150.3	0.9874	2.599	0.0449	0.1479	−0.0439	0.0188	3274	1689	12.91
**121**	**11.09**	1.6562	151.4	0.9921	2.572	0.0494	0.1552	−0.0493	0.0193	3182	1681	13.65
**122**	**11.20**	1.6654	149.3	0.9887	2.565	0.0481	0.1565	−0.0482	0.0195	3223	1678	13.91
**123**	**9.86**	1.6490	150.3	0.9870	2.554	0.0501	0.1618	−0.0507	0.0194	3222	1665	13.63

Table S19. The calculated E_{π}(PPE) energy (kcal mol^{-1}), bond length (l, Å), vibration frequency (ν, cm^{-1}), HOMO and LUMO energy (E(HOMO) and E(LUMO), eV), HOMO−LUMO energy gap ($\Delta E_{\text{HOMO-LUMO}}$, eV) for subseries **Ia** and **Ib** of studied compounds **1 – 18**.

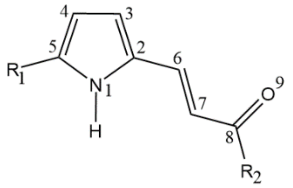

N subseries	N compound	$-E_{\pi}$(PPE)	Bond lengths				Freq	MO energy		
			$l(C_2–C_6)$	$l(C_6=C_7)$	$l(C_7–C_8)$	$l(C_8=O_9)$	$\nu_{C=O}$	E(HOMO)	E(LUMO)	ΔE(HOMO−LUMO)
**Ia**	**1**	12.06	1.4043	1.3724	1.4260	1.1941	1843	−0.211	−0.102	0.110
	**2**	10.60	1.4073	1.3697	1.4290	1.1931	1847	−0.222	−0.107	0.115
	**3**	8.79	1.4120	1.3657	1.4335	1.1918	1851	−0.235	−0.114	0.121
	**4**	7.98	1.4166	1.3639	1.4353	1.1910	1854	−0.241	−0.117	0.124
	**5**	6.92	1.4202	1.3614	1.4383	1.1901	1858	−0.250	−0.121	0.128
	**6**	6.31	1.4194	1.3609	1.4391	1.1900	1857	−0.251	−0.125	0.125
	**7**	4.31	1.4268	1.3571	1.4450	1.1884	1863	−0.267	−0.142	0.124
	**8**	3.73	1.4277	1.3561	1.4464	1.1882	1864	−0.270	−0.142	0.127
	**9**	3.04	1.4302	1.3542	1.4497	1.1875	1866	−0.281	−0.154	0.127
**Ib**	**10**	3.59	1.4243	1.3549	1.4790	1.2317	1697	−0.183	−0.048	0.135
	**11**	3.42	1.4234	1.3537	1.4751	1.2251	1732	−0.187	−0.050	0.136
	**12**	5.17	1.4189	1.3576	1.4565	1.2154	1763	−0.181	−0.059	0.122
	**13**	4.96	1.4189	1.3591	1.4691	1.2246	1727	−0.196	−0.063	0.133
	**14**	5.88	1.4153	1.3631	1.4559	1.2216	1733	−0.193	−0.069	0.124
	**15**	8.90	1.4107	1.3665	1.4418	1.1919	1715	−0.204	−0.080	0.124
	**16**	8.77	1.4105	1.3663	1.4439	1.2297	1700	−0.203	−0.099	0.104
	**17**	10.22	1.4066	1.3692	1.4447	1.2238	1697	−0.208	−0.096	0.111
	**18**	12.06	1.4043	1.3724	1.4260	1.1941	1842	−0.212	−0.102	0.110

Table S20. The calculated E_{π}(PPE) energy (kcal mol^{-1}), bond length (*l*, Å), vibration frequency (*ν*, cm^{-1}), HOMO and LUMO energy (*E*(HOMO) and *E*(LUMO), eV), HOMO–LUMO energy gap ($\Delta E_{HOMO\text{-}LUMO}$, eV) for subseries **IIa** and **IIb** of studied compounds **19 – 36**.

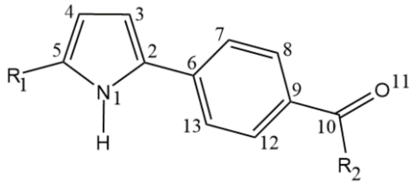

**N subseries**	**N compound**	$-E_{\pi}$(PPE)	Bond lengths									Freq	MO energy		
			$l(C_2–C_6)$	$l(C_6–C_7)$	$l(C_7=C_8)$	$l(C_8–C_9)$	$l(C_9–C_{10})$	$l(C_{10}=O_{11})$	$l(C_9=C_{12})$	$l(C_{12}–C_{13})$	$l(C_6=C_{13})$	$\nu_{C=O}$	*E*(HOMO)	*E*(LUMO)	Δ*E*(HOMO–LUMO)
**IIa**	**19**	3.44	1.4382	1.4175	1.3762	1.4112	1.4519	1.1926	1.4046	1.3822	1.4143	1815	−0.202	−0.094	0.108
	**20**	2.71	1.4423	1.4155	1.3775	1.4098	1.4544	1.1944	1.4040	1.3829	1.4124	1819	−0.212	−0.099	0.114
	**21**	2.43	1.4441	1.4149	1.3777	1.4092	1.4558	1.1940	1.4033	1.3834	1.4114	1821	−0.220	−0.101	0.119
	**22**	2.22	1.4481	1.4136	1.3787	1.4084	1.4571	1.1936	1.4031	1.3838	1.4106	1824	−0.226	−0.103	0.123
	**23**	1.56	1.4511	1.4121	1.3796	1.4075	1.4590	1.1930	1.4027	1.3843	1.4094	1827	−0.234	−0.106	0.128
	**24**	1.11	1.4509	1.4118	1.3797	1.4074	1.4649	1.1927	1.4024	1.3846	1.4089	1828	−0.236	−0.110	0.126
	**25**	−0.05	1.4560	1.4095	1.3814	1.4057	1.4642	1.1915	1.4017	1.3854	1.4072	1834	−0.252	−0.123	0.129
	**26**	−0.30	1.4570	1.4090	1.3815	1.4055	1.4649	1.1914	1.4015	1.3858	1.4066	1834	−0.255	−0.123	0.132
	**27**	−0.51	1.4581	1.4081	1.3821	1.4048	1.4669	1.1910	1.4013	1.3860	1.4058	1837	−0.267	−0.134	0.133
**IIb**	**28**	0.40	1.4550	1.4088	1.3854	1.4013	1.5016	1.2266	1.3983	1.3903	1.4070	1690	−0.186	−0.045	0.142
	**29**	0.59	1.4528	1.4098	1.3839	1.4021	1.4960	1.2218	1.3997	1.3894	1.4077	1736	−0.189	−0.056	0.133
	**30**	1.05	1.4503	1.4113	1.3825	1.4033	1.4770	1.2115	1.4003	1.3870	1.4096	1773	−0.190	−0.064	0.126
	**31**	1.01	1.4506	1.4120	1.3816	1.4059	1.4912	1.2198	1.4012	1.3887	1.4082	1730	−0.190	−0.067	0.123
	**32**	1.30	1.4485	1.4149	1.3803	1.4059	1.4705	1.2146	1.3995	1.3873	1.4098	1749	−0.193	−0.073	0.119
	**33**	2.38	1.4434	1.4144	1.3787	1.4103	1.4676	1.1893	1.4013	1.3846	1.4117	1824	−0.196	−0.082	0.113
	**34**	2.28	1.4429	1.4159	1.3772	1.4116	1.4693	1.2251	1.4064	1.3857	1.4110	1692	−0.192	−0.103	0.089
	**35**	3.15	1.4395	1.4181	1.3765	1.4101	1.4633	1.2182	1.4039	1.3835	1.4133	1701	−0.198	−0.100	0.098
	**36**	3.45	1.4382	1.4175	1.3762	1.4112	1.4519	1.1953	1.4046	1.3821	1.4143	1815	−0.200	−0.094	0.106

Table S21. The calculated E_{π}(PPE) energy (kcal mol^{-1}), bond length (l, Å), vibration frequency (v, cm^{-1}), HOMO and LUMO energy (E(HOMO) and E(LUMO), eV), HOMO–LUMO energy gap ($\Delta E_{HOMO\text{-}LUMO}$, eV) for subseries IIIa and IIIb of studied compounds **37 – 54**.

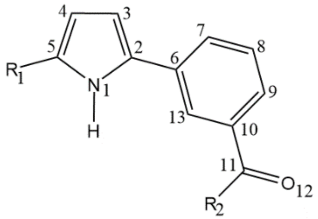

N subseries	N compound	$-E_{\pi}$(PPE)	Bond lengths									Freq	MO energy		
			$l(C_2–C_6)$	$l(C_6–C_7)$	$l(C_7=C_8)$	$l(C_8–C_9)$	$l(C_9=C_{10})$	$l(C_{10}–C_{11})$	$l(C_{11}=O_{12})$	$l(C_{10}–C_{13})$	$l(C_6=C_{13})$	$v_{C=O}$	E(HOMO)	E(LUMO)	ΔE(HOMO–LUMO)
**IIIa**	**37**	2.12	1.4526	1.4098	1.3902	1.3903	1.4025	1.4677	1.1901	1.3994	1.4034	1843	−0.196	−0.104	0.093
	**38**	1.84	1.4535	1.4093	1.3905	1.3900	1.4024	1.4680	1.1901	1.3994	1.4030	1842	−0.205	−0.105	0.100
	**39**	1.39	1.4544	1.4090	1.3903	1.3899	1.4021	1.4686	1.1899	1.3996	1.4026	1843	−0.212	−0.106	0.106
	**40**	1.50	1.4574	1.4081	1.3910	1.3895	1.4023	1.4684	1.1898	1.3995	1.4019	1843	−0.218	−0.108	0.110
	**41**	1.09	1.4595	1.4071	1.3914	1.3892	1.4023	1.4689	1.1896	1.3995	1.4012	1844	−0.227	−0.110	0.117
	**42**	0.51	1.4592	1.4070	1.3911	1.3893	1.4021	1.4698	1.1891	1.3994	1.4010	1845	−0.229	−0.113	0.116
	**43**	−0.26	1.4622	1.4058	1.3916	1.3890	1.4023	1.4712	1.1891	1.3989	1.4001	1848	−0.247	−0.122	0.125
	**44**	−0.82	1.4631	1.4054	1.3916	1.3889	1.4021	1.4718	1.1894	1.3990	1.3998	1847	−0.249	−0.122	0.127
	**45**	−1.50	1.4633	1.4051	1.3914	1.3887	1.4019	1.4727	1.2270	1.3988	1.3999	1846	−0.262	−0.124	0.138
**IIIb**	**46**	0.37	1.4573	1.4070	1.3892	1.3937	1.3966	1.5068	1.2216	1.3968	1.4055	1699	−0.183	−0.040	0.142
	**47**	0.50	1.4561	1.4065	1.3900	1.3927	1.3973	1.5034	1.2104	1.3978	1.4051	1739	−0.184	−0.051	0.133
	**48**	1.02	1.4564	1.4091	1.3890	1.3938	1.3985	1.4861	1.2180	1.3971	1.4021	1779	−0.186	−0.062	0.124
	**49**	1.19	1.4564	1.4099	1.3879	1.3951	1.3998	1.5010	1.2122	1.3989	1.4013	1740	−0.185	−0.067	0.118
	**50**	1.19	1.4554	1.4126	1.3881	1.3948	1.3975	1.4798	1.1882	1.3985	1.4009	1761	−0.188	−0.075	0.114
	**51**	1.39	1.4548	1.4103	1.3880	1.3936	1.3980	1.4824	1.2201	1.4021	1.4010	1830	−0.193	−0.086	0.107
	**52**	1.15	1.4554	1.4117	1.3874	1.3947	1.4024	1.4817	1.2136	1.4029	1.3993	1714	−0.190	−0.111	0.079
	**53**	1.56	1.4531	1.4133	1.3876	1.3940	1.3998	1.4775	1.1901	1.4006	1.4003	1722	−0.196	−0.111	0.084
	**54**	2.12	1.4526	1.4099	1.3902	1.3903	1.4025	1.4677	1.1901	1.3994	1.4034	1842	−0.196	−0.104	0.093

Table S22. Energy of the hydrogen bonding E_{HB} (in kcal mol^{-1}) of MTA [E_{HB}(MTA)] and FBA [E_{HB}(FBA)] methods, [E_{HB}(MTA) – E_{HB}(FBA)] difference for structures from the non-RAHB cluster within the $r_{O\cdots H}$ range of 1.75 – 1.80 Å.

No compounds	$-E_{HB}$(MTA)	$-E_{HB}$(FBA)	E_{HB}(MTA) – E_{HB}(FBA)	$r_{O\cdots H}$
**68b**	4.26	5.67	−1.41	1.7948
**33e**	4.46	5.75	−1.29	1.7897
**24a**	5.21	5.83	−0.62	1.7850
**20**	5.79	5.94	−0.15	1.7784
**88a**	4.84	5.97	−1.13	1.7767
**21**	5.53	6.15	−0.62	1.7669
**57b**	5.82	6.15	−0.33	1.7668
**24b**	5.94	6.36	−0.42	1.7551
**22**	6.40	6.36	0.04	1.7549
**‹$-E_{HB}$(MTA, FBA)›, ‹E_{HB}(MTA) – E_{HB}(FBA)›**	5.36	6.02	−0.66	

Table S23. Energy of the hydrogen bonding E_{HB} (in kcal mol^{-1}) of MTA [E_{HB}(MTA)] and FBA [E_{HB}(FBA)] methods, [E_{HB}(MTA) – E_{HB}(FBA)] difference for structures from the non-RAHB cluster within the $r_{O\cdots H}$ range of 1.70 – 1.75 Å.

No compounds	$-E_{HB}$(MTA)	$-E_{HB}$(FBA)	E_{HB}(MTA) – E_{HB}(FBA)	$r_{O\cdots H}$
**33c**	5.93	6.51	−0.58	1.7469
**37b**	6.92	6.54	0.38	1.7455
**88b**	5.36	6.73	−1.37	1.7350
**37a**	9.28	6.76	2.52	1.7338
**58**	5.91	6.78	−0.87	1.7329
**89**	6.32	6.94	−0.62	1.7243
**‹$-E_{HB}$(MTA, FBA)›, ‹E_{HB}(MTA) – E_{HB}(FBA)›**	6.62	6.71	−0.09	

Table S24. Energy of the hydrogen bonding E_{HB} (in kcal mol^{-1}) of MTA [E_{HB}(MTA)] and FBA [E_{HB}(FBA)] methods, [E_{HB}(MTA) – E_{HB}(FBA)] difference for structures from the non-RAHB cluster within the $r_{O\cdots H}$ range of 1.65 – 1.70 Å.

No compounds	$-E_{HB}$(MTA)	$-E_{HB}$(FBA)	E_{HB}(MTA) – E_{HB}(FBA)	$r_{O\cdots H}$
**90**	8.13	7.77	0.36	1.6842
**81a**	5.25	7.94	−2.69	1.6764
**80**	5.77	8.12	−2.35	1.6682
**81b**	5.66	8.27	−2.61	1.6615
**82a**	6.41	8.44	−2.03	1.6538
**102a**	11.29	8.44	2.85	1.6537
**79**	6.56	8.51	−1.95	1.6509
**‹$-E_{HB}$(MTA, FBA)›, ‹E_{HB}(MTA) – E_{HB}(FBA)›**	7.01	8.21	−1.20	

Table S25. Energy of the hydrogen bonding E_{HB} (in kcal mol^{-1}) of MTA [E_{HB}(MTA)] and FBA [E_{HB}(FBA)] methods, [E_{HB}(MTA) – E_{HB}(FBA)] difference for structures from the non-RAHB cluster within the $r_{O\cdots H}$ range of 1.60 – 1.65 Å.

No compounds	$-E_{HB}$(MTA)	$-E_{HB}$(FBA)	E_{HB}(MTA) – E_{HB}(FBA)	$r_{O\cdots H}$
**94**	9.58	8.78	0.80	1.6390

**82b**	7.01	8.86	−1.85	1.6357
**92**	8.19	8.93	−0.74	1.6327
**35**	11.15	9.06	2.09	1.6274
**85a**	6.32	9.21	−2.89	1.6212
**36**	9.35	9.26	0.09	1.6192
**34**	12.48	9.48	3.00	1.6100
**85b**	7.09	9.57	−2.48	1.6064
**84a**	9.20	9.60	−0.40	1.6053
**102b**	11.76	9.72	2.04	1.6004
**‹−E_{HB}(MTA, FBA)›, ‹E_{HB}(MTA) − E_{HB}(FBA)›**	9.21	9.25	−0.04	

Table S26. Energy of the hydrogen bonding E_{HB} (in kcal mol^{-1}) of MTA [E_{HB}(MTA)] and FBA [E_{HB}(FBA)] methods, [E_{HB}(MTA) – E_{HB}(FBA)] difference for structures from the non-RAHB cluster within the $r_{O\cdots H}$ range of 1.60 – 1.80 Å (total range).

**No compounds**	**−E_{HB}(MTA)**	**−E_{HB}(FBA)**	**E_{HB}(MTA) − E_{HB}(FBA)**	**$r_{O\cdots H}$**
**68b**	4.26	5.67	−1.41	1.7948
**33e**	4.46	5.75	−1.29	1.7897
**24a**	5.21	5.83	−0.62	1.7850
**20**	5.79	5.94	−0.15	1.7784
**88a**	4.84	5.97	−1.13	1.7767
**21**	5.53	6.15	−0.62	1.7669
**57b**	5.82	6.15	−0.33	1.7668
**24b**	5.94	6.36	−0.42	1.7551
**22**	6.40	6.36	0.04	1.7549
**33c**	5.93	6.51	−0.58	1.7469
**37b**	6.92	6.54	0.38	1.7455
**88b**	5.36	6.73	−1.37	1.7350
**37a**	9.28	6.76	2.52	1.7338
**58**	5.91	6.78	−0.87	1.7329
**89**	6.32	6.94	−0.62	1.7243
**90**	8.13	7.77	0.36	1.6842
**81a**	5.25	7.94	−2.69	1.6764
**80**	5.77	8.12	−2.35	1.6682
**81b**	5.66	8.27	−2.61	1.6615
**82a**	6.41	8.44	−2.03	1.6538
**102a**	11.29	8.44	2.85	1.6537
**79**	6.56	8.51	−1.95	1.6509
**94**	9.58	8.78	0.80	1.6390
**82b**	7.01	8.86	−1.85	1.6357
**92**	8.19	8.93	−0.74	1.6327
**35**	11.15	9.06	2.09	1.6274
**85a**	6.32	9.21	−2.89	1.6212
**36**	9.35	9.26	0.09	1.6192
**34**	12.48	9.48	3.00	1.6100
**85b**	7.09	9.57	−2.48	1.6064
**84a**	9.20	9.60	−0.40	1.6053
**102b**	11.76	9.72	2.04	1.6004
**‹−E_{HB}(MTA, FBA)›, ‹E_{HB}(MTA) − E_{HB}(FBA)›**	7.16	7.64	−0.48	

Table S27. Energy of the hydrogen bonding E_{HB} (in kcal mol^{-1}) of MTA [E_{HB}(MTA)] and FBA [E_{HB}(FBA)] methods, [E_{HB}(MTA) – E_{HB}(FBA)] difference for structures from the non-RAHB cluster within the ρ_{BCP} range of 0.030 – 0.035 a.u.

**No compounds**	**−E_{HB}(MTA)**	**−E_{HB}(FBA)**	**E_{HB}(MTA) – E_{HB}(FBA)**	**ρ_{BCP}**
**50**	3.78	3.96	−0.18	0.0300
**67a**	6.01	3.98	2.03	0.0301
**21**	5.53	4.02	1.51	0.0303
**64c**	6.40	4.04	2.36	0.0304
**67b**	6.23	4.12	2.11	0.0308
**23**	3.45	4.31	−0.86	0.0318
**52**	5.90	4.33	1.57	0.0319
**33d**	4.86	4.40	0.46	0.0323
**68b**	4.26	4.42	−0.16	0.0324
**88a**	4.84	4.80	0.04	0.0344
**33a**	5.00	4.86	0.14	0.0347
**33b**	5.31	4.86	0.45	0.0347
**‹−E_{HB}(MTA, FBA)›, ‹E_{HB}(MTA) – E_{HB}(FBA)›**	5.13	4.34	0.79	

Table S28. Energy of the hydrogen bonding E_{HB} (in kcal mol^{-1}) of MTA [E_{HB}(MTA)] and FBA [E_{HB}(FBA)] methods, [E_{HB}(MTA) – E_{HB}(FBA)] difference for structures from the non-RAHB cluster within the ρ_{BCP} range of 0.035 – 0.040 a.u.

**No compounds**	**−E_{HB}(MTA)**	**−E_{HB}(FBA)**	**E_{HB}(MTA) – E_{HB}(FBA)**	**ρ_{BCP}**
**33e**	4.46	5.03	−0.57	0.0356
**57b**	5.82	5.03	0.79	0.0356
**24a**	5.21	5.05	0.16	0.0357
**20**	5.79	5.19	0.60	0.0364
**59**	6.31	5.24	1.07	0.0367
**88b**	5.36	5.47	−0.11	0.0379
**24b**	5.94	5.59	0.35	0.0385
**22**	6.40	5.61	0.79	0.0386
**58**	5.91	5.70	0.21	0.0391
**33c**	5.93	5.76	0.17	0.0394
**37b**	6.92	5.76	1.16	0.0394
**89**	6.32	5.76	0.56	0.0394
**‹−E_{HB}(MTA, FBA)›, ‹E_{HB}(MTA) – E_{HB}(FBA)›**	5.86	5.43	0.43	

Table S29. Energy of the hydrogen bonding E_{HB} (in kcal mol^{-1}) of MTA [E_{HB}(MTA)] and FBA [E_{HB}(FBA)] methods, [E_{HB}(MTA) – E_{HB}(FBA)] difference for structures from the non-RAHB cluster within the ρ_{BCP} range of 0.040 – 0.046 a.u.

**No compounds**	**−E_{HB}(MTA)**	**−E_{HB}(FBA)**	**E_{HB}(MTA) – E_{HB}(FBA)**	**ρ_{BCP}**
**37a**	9.28	6.01	3.27	0.0407
**90**	8.13	6.58	1.55	0.0437
**81a**	5.25	6.64	−1.39	0.0440
**80**	5.77	6.95	−1.18	0.0456
**81b**	5.66	6.99	−1.33	0.0458
**‹−E_{HB}(MTA, FBA)›, ‹E_{HB}(MTA) – E_{HB}(FBA)›**	6.82	6.64	0.18	

Table S30. Energy of the hydrogen bonding E_{HB} (in kcal mol^{-1}) of MTA [E_{HB}(MTA)] and FBA [E_{HB}(FBA)] methods, [E_{HB}(MTA) – E_{HB}(FBA)] difference for structures from the non-RAHB cluster within the ρ_{BCP} range of 0.046 – 0.051 a.u.

No compounds	–E_{HB}(MTA)	–E_{HB}(FBA)	E_{HB}(MTA) – E_{HB}(FBA)	ρ_{BCP}
**82a**	6.41	7.10	−0.69	0.0464
**102a**	11.29	7.10	4.19	0.0464
**79**	6.56	7.27	−0.71	0.0473
**82b**	7.01	7.54	−0.53	0.0487
**92**	8.19	7.69	0.50	0.0495
**94**	9.58	7.77	1.81	0.0499
**85a**	6.32	7.90	−1.58	0.0506
**‹–E_{HB}(MTA, FBA)›, ‹E_{HB}(MTA) – E_{HB}(FBA)›**	7.91	7.49	0.42	

Table S31. Energy of the hydrogen bonding E_{HB} (in kcal mol^{-1}) of MTA [E_{HB}(MTA)] and FBA [E_{HB}(FBA)] methods, [E_{HB}(MTA) – E_{HB}(FBA)] difference for structures from the non-RAHB cluster within the ρ_{BCP} range of 0.030 – 0.051 a.u. (total range).

No compounds	–E_{HB}(MTA)	–E_{HB}(FBA)	E_{HB}(MTA) – E_{HB}(FBA)	ρ_{BCP}
**50**	3.78	3.96	−0.18	0.0300
**67a**	6.01	3.98	2.03	0.0301
**21**	5.53	4.02	1.51	0.0303
**64c**	6.40	4.04	2.36	0.0304
**67b**	6.23	4.12	2.11	0.0308
**23**	3.45	4.31	−0.86	0.0318
**52**	5.90	4.33	1.57	0.0319
**33d**	4.86	4.40	0.46	0.0323
**68b**	4.26	4.42	−0.16	0.0324
**88a**	4.84	4.80	0.04	0.0344
**33a**	5.00	4.86	0.14	0.0347
**33b**	5.31	4.86	0.45	0.0347
**33e**	4.46	5.03	−0.57	0.0356
**57b**	5.82	5.03	0.79	0.0356
**24a**	5.21	5.05	0.16	0.0357
**20**	5.79	5.19	0.60	0.0364
**59**	6.31	5.24	1.07	0.0367
**88b**	5.36	5.47	−0.11	0.0379
**24b**	5.94	5.59	0.35	0.0385
**22**	6.40	5.61	0.79	0.0386
**58**	5.91	5.70	0.21	0.0391
**33c**	5.93	5.76	0.17	0.0394
**37b**	6.92	5.76	1.16	0.0394
**89**	6.32	5.76	0.56	0.0394
**37a**	9.28	6.01	3.27	0.0407
**90**	8.13	6.58	1.55	0.0437
**81a**	5.25	6.64	−1.39	0.0440
**80**	5.77	6.95	−1.18	0.0456
**81b**	5.66	6.99	−1.33	0.0458
**82a**	6.41	7.10	−0.69	0.0464

**102a**	11.29	7.10	4.19	0.0464
**79**	6.56	7.27	−0.71	0.0473
**82b**	7.01	7.54	−0.53	0.0487
**92**	8.19	7.69	0.50	0.0495
**94**	9.58	7.77	1.81	0.0499
**85a**	6.32	7.90	−1.58	0.0506
**‹−E_{HB}(MTA, FBA)›, ‹E_{HB}(MTA) − E_{HB}(FBA)›**	6.15	5.64	0.51	

Table S32. Energy of the hydrogen bonding E_{HB} (in kcal mol^{-1}) of MTA [E_{HB}(MTA)] and FBA [E_{HB}(FBA)] methods, [E_{HB}(MTA) – E_{HB}(FBA)] difference for structures from the non-RAHB cluster within the V_{BCP} range of -0.030 ÷ -0.035 a.u.

No compounds	−E_{HB}(MTA)	−E_{HB}(FBA)	E_{HB}(MTA) − E_{HB}(FBA)	V_{BCP}
**88a**	4.84	5.06	−0.22	−0.0317
**33b**	5.31	5.15	0.16	−0.0322
**57b**	5.82	5.15	0.67	−0.0322
**33a**	5.00	5.18	−0.18	−0.0324
**24a**	5.21	5.36	−0.15	−0.0334
**33e**	4.46	5.36	−0.90	−0.0334
**59**	6.31	5.39	0.92	−0.0336
**20**	5.79	5.48	0.31	−0.0341
**‹−E_{HB}(MTA, FBA)›, ‹E_{HB}(MTA) − E_{HB}(FBA)›**	5.34	5.26	0.08	

Table S33. Energy of the hydrogen bonding E_{HB} (in kcal mol^{-1}) of MTA [E_{HB}(MTA)] and FBA [E_{HB}(FBA)] methods, [E_{HB}(MTA) – E_{HB}(FBA)] difference for structures from the non-RAHB cluster within the V_{BCP} range of -0.035 ÷ -0.040 a.u.

No compounds	−E_{HB}(MTA)	−E_{HB}(FBA)	E_{HB}(MTA) − E_{HB}(FBA)	V_{BCP}
**21**	5.53	5.65	−0.12	−0.0351
**88b**	5.36	5.76	−0.40	−0.0357
**24b**	5.94	5.86	0.08	−0.0363
**58**	5.91	5.89	0.02	−0.0365
**22**	6.40	5.91	0.49	−0.0366
**89**	6.32	5.91	0.41	−0.0366
**33c**	5.93	6.09	−0.16	−0.0376
**37b**	6.92	6.10	0.82	−0.0377
**37a**	9.28	6.28	3.00	−0.0387
**‹−E_{HB}(MTA, FBA)›, ‹E_{HB}(MTA) − E_{HB}(FBA)›**	6.40	5.94	0.46	

Table S34. Energy of the hydrogen bonding E_{HB} (in kcal mol^{-1}) of MTA [E_{HB}(MTA)] and FBA [E_{HB}(FBA)] methods, [E_{HB}(MTA) – E_{HB}(FBA)] difference for structures from the non-RAHB cluster within the V_{BCP} range of -0.040 ÷ -0.046 a.u.

No compounds	−E_{HB}(MTA)	−E_{HB}(FBA)	E_{HB}(MTA) − E_{HB}(FBA)	V_{BCP}
**90**	8.13	6.76	1.37	−0.0415
**81a**	5.25	6.89	−1.64	−0.0422
**80**	5.77	7.15	−1.38	−0.0437
**102a**	11.29	7.18	4.11	−0.0439

**81b**	5.66	7.23	−1.57	−0.0442
**82a**	6.41	7.37	−0.96	−0.0450
**79**	6.56	7.53	−0.97	−0.0459
**‹−E_{HB}(MTA, FBA)›, ‹E_{HB}(MTA) − E_{HB}(FBA)›**	7.01	7.16	−0.15	

Table S35. Energy of the hydrogen bonding E_{HB} (in kcal mol^{-1}) of MTA [E_{HB}(MTA)] and FBA [E_{HB}(FBA)] methods, [E_{HB}(MTA) – E_{HB}(FBA)] difference for structures from the non-RAHB cluster within the V_{BCP} range of -0.046 ÷ -0.053 a.u.

**No compounds**	**−E_{HB}(MTA)**	**−E_{HB}(FBA)**	**E_{HB}(MTA) − E_{HB}(FBA)**	**V_{BCP}**
**82b**	7.01	7.84	−0.83	−0.0477
**92**	8.19	8.03	0.16	−0.0488
**94**	9.58	8.05	1.53	−0.0489
**85a**	6.32	8.31	−1.99	−0.0504
**84a**	9.20	8.68	0.52	−0.0525
**102b**	11.76	8.68	3.08	−0.0525
**85b**	7.09	8.75	−1.66	−0.0529
**‹−E_{HB}(MTA, FBA)›, ‹E_{HB}(MTA) − E_{HB}(FBA)›**	8.45	8.33	0.12	

Table S36. Energy of the hydrogen bonding E_{HB} (in kcal mol^{-1}) of MTA [E_{HB}(MTA)] and FBA [E_{HB}(FBA)] methods, [E_{HB}(MTA) – E_{HB}(FBA)] difference for structures from the non-RAHB cluster within the V_{BCP} range of 0.0300 – 0.053 a.u. (total range).

**No compounds**	**−E_{HB}(MTA)**	**−E_{HB}(FBA)**	**E_{HB}(MTA) − E_{HB}(FBA)**	**V_{BCP}**
**88a**	4.84	5.06	−0.22	−0.0317
**33b**	5.31	5.15	0.16	−0.0322
**57b**	5.82	5.15	0.67	−0.0322
**33a**	5.00	5.18	−0.18	−0.0324
**24a**	5.21	5.36	−0.15	−0.0334
**33e**	4.46	5.36	−0.90	−0.0334
**59**	6.31	5.39	0.92	−0.0336
**20**	5.79	5.48	0.31	−0.0341
**21**	5.53	5.65	−0.12	−0.0351
**88b**	5.36	5.76	−0.40	−0.0357
**24b**	5.94	5.86	0.08	−0.0363
**58**	5.91	5.89	0.02	−0.0365
**22**	6.40	5.91	0.49	−0.0366
**89**	6.32	5.91	0.41	−0.0366
**33c**	5.93	6.09	−0.16	−0.0376
**37b**	6.92	6.10	0.82	−0.0377
**37a**	9.28	6.28	3.00	−0.0387
**90**	8.13	6.76	1.37	−0.0415
**81a**	5.25	6.89	−1.64	−0.0422
**80**	5.77	7.15	−1.38	−0.0437
**102a**	11.29	7.18	4.11	−0.0439
**81b**	5.66	7.23	−1.57	−0.0442
**82a**	6.41	7.37	−0.96	−0.0450
**79**	6.56	7.53	−0.97	−0.0459

**82b**	7.01	7.84	−0.83	−0.0477
**92**	8.19	8.03	0.16	−0.0488
**94**	9.58	8.05	1.53	−0.0489
**85a**	6.32	8.31	−1.99	−0.0504
**84a**	9.20	8.68	0.52	−0.0525
**102b**	11.76	8.68	3.08	−0.0525
**85b**	7.09	8.75	−1.66	−0.0529
**‹−E_{HB}(MTA, FBA)›, ‹E_{HB}(MTA) – E_{HB}(FBA)›**	6.73	6.58	0.15	

Table S37. Energy of the hydrogen bonding E_{HB} (in kcal mol^{-1}) of MTA and FBA methods, π-contribution to the total energy of RAHB interaction for compounds of the RAHB cluster within the $r_{O\cdots H}$ range of 1.75 – 1.80 Å.

No compounds	−E_{HB}(MTA)	−E_{HB}(FBA)	π-contribution	$r_{O\cdots H}$
**46**	11.41	5.68	5.73	1.7941
**106**	15.98	5.71	10.27	1.7919
**138a**	10.59	5.86	4.73	1.7830
**143**	10.38	5.98	4.40	1.7765
**144**	13.83	6.07	7.76	1.7711
**40**	13.73	6.22	7.51	1.7626
**101b**	13.78	6.28	7.50	1.7595
**83a**	11.71	6.31	5.40	1.7576
**34**	15.26	6.44	8.82	1.7504
**‹−E_{HB}(MTA, FBA)›, ‹π-contribution›**	12.96	6.06	6.90	

Table S38. Energy of the hydrogen bonding E_{HB} (in kcal mol^{-1}) of MTA and FBA methods, π-contribution to the total energy of RAHB interaction for compounds of the RAHB cluster within the $r_{O\cdots H}$ range of 1.70 – 1.75 Å

No compounds	−E_{HB}(MTA)	−E_{HB}(FBA)	π-contribution	$r_{O\cdots H}$
**35**	11.90	6.50	5.40	1.7475
**141**	12.93	6.55	6.38	1.7449
**54**	12.63	6.62	6.01	1.7411
**94b**	11.56	6.63	4.93	1.7404
**94a**	11.88	6.73	5.15	1.7350
**109**	11.81	6.78	5.03	1.7326
**50**	14.53	6.80	7.73	1.7318
**57**	12.61	6.91	5.70	1.7260
**90a**	11.72	6.97	4.75	1.7230
**120**	14.15	6.98	7.17	1.7226
**92b**	11.75	6.98	4.77	1.7224
**83b**	12.97	7.01	5.96	1.7211
**92a**	11.82	7.02	4.80	1.7205
**44**	13.89	7.02	6.87	1.7202
**138b**	12.40	7.04	5.36	1.7194
**33**	15.76	7.05	8.71	1.7191
**179**	13.92	7.05	6.87	1.7190
**145**	15.72	7.05	8.67	1.7189

**91a**	11.83	7.05	4.78	1.7188
**72**	13.41	7.07	6.34	1.7177
**94e**	11.93	7.18	4.75	1.7125
**139a**	11.60	7.22	4.38	1.7102
**48**	13.66	7.24	6.42	1.7093
**56**	13.54	7.43	6.11	1.7002
**‹–E_{HB}(MTA, FBA)›, ‹π-contribution›**	12.91	6.95	5.96	

Table S39. Energy of the hydrogen bonding E_{HB} (in kcal mol^{-1}) of MTA and FBA methods, π-contribution to the total energy of RAHB interaction for compounds of the RAHB cluster within the $r_{O\cdots H}$ range of 1.65 – 1.70 Å

No compounds	–E_{HB}(MTA)	–E_{HB}(FBA)	π-contribution	$r_{O\cdots H}$
**53**	13.81	7.46	6.35	1.6989
**146**	15.60	7.50	8.10	1.6966
**185**	12.91	7.60	5.31	1.6921
**60**	14.12	7.66	6.46	1.6892
**135**	14.44	7.68	6.76	1.6884
**1**	14.51	7.69	6.82	1.6880
**93a**	11.88	7.69	4.19	1.6879
**96a**	11.71	7.69	4.02	1.6879
**95b**	15.42	7.69	7.73	1.6878
**177**	13.47	7.72	5.75	1.6862
**4**	14.03	7.74	6.29	1.6854
**41**	14.35	7.75	6.60	1.6849
**73**	13.26	7.75	5.51	1.6848
**64**	14.36	7.82	6.54	1.6817
**110b**	13.18	7.83	5.35	1.6812
**29a**	13.95	7.90	6.05	1.6781
**100**	11.62	7.93	3.69	1.6766
**123**	14.60	7.96	6.64	1.6755
**118b**	14.32	7.96	6.36	1.6753
**117b**	14.28	8.00	6.28	1.6735
**111a**	14.46	8.03	6.43	1.6721
**129**	14.23	8.08	6.15	1.6700
**62**	14.30	8.11	6.19	1.6686
**3**	14.57	8.11	6.46	1.6684
**117a**	14.50	8.14	6.36	1.6670
**94d**	14.75	8.19	6.56	1.6647
**66**	14.31	8.21	6.10	1.6641
**21**	14.52	8.21	6.31	1.6639
**142**	12.59	8.22	4.37	1.6635
**186**	11.69	8.24	3.45	1.6625
**127**	15.52	8.25	7.27	1.6622
**88c**	13.76	8.28	5.48	1.6607
**86b**	14.07	8.29	5.78	1.6604
**94c**	15.12	8.30	6.82	1.6599
**85a**	14.80	8.37	6.43	1.6568

**92c**	14.54	8.41	6.13	1.6551
**84c**	15.15	8.42	6.73	1.6549
**75**	13.75	8.44	5.31	1.6539
**148**	13.72	8.44	5.28	1.6538
**180**	13.84	8.49	5.35	1.6518
**136**	14.97	8.50	6.47	1.6513
**111b**	16.25	8.50	7.75	1.6512
**29b**	15.35	8.51	6.84	1.6510
**2**	15.10	8.51	6.59	1.6507
**61**	14.66	8.52	6.14	1.6502
**‹–E_{HB}(MTA, FBA)›, ‹π-contribution›**	14.14	8.06	6.08	

Table S40. Energy of the hydrogen bonding E_{HB} (in kcal mol^{-1}) of MTA and FBA methods, π-contribution to the total energy of RAHB interaction for compounds of the RAHB cluster within the $r_{O\cdots H}$ range of 1.60 – 1.65 Å.

**No comp**	**–E_{HB}(MTA)**	**–E_{HB}(FBA)**	**π-contribution**	**$r_{O\cdots H}$**
**31**	14.08	8.54	5.54	1.6496
**70**	14.32	8.59	5.73	1.6473
**90b**	14.96	8.60	6.36	1.6471
**137**	14.08	8.62	5.46	1.6461
**20**	15.22	8.64	6.58	1.6451
**122**	14.73	8.66	6.07	1.6443
**140**	12.01	8.66	3.35	1.6442
**152**	14.16	8.67	5.49	1.6440
**147**	16.28	8.67	7.61	1.6438
**91b**	15.03	8.68	6.35	1.6436
**92d**	15.12	8.69	6.43	1.6429
**96b**	12.83	8.73	4.10	1.6412
**68**	14.61	8.74	5.87	1.6410
**139b**	13.33	8.76	4.57	1.6401
**63**	14.80	8.77	6.03	1.6395
**6**	15.17	8.77	6.40	1.6394
**112a**	14.61	8.78	5.83	1.6392
**162**	16.03	8.78	7.25	1.6391
**110a**	16.89	8.79	8.10	1.6386
**121**	14.79	8.80	5.99	1.6383
**5**	15.16	8.88	6.28	1.6351
**153**	14.15	8.92	5.23	1.6333
**94f**	15.38	8.94	6.44	1.6324
**183**	13.78	8.95	4.83	1.6318
**112b**	16.41	8.97	7.44	1.6313
**65**	14.99	9.01	5.98	1.6294
**11**	15.46	9.01	6.45	1.6293
**67**	15.22	9.07	6.15	1.6271
**161**	16.09	9.10	6.99	1.6257
**22**	15.29	9.11	6.18	1.6253
**71**	16.03	9.12	6.91	1.6249

**93b**	15.23	9.14	6.09	1.6242
**87a**	15.75	9.15	6.60	1.6237
**12**	15.63	9.18	6.45	1.6225
**84a**	16.10	9.18	6.92	1.6225
**167**	14.33	9.21	5.12	1.6212
**32**	16.22	9.22	7.00	1.6206
**9b**	15.44	9.26	6.18	1.6193
**150**	15.20	9.28	5.92	1.6185
**69**	15.76	9.30	6.46	1.6173
**9a**	15.95	9.32	6.63	1.6167
**14**	15.24	9.35	5.89	1.6156
**7**	15.09	9.39	5.70	1.6139
**26**	15.26	9.39	5.87	1.6138
**84d**	17.01	9.45	7.56	1.6114
**25**	15.91	9.46	6.45	1.6110
**59**	16.29	9.46	6.83	1.6110
**108b**	16.60	9.47	7.13	1.6104
**151**	15.13	9.49	5.64	1.6099
**165**	15.96	9.50	6.46	1.6092
**13a**	15.32	9.55	5.77	1.6073
**45**	15.90	9.55	6.35	1.6073
**182**	13.54	9.62	3.92	1.6047
**16**	16.38	9.64	6.74	1.6036
**102**	15.15	9.66	5.49	1.6030
**154**	15.33	9.66	5.67	1.6030
**181**	14.65	9.67	4.98	1.6027
**149**	17.44	9.72	7.72	1.6006
**88a**	17.13	9.73	7.40	1.6003
**‹$-E_{HB}$(MTA, FBA)›, ‹π-contribution›**	15.25	9.10	6.15	

Table S41. Energy of the hydrogen bonding E_{HB} (in kcal mol^{-1}) of MTA and FBA methods, π-contribution to the total energy of RAHB interaction for compounds of the RAHB cluster within the $r_{O\cdots H}$ range of 1.60 – 1.80 Å (total range).

**No compounds**	**$-E_{HB}$(MTA)**	**$-E_{HB}$(FBA)**	**π-contribution**	**$r_{O\cdots H}$**
**46**	11.41	5.68	5.73	1.7941
**106**	15.98	5.71	10.27	1.7919
**138a**	10.59	5.86	4.73	1.7830
**143**	10.38	5.98	4.40	1.7765
**144**	13.83	6.07	7.76	1.7711
**40**	13.73	6.22	7.51	1.7626
**101b**	13.78	6.28	7.50	1.7595
**83a**	11.71	6.31	5.40	1.7576
**34**	15.26	6.44	8.82	1.7504
**35**	11.90	6.50	5.40	1.7475
**141**	12.93	6.55	6.38	1.7449
**54**	12.63	6.62	6.01	1.7411
**94b**	11.56	6.63	4.93	1.7404

**94a**	11.88	6.73	5.15	1.7350
**109**	11.81	6.78	5.03	1.7326
**50**	14.53	6.80	7.73	1.7318
**57**	12.61	6.91	5.70	1.7260
**90a**	11.72	6.97	4.75	1.7230
**120**	14.15	6.98	7.17	1.7226
**92b**	11.75	6.98	4.77	1.7224
**83b**	12.97	7.01	5.96	1.7211
**92a**	11.82	7.02	4.80	1.7205
**44**	13.89	7.02	6.87	1.7202
**138b**	12.40	7.04	5.36	1.7194
**33**	15.76	7.05	8.71	1.7191
**179**	13.92	7.05	6.87	1.7190
**145**	15.72	7.05	8.67	1.7189
**91a**	11.83	7.05	4.78	1.7188
**72**	13.41	7.07	6.34	1.7177
**94e**	11.93	7.18	4.75	1.7125
**139a**	11.60	7.22	4.38	1.7102
**48**	13.66	7.24	6.42	1.7093
**56**	13.54	7.43	6.11	1.7002
**53**	13.81	7.46	6.35	1.6989
**146**	15.60	7.50	8.10	1.6966
**185**	12.91	7.60	5.31	1.6921
**60**	14.12	7.66	6.46	1.6892
**135**	14.44	7.68	6.76	1.6884
**1**	14.51	7.69	6.82	1.6880
**93a**	11.88	7.69	4.19	1.6879
**96a**	11.71	7.69	4.02	1.6879
**95b**	15.42	7.69	7.73	1.6878
**177**	13.47	7.72	5.75	1.6862
**4**	14.03	7.74	6.29	1.6854
**41**	14.35	7.75	6.60	1.6849
**73**	13.26	7.75	5.51	1.6848
**64**	14.36	7.82	6.54	1.6817
**110b**	13.18	7.83	5.35	1.6812
**29a**	13.95	7.90	6.05	1.6781
**100**	11.62	7.93	3.69	1.6766
**123**	14.60	7.96	6.64	1.6755
**118b**	14.32	7.96	6.36	1.6753
**117b**	14.28	8.00	6.28	1.6735
**111a**	14.46	8.03	6.43	1.6721
**129**	14.23	8.08	6.15	1.6700
**62**	14.30	8.11	6.19	1.6686
**3**	14.57	8.11	6.46	1.6684
**117a**	14.50	8.14	6.36	1.6670
**94d**	14.75	8.19	6.56	1.6647
**66**	14.31	8.21	6.10	1.6641
**21**	14.52	8.21	6.31	1.6639

**142**	12.59	8.22	4.37	1.6635
**186**	11.69	8.24	3.45	1.6625
**127**	15.52	8.25	7.27	1.6622
**88c**	13.76	8.28	5.48	1.6607
**86b**	14.07	8.29	5.78	1.6604
**94c**	15.12	8.30	6.82	1.6599
**85a**	14.80	8.37	6.43	1.6568
**92c**	14.54	8.41	6.13	1.6551
**84c**	15.15	8.42	6.73	1.6549
**75**	13.75	8.44	5.31	1.6539
**148**	13.72	8.44	5.28	1.6538
**180**	13.84	8.49	5.35	1.6518
**136**	14.97	8.50	6.47	1.6513
**111b**	16.25	8.50	7.75	1.6512
**29b**	15.35	8.51	6.84	1.6510
**2**	15.10	8.51	6.59	1.6507
**61**	14.66	8.52	6.14	1.6502
**31**	14.08	8.54	5.54	1.6496
**70**	14.32	8.59	5.73	1.6473
**90b**	14.96	8.60	6.36	1.6471
**137**	14.08	8.62	5.46	1.6461
**20**	15.22	8.64	6.58	1.6451
**122**	14.73	8.66	6.07	1.6443
**140**	12.01	8.66	3.35	1.6442
**152**	14.16	8.67	5.49	1.6440
**147**	16.28	8.67	7.61	1.6438
**91b**	15.03	8.68	6.35	1.6436
**92d**	15.12	8.69	6.43	1.6429
**96b**	12.83	8.73	4.10	1.6412
**68**	14.61	8.74	5.87	1.6410
**139b**	13.33	8.76	4.57	1.6401
**63**	14.80	8.77	6.03	1.6395
**6**	15.17	8.77	6.40	1.6394
**112a**	14.61	8.78	5.83	1.6392
**162**	16.03	8.78	7.25	1.6391
**110a**	16.89	8.79	8.10	1.6386
**121**	14.79	8.80	5.99	1.6383
**5**	15.16	8.88	6.28	1.6351
**153**	14.15	8.92	5.23	1.6333
**94f**	15.38	8.94	6.44	1.6324
**183**	13.78	8.95	4.83	1.6318
**112b**	16.41	8.97	7.44	1.6313
**65**	14.99	9.01	5.98	1.6294
**11**	15.46	9.01	6.45	1.6293
**67**	15.22	9.07	6.15	1.6271
**161**	16.09	9.10	6.99	1.6257
**22**	15.29	9.11	6.18	1.6253
**71**	16.03	9.12	6.91	1.6249

**93b**	15.23	9.14	6.09	1.6242
**87a**	15.75	9.15	6.60	1.6237
**12**	15.63	9.18	6.45	1.6225
**84a**	16.10	9.18	6.92	1.6225
**167**	14.33	9.21	5.12	1.6212
**32**	16.22	9.22	7.00	1.6206
**9b**	15.44	9.26	6.18	1.6193
**150**	15.20	9.28	5.92	1.6185
**69**	15.76	9.30	6.46	1.6173
**9a**	15.95	9.32	6.63	1.6167
**14**	15.24	9.35	5.89	1.6156
**7**	15.09	9.39	5.70	1.6139
**26**	15.26	9.39	5.87	1.6138
**84d**	17.01	9.45	7.56	1.6114
**25**	15.91	9.46	6.45	1.6110
**59**	16.29	9.46	6.83	1.6110
**108b**	16.60	9.47	7.13	1.6104
**151**	15.13	9.49	5.64	1.6099
**165**	15.96	9.50	6.46	1.6092
**13a**	15.32	9.55	5.77	1.6073
**45**	15.90	9.55	6.35	1.6073
**182**	13.54	9.62	3.92	1.6047
**16**	16.38	9.64	6.74	1.6036
**102**	15.15	9.66	5.49	1.6030
**154**	15.33	9.66	5.67	1.6030
**181**	14.65	9.67	4.98	1.6027
**149**	17.44	9.72	7.72	1.6006
**88a**	17.13	9.73	7.40	1.6003
**‹−E_{HB}(MTA, FBA)›, ‹π-contribution›**	14.33	8.18	6.15	

Table S42. Energy of the hydrogen bonding E_{HB} (in kcal mol^{-1}) of MTA and FBA methods, π-contribution to the total energy of RAHB interaction for compounds of the RAHB cluster within the ρ_{BCP} range of 0.030 – 0.036 a.u.

**No compounds**	**−E_{HB}(MTA)**	**−E_{HB}(FBA)**	**π-contribution**	**ρ_{BCP}**
**51**	11.39	4.02	7.37	0.0303
**42**	11.28	4.54	6.74	0.0330
**101a**	15.14	4.57	10.57	0.0332
**95a**	12.59	4.69	7.90	0.0338
**38**	11.08	4.71	6.37	0.0339
**46**	11.41	5.00	6.41	0.0354
**106**	15.98	5.09	10.89	0.0359
**‹−E_{HB}(MTA, FBA)›, ‹π-contribution›**	12.70	4.66	8.04	

Table S43. Energy of the hydrogen bonding E_{HB} (in kcal mol^{-1}) of MTA and FBA methods, π-contribution to the total energy of RAHB interaction for compounds of the RAHB cluster within the ρ_{BCP} range of 0.036 – 0.041 a.u.

No compounds	$-E_{HB}$(MTA)	$-E_{HB}$(FBA)	π-contribution	ρ_{BCP}
**138a**	10.59	5.19	5.40	0.0364
**143**	10.38	5.28	5.10	0.0369
**144**	13.83	5.45	8.38	0.0378
**40**	13.73	5.57	8.16	0.0384
**83a**	11.71	5.61	6.10	0.0386
**101b**	13.78	5.61	8.17	0.0386
**141**	12.93	5.76	7.17	0.0394
**35**	11.90	5.80	6.10	0.0396
**34**	15.26	5.82	9.44	0.0397
**54**	12.63	5.90	6.73	0.0401
**94b**	11.56	5.91	5.65	0.0402
**94a**	11.88	6.01	5.87	0.0407
**‹$-E_{HB}$(MTA, FBA)›, ‹π-contribution›**	12.52	5.66	6.86	

Table S44. Energy of the hydrogen bonding E_{HB} (in kcal mol^{-1}) of MTA and FBA methods, π-contribution to the total energy of RAHB interaction for the RAHB structures within the ρ_{BCP} range of 0.041 – 0.046 a.u.

No compounds	$-E_{HB}$(MTA)	$-E_{HB}$(FBA)	π-contribution	ρ_{BCP}
**109**	11.81	6.07	5.74	0.0410
**50**	14.53	6.09	8.44	0.0411
**57**	12.61	6.16	6.45	0.0415
**90a**	11.72	6.24	5.48	0.0419
**92b**	11.75	6.26	5.49	0.0420
**179**	13.92	6.26	7.66	0.0420
**83b**	12.97	6.28	6.69	0.0421
**92a**	11.82	6.28	5.54	0.0421
**120**	14.15	6.28	7.87	0.0421
**138b**	12.40	6.30	6.10	0.0422
**72**	13.41	6.32	7.09	0.0423
**44**	13.89	6.34	7.55	0.0424
**145**	15.72	6.35	9.37	0.0425
**91a**	11.83	6.37	5.46	0.0426
**33**	15.76	6.41	9.35	0.0428
**94e**	11.93	6.43	5.50	0.0429
**139a**	11.60	6.49	5.11	0.0432
**48**	13.66	6.53	7.13	0.0434
**56**	13.54	6.66	6.88	0.0441
**53**	13.81	6.70	7.11	0.0443
**93a**	11.88	6.74	5.14	0.0445
**185**	12.91	6.78	6.13	0.0447
**146**	15.60	6.87	8.73	0.0452
**60**	14.12	6.91	7.21	0.0454
**177**	13.47	6.91	6.56	0.0454
**96a**	11.71	6.95	4.76	0.0456
**135**	14.44	6.97	7.47	0.0457
**1**	14.51	6.99	7.52	0.0458
**95b**	15.42	7.01	8.41	0.0459
**‹$-E_{HB}$(MTA, FBA)›, ‹π-contribution›**	13.34	6.51	6.83	

Table S45. Energy of the hydrogen bonding E_{HB} (in kcal mol^{-1}) of MTA and FBA methods, π-contribution to the total energy of RAHB interaction for compounds of the RAHB cluster within the ρ_{BCP} range of 0.046 – 0.051 a.u.

**No compounds**	**$-E_{HB}$(MTA)**	**$-E_{HB}$(FBA)**	**π-contribution**	**ρ_{BCP}**
**4**	14.03	7.02	7.01	0.0460
**73**	13.26	7.04	6.22	0.0461
**41**	14.35	7.06	7.29	0.0462
**64**	14.36	7.06	7.30	0.0462
**110b**	13.18	7.08	6.10	0.0463
**29a**	13.95	7.20	6.75	0.0469
**100**	11.62	7.23	4.39	0.0471
**117b**	14.28	7.25	7.03	0.0472
**118b**	14.32	7.25	7.07	0.0472
**123**	14.60	7.25	7.35	0.0472
**111a**	14.46	7.33	7.13	0.0476
**62**	14.30	7.35	6.95	0.0477
**129**	14.23	7.35	6.88	0.0477
**186**	11.69	7.39	4.30	0.0479
**3**	14.57	7.43	7.14	0.0481
**117a**	14.50	7.43	7.07	0.0481
**66**	14.31	7.45	6.86	0.0482
**94d**	14.75	7.46	7.29	0.0483
**21**	14.52	7.50	7.02	0.0485
**86b**	14.07	7.54	6.53	0.0487
**88c**	13.76	7.56	6.20	0.0488
**127**	15.52	7.56	7.96	0.0488
**142**	12.59	7.56	5.03	0.0488
**94c**	15.12	7.60	7.52	0.0490
**85a**	14.80	7.66	7.14	0.0493
**180**	13.84	7.66	6.18	0.0493
**84c**	15.15	7.68	7.47	0.0494
**92c**	14.54	7.71	6.83	0.0496
**75**	13.75	7.73	6.02	0.0497
**148**	13.72	7.73	5.99	0.0497
**2**	15.10	7.81	7.29	0.0501
**29b**	15.35	7.81	7.54	0.0501
**111b**	16.25	7.81	8.44	0.0501
**136**	14.97	7.81	7.16	0.0501
**31**	14.08	7.83	6.25	0.0502
**61**	14.66	7.83	6.83	0.0502
**70**	14.32	7.89	6.43	0.0505
**140**	12.01	7.89	4.12	0.0505
**90b**	14.96	7.90	7.06	0.0506
**137**	14.08	7.90	6.18	0.0506
**20**	15.22	7.94	7.28	0.0508
**‹$-E_{HB}$(MTA, FBA)›, ‹π-contribution›**	14.22	7.52	6.70	

Table S46. Energy of the hydrogen bonding E_{HB} (in kcal mol^{-1}) of MTA and FBA methods, π-contribution to the total energy of RAHB interaction for compounds of the RAHB cluster within the ρ_{BCP} range of 0.030 – 0.051 a.u. (total range).

No compounds	$-E_{HB}$(MTA)	$-E_{HB}$(FBA)	π-contribution	ρ_{BCP}
**51**	11.39	4.02	7.37	0.0303
**42**	11.28	4.54	6.74	0.033
**101a**	15.14	4.57	10.57	0.0332
**95a**	12.59	4.69	7.90	0.0338
**38**	11.08	4.71	6.37	0.0339
**46**	11.41	5.00	6.41	0.0354
**106**	15.98	5.09	10.89	0.0359
**138a**	10.59	5.19	5.40	0.0364
**143**	10.38	5.28	5.10	0.0369
**144**	13.83	5.45	8.38	0.0378
**40**	13.73	5.57	8.16	0.0384
**83a**	11.71	5.61	6.10	0.0386
**101b**	13.78	5.61	8.17	0.0386
**141**	12.93	5.76	7.17	0.0394
**35**	11.9	5.80	6.10	0.0396
**34**	15.26	5.82	9.44	0.0397
**54**	12.63	5.90	6.73	0.0401
**94b**	11.56	5.91	5.65	0.0402
**94a**	11.88	6.01	5.87	0.0407
**109**	11.81	6.07	5.74	0.0410
**50**	14.53	6.09	8.44	0.0411
**57**	12.61	6.16	6.45	0.0415
**90a**	11.72	6.24	5.48	0.0419
**92b**	11.75	6.26	5.49	0.0420
**179**	13.92	6.26	7.66	0.0420
**83b**	12.97	6.28	6.69	0.0421
**92a**	11.82	6.28	5.54	0.0421
**120**	14.15	6.28	7.87	0.0421
**138b**	12.4	6.30	6.10	0.0422
**72**	13.41	6.32	7.09	0.0423
**44**	13.89	6.34	7.55	0.0424
**145**	15.72	6.35	9.37	0.0425
**91a**	11.83	6.37	5.46	0.0426
**33**	15.76	6.41	9.35	0.0428
**94e**	11.93	6.43	5.50	0.0429
**139a**	11.6	6.49	5.11	0.0432
**48**	13.66	6.53	7.13	0.0434
**56**	13.54	6.66	6.88	0.0441
**53**	13.81	6.70	7.11	0.0443
**93a**	11.88	6.74	5.14	0.0445
**185**	12.91	6.78	6.13	0.0447
**146**	15.6	6.87	8.73	0.0452
**60**	14.12	6.91	7.21	0.0454
**177**	13.47	6.91	6.56	0.0454

**96a**	11.71	6.95	4.76	0.0456
**135**	14.44	6.97	7.47	0.0457
**1**	14.51	6.99	7.52	0.0458
**95b**	15.42	7.01	8.41	0.0459
**4**	14.03	7.02	7.01	0.0460
**73**	13.26	7.04	6.22	0.0461
**41**	14.35	7.06	7.29	0.0462
**64**	14.36	7.06	7.30	0.0462
**110b**	13.18	7.08	6.10	0.0463
**29a**	13.95	7.20	6.75	0.0469
**100**	11.62	7.23	4.39	0.0471
**117b**	14.28	7.25	7.03	0.0472
**118b**	14.32	7.25	7.07	0.0472
**123**	14.6	7.25	7.35	0.0472
**111a**	14.46	7.33	7.13	0.0476
**62**	14.3	7.35	6.95	0.0477
**129**	14.23	7.35	6.88	0.0477
**186**	11.69	7.39	4.30	0.0479
**3**	14.57	7.43	7.14	0.0481
**117a**	14.5	7.43	7.07	0.0481
**66**	14.31	7.45	6.86	0.0482
**94d**	14.75	7.46	7.29	0.0483
**21**	14.52	7.50	7.02	0.0485
**86b**	14.07	7.54	6.53	0.0487
**88c**	13.76	7.56	6.20	0.0488
**127**	15.52	7.56	7.96	0.0488
**142**	12.59	7.56	5.03	0.0488
**94c**	15.12	7.60	7.52	0.049
**85a**	14.8	7.66	7.14	0.0493
**180**	13.84	7.66	6.18	0.0493
**84c**	15.15	7.68	7.47	0.0494
**92c**	14.54	7.71	6.83	0.0496
**75**	13.75	7.73	6.02	0.0497
**148**	13.72	7.73	5.99	0.0497
**2**	15.1	7.81	7.29	0.0501
**29b**	15.35	7.81	7.54	0.0501
**111b**	16.25	7.81	8.44	0.0501
**136**	14.97	7.81	7.16	0.0501
**31**	14.08	7.83	6.25	0.0502
**61**	14.66	7.83	6.83	0.0502
**70**	14.32	7.89	6.43	0.0505
**140**	12.01	7.89	4.12	0.0505
**90b**	14.96	7.90	7.06	0.0506
**137**	14.08	7.90	6.18	0.0506
**20**	15.22	7.94	7.28	0.0508
**‹–E_{HB}(MTA, FBA)›, ‹π-contribution›**	13.59	6.72	6.87	

Table S47. Energy of the hydrogen bonding E_{HB} (in kcal mol^{-1}) of MTA and FBA methods, π-contribution to the total energy of RAHB interaction for compounds of the RAHB cluster within the V_{BCP} range of –0.030 ÷ –0.036 a.u.

No compounds	$-E_{HB}$(MTA)	$-E_{HB}$(FBA)	π-contribution	V_{BCP}
**42**	11.28	4.82	6.46	–0.0303
**95a**	12.59	4.92	7.67	–0.0309
**38**	11.08	4.99	6.09	–0.0313
**106**	15.98	5.16	10.82	–0.0323
**46**	11.41	5.30	6.11	–0.0331
**138a**	10.59	5.51	5.08	–0.0343
**143**	10.38	5.55	4.83	–0.0345
**144**	13.83	5.65	8.18	–0.0351
**‹$-E_{HB}$(MTA, FBA)›, ‹π-contribution›**	12.14	5.24	6.90	

Table S48. Energy of the hydrogen bonding E_{HB} (in kcal mol^{-1}) of MTA and FBA methods, π-contribution to the total energy of RAHB interaction for the compounds of the RAHB cluster within the V_{BCP} range of –0.036 ÷ –0.041 a.u.

No compounds	$-E_{HB}$(MTA)	$-E_{HB}$(FBA)	π-contribution	V_{BCP}
**101b**	13.78	5.81	7.97	–0.0360
**40**	13.73	5.86	7.87	–0.0363
**83a**	11.71	5.91	5.80	–0.0366
**34**	15.26	6.05	9.21	–0.0374
**35**	11.90	6.09	5.81	–0.0376
**141**	12.93	6.10	6.83	–0.0377
**94b**	11.56	6.26	5.30	–0.0386
**54**	12.63	6.28	6.35	–0.0387
**50**	14.53	6.35	8.18	–0.0391
**94a**	11.88	6.35	5.53	–0.0391
**109**	11.81	6.35	5.46	–0.0391
**57**	12.61	6.59	6.02	–0.0405
**90a**	11.72	6.59	5.13	–0.0405
**145**	15.72	6.59	9.13	–0.0405
**83b**	12.97	6.61	6.36	–0.0406
**92b**	11.75	6.61	5.14	–0.0406
**92a**	11.82	6.62	5.20	–0.0407
**120**	14.15	6.62	7.53	–0.0407
**92c**	14.54	6.64	7.90	–0.0408
**179**	13.92	6.66	7.26	–0.0409
**‹$-E_{HB}$(MTA, FBA)›, ‹π-contribution›**	13.05	6.35	6.70	

Table S49. Energy of the hydrogen bonding E_{HB} (in kcal mol^{-1}) of MTA and FBA methods, π-contribution to the total energy of RAHB interaction for compounds of the RAHB cluster within the V_{BCP} range of –0.041 ÷ –0.046 a.u.

No compounds	$-E_{HB}$(MTA)	$-E_{HB}$(FBA)	π-contribution	V_{BCP}
**33**	15.76	6.68	9.08	–0.0410
**91a**	11.83	6.68	5.15	–0.0410

**44**	13.89	6.69	7.20	–0.0411
**72**	13.41	6.71	6.70	–0.0412
**138b**	12.40	6.73	5.67	–0.0413
**94e**	11.93	6.76	5.17	–0.0415
**48**	13.66	6.90	6.76	–0.0423
**139a**	11.60	6.92	4.68	–0.0424
**56**	13.54	7.01	6.53	–0.0429
**53**	13.81	7.04	6.77	–0.0431
**93a**	11.88	7.11	4.77	–0.0435
**146**	15.60	7.11	8.49	–0.0435
**185**	12.91	7.20	5.71	–0.0440
**177**	13.47	7.30	6.17	–0.0446
**60**	14.12	7.34	6.78	–0.0448
**135**	14.44	7.34	7.10	–0.0448
**1**	14.51	7.35	7.16	–0.0449
**95b**	15.42	7.35	8.07	–0.0449
**73**	13.26	7.39	5.87	–0.0451
**96a**	11.71	7.41	4.30	–0.0452
**4**	14.03	7.46	6.57	–0.0455
**41**	14.35	7.46	6.89	–0.0455
**110b**	13.18	7.48	5.70	–0.0456
**64**	14.36	7.51	6.85	–0.0458
**‹–E_{HB}(MTA, FBA)›, ‹π-contribution›**	13.54	7.12	6.42	

Table S50. Energy of the hydrogen bonding E_{HB} (in kcal mol^{-1}) of MTA and FBA methods, π-contribution to the total energy of RAHB interaction for compounds of the RAHB cluster within the V_{BCP} range of –0.046 ÷ –0.051 a.u.

**No compounds**	**–E_{HB}(MTA)**	**–E_{HB}(FBA)**	**π-contribution**	**V_{BCP}**
**29a**	13.95	7.60	6.35	–0.0463
**123**	14.60	7.67	6.93	–0.0467
**117b**	14.28	7.68	6.60	–0.0468
**118b**	14.32	7.68	6.64	–0.0468
**111a**	14.46	7.70	6.76	–0.0469
**129**	14.23	7.75	6.48	–0.0472
**100**	11.62	7.77	3.85	–0.0473
**3**	14.57	7.82	6.75	–0.0476
**62**	14.30	7.82	6.48	–0.0476
**117a**	14.50	7.88	6.62	–0.0479
**94d**	14.75	7.91	6.84	–0.0481
**186**	11.69	7.91	3.78	–0.0481
**66**	14.31	7.93	6.38	–0.0482
**86b**	14.07	7.93	6.14	–0.0482
**21**	14.52	7.95	6.57	–0.0483
**88c**	13.76	7.95	5.81	–0.0483
**127**	15.52	7.96	7.56	–0.0484
**85a**	14.80	8.02	6.78	–0.0487

**94c**	15.12	8.03	7.09	–0.0488
**84c**	15.15	8.05	7.10	–0.0489
**142**	12.59	8.12	4.47	–0.0493
**180**	13.84	8.12	5.72	–0.0493
**75**	13.75	8.14	5.61	–0.0494
**111b**	16.25	8.22	8.03	–0.0499
**148**	13.72	8.22	5.50	–0.0499
**29b**	15.35	8.24	7.11	–0.0500
**136**	14.97	8.24	6.73	–0.0500
**2**	15.10	8.26	6.84	–0.0501
**61**	14.66	8.28	6.38	–0.0502
**31**	14.08	8.33	5.75	–0.0505
**70**	14.32	8.35	5.97	–0.0506
**90b**	14.96	8.36	6.60	–0.0507
**137**	14.08	8.36	5.72	–0.0507
**147**	16.28	8.36	7.92	–0.0507
**‹–E_{HB}(MTA, FBA)›, ‹π-contribution›**	14.37	8.02	6.35	

Table S51. Energy of the hydrogen bonding E_{HB} (in kcal mol^{-1}) of MTA and FBA methods, π-contribution to the total energy of RAHB interaction for compounds of the RAHB cluster within the V_{BCP} range of –0.030 ÷ –0.036 a.u. (total range).

**No compounds**	**–E_{HB}(MTA)**	**–E_{HB}(FBA)**	**π-contribution**	**V_{BCP}**
**42**	11.28	4.82	6.46	–0.0303
**95a**	12.59	5.29	7.30	–0.033
**38**	11.08	5.32	5.76	–0.0332
**106**	15.98	5.43	10.55	–0.0338
**46**	11.41	5.44	5.97	–0.0339
**138a**	10.59	5.70	4.89	–0.0354
**143**	10.38	5.79	4.59	–0.0359
**144**	13.83	5.88	7.95	–0.0364
**101b**	13.78	5.96	7.82	–0.0369
**40**	13.73	6.12	7.61	–0.0378
**83a**	11.71	6.22	5.49	–0.0384
**34**	15.26	6.26	9.00	–0.0386
**35**	11.90	6.26	5.64	–0.0386
**141**	12.93	6.40	6.53	–0.0394
**94b**	11.56	6.43	5.13	–0.0396
**54**	12.63	6.45	6.18	–0.0397
**50**	14.53	6.52	8.01	–0.0401
**94a**	11.88	6.54	5.34	–0.0402
**109**	11.81	6.62	5.19	–0.0407
**57**	12.61	6.68	5.93	–0.041
**90a**	11.72	6.69	5.03	–0.0411
**145**	15.72	6.76	8.96	–0.0415
**83b**	12.97	6.83	6.14	–0.0419
**92b**	11.75	6.85	4.90	–0.0420
**92a**	11.82	6.85	4.97	–0.0420

**120**	14.15	6.87	7.28	–0.0421
**92c**	14.54	6.87	7.67	–0.0421
**179**	13.92	6.87	7.05	–0.0421
**33**	15.76	6.89	8.87	–0.0422
**91a**	11.83	6.90	4.93	–0.0423
**44**	13.89	6.92	6.97	–0.0424
**72**	13.41	6.94	6.47	–0.0425
**138b**	12.40	6.95	5.45	–0.0426
**94e**	11.93	6.99	4.94	–0.0428
**48**	13.66	7.01	6.65	–0.0429
**139a**	11.60	7.06	4.54	–0.0432
**56**	13.54	7.09	6.45	–0.0434
**53**	13.81	7.22	6.59	–0.0441
**93a**	11.88	7.25	4.63	–0.0443
**146**	15.60	7.28	8.32	–0.0445
**185**	12.91	7.32	5.59	–0.0447
**177**	13.47	7.41	6.06	–0.0452
**60**	14.12	7.44	6.68	–0.0454
**135**	14.44	7.44	7.00	–0.0454
**1**	14.51	7.48	7.03	–0.0456
**95b**	15.42	7.49	7.93	–0.0457
**73**	13.26	7.51	5.75	–0.0458
**96a**	11.71	7.53	4.18	–0.0459
**4**	14.03	7.55	6.48	–0.0460
**41**	14.35	7.56	6.79	–0.0461
**110b**	13.18	7.58	5.60	–0.0462
**64**	14.36	7.58	6.78	–0.0462
**29a**	13.95	7.60	6.35	–0.0463
**123**	14.60	7.70	6.90	–0.0469
**117b**	14.28	7.74	6.54	–0.0471
**118b**	14.32	7.75	6.57	–0.0472
**111a**	14.46	7.75	6.71	–0.0472
**129**	14.23	7.75	6.48	–0.0472
**100**	11.62	7.82	3.80	–0.0476
**3**	14.57	7.84	6.73	–0.0477
**62**	14.30	7.84	6.46	–0.0477
**117a**	14.50	7.88	6.62	–0.0479
**94d**	14.75	7.91	6.84	–0.0481
**186**	11.69	7.91	3.78	–0.0481
**66**	14.31	7.93	6.38	–0.0482
**86b**	14.07	7.95	6.12	–0.0483
**21**	14.52	7.98	6.54	–0.0485
**88c**	13.76	8.02	5.74	–0.0487
**127**	15.52	8.03	7.49	–0.0488
**85a**	14.80	8.03	6.77	–0.0488
**94c**	15.12	8.03	7.09	–0.0488
**84c**	15.15	8.07	7.08	–0.0490
**142**	12.59	8.12	4.47	–0.0493

**180**	13.84	8.12	5.72	–0.0493
**75**	13.75	8.14	5.61	–0.0494
**111b**	16.25	8.17	8.08	–0.0496
**148**	13.72	8.19	5.53	–0.0497
**29b**	15.35	8.19	7.16	–0.0497
**136**	14.97	8.26	6.71	–0.0501
**2**	15.10	8.26	6.84	–0.0501
**61**	14.66	8.26	6.40	–0.0501
**31**	14.08	8.26	5.82	–0.0501
**70**	14.32	8.28	6.04	–0.0502
**90b**	14.96	8.28	6.68	–0.0502
**137**	14.08	8.33	5.75	–0.0505
**147**	16.28	8.33	7.95	–0.0505
**‹–E_{HB}(MTA, FBA)›, ‹π-contribution›**	13.62	7.23	6.39	

Table S52. Energy of the hydrogen bonding E_{HB} (in kcal mol^{-1}) of MTA and FBA methods, π-contribution to the total energy of arom-AHB interaction for compounds of the arom-AHB cluster within the $r_{O\cdots H}$ range of 1.75 – 1.80 Å

No compounds	**–E_{HB}(MTA)**	**–E_{HB}(FBA)**	**π-contribution**	**$r_{O\cdots H}$**
**48**	7.72	5.69	2.03	1.7937
**23**	7.67	5.75	1.92	1.7898
**22**	7.74	5.81	1.93	1.7865
**20**	7.56	5.85	1.71	1.7840
**11**	7.83	5.85	1.98	1.7839
**21**	7.92	5.90	2.02	1.7808
**61**	6.36	5.91	0.45	1.7805
**19**	7.88	5.95	1.93	1.7781
**24**	7.84	5.97	1.87	1.7769
**25**	7.75	5.98	1.77	1.7763
**39**	7.82	5.98	1.84	1.7761
**26**	7.75	5.99	1.76	1.7757
**16**	5.53	5.99	-0.46	1.7755
**30**	8.11	6.01	2.10	1.7746
**15**	5.72	6.02	-0.30	1.7740
**1**	7.85	6.05	1.80	1.7724
**17**	6.64	6.06	0.58	1.7720
**38**	7.87	6.07	1.80	1.7714
**29**	7.97	6.07	1.90	1.7713
**28**	7.87	6.07	1.80	1.7709
**32**	8.09	6.11	1.98	1.7690
**111**	8.25	6.11	2.14	1.7690
**27**	7.74	6.12	1.62	1.7683
**71**	6.96	6.15	0.81	1.7669
**46**	8.07	6.15	1.92	1.7668
**37**	7.99	6.16	1.83	1.7662
**112**	8.36	6.16	2.20	1.7662
**72**	6.87	6.18	0.69	1.7650

**35**	8.28	6.18	2.10	1.7648
**36**	8.11	6.18	1.93	1.7647
**73**	6.87	6.19	0.68	1.7645
**7**	6.99	6.20	0.79	1.7637
**4**	6.82	6.25	0.57	1.7610
**9**	8.06	6.25	1.81	1.7610
**33**	8.71	6.27	2.44	1.7601
**31**	8.65	6.27	2.38	1.7597
**34**	8.83	6.31	2.52	1.7579
**101c**	5.60	6.33	-0.73	1.7569
**102**	5.47	6.33	-0.86	1.7568
**45**	8.05	6.40	1.65	1.7527
**‹–E_{HB}(MTA, FBA)›, ‹π-contribution›**	7.54	6.08	1.47	

Table S53. Energy of the hydrogen bonding E_{HB} (in kcal mol^{-1}) of MTA and FBA methods, π-contribution to the total energy of arom-AHB interaction for compounds of the arom-AHB cluster within the $r_{O\cdots H}$ range of 1.70 – 1.75 Å

No compounds	–E_{HB}(MTA)	–E_{HB}(FBA)	π-contribution	$r_{O\cdots H}$
**6**	7.23	6.50	0.73	1.7474
**12**	6.98	6.69	0.29	1.7375
**110**	8.24	6.76	1.48	1.7339
**113**	8.69	6.79	1.90	1.7323
**40**	8.85	6.81	2.04	1.7310
**107**	7.44	6.84	0.60	1.7298
**13**	7.20	6.85	0.35	1.7289
**42**	8.60	6.92	1.68	1.7257
**57**	8.32	6.92	1.40	1.7253
**47**	10.45	6.93	3.52	1.7251
**74**	8.31	6.94	1.37	1.7246
**76**	8.48	6.95	1.53	1.7240
**75**	8.68	6.99	1.69	1.7220
**3**	8.20	7.01	1.19	1.7207
**82**	8.37	7.05	1.32	1.7188
**43**	8.93	7.10	1.83	1.7164
**10**	7.99	7.11	0.88	1.7157
**81**	8.86	7.12	1.74	1.7156
**116**	9.32	7.16	2.16	1.7136
**64**	8.98	7.16	1.82	1.7133
**56**	8.48	7.22	1.26	1.7106
**114**	8.73	7.23	1.50	1.7100
**117**	9.32	7.23	2.09	1.7098
**67**	9.56	7.25	2.31	1.7089
**78c**	8.84	7.25	1.59	1.7089
**78a**	8.49	7.28	1.21	1.7075
**41**	9.44	7.30	2.14	1.7067
**98**	7.59	7.32	0.27	1.7054
**78b**	8.47	7.35	1.12	1.7040

**86a**	10.29	7.36	2.93	1.7038
**118**	9.84	7.37	2.47	1.7032
**109**	7.69	7.41	0.28	1.7014
**83**	10.46	7.41	3.05	1.7012
**90b**	10.15	7.42	2.73	1.7009
**2**	8.57	7.43	1.14	1.7003
**85a**	10.28	7.43	2.85	1.7001
**108**	7.55	7.43	0.12	1.700
**‹−E_{HB}(MTA, FBA)›, ‹π-contribution›**	8.70	7.12	1.58	

Table S54. Energy of the hydrogen bonding E_{HB} (in kcal mol^{-1}) of MTA and FBA methods, π-contribution to the total energy of arom-AHB interaction for compounds of the arom-AHB cluster within the $r_{O\cdots H}$ range of 1.65 – 1.70 Å

**No compounds**	**−E_{HB}(MTA)**	**−E_{HB}(FBA)**	**π-contribution**	**$r_{O\cdots H}$**
**54**	8.81	7.46	1.35	1.6988
**89a**	10.56	7.47	3.09	1.6981
**8**	8.63	7.48	1.15	1.6980
**85b**	10.71	7.49	3.22	1.6974
**63**	8.77	7.51	1.26	1.6965
**100b**	9.46	7.51	1.95	1.6964
**80**	8.67	7.53	1.14	1.6954
**106**	7.34	7.53	-0.19	1.6954
**100a**	9.26	7.53	1.73	1.6952
**120**	9.95	7.54	2.41	1.6951
**94**	8.82	7.56	1.26	1.6942
**79**	9.27	7.60	1.67	1.6923
**91a**	10.61	7.60	3.01	1.6923
**87**	10.62	7.60	3.02	1.6920
**96**	9.07	7.64	1.43	1.6904
**65**	9.36	7.65	1.71	1.6899
**115**	8.75	7.70	1.05	1.6876
**95**	10.03	7.71	2.32	1.6869
**53**	9.41	7.72	1.69	1.6866
**66**	9.57	7.78	1.79	1.6837
**55**	9.17	7.81	1.36	1.6823
**99**	9.87	7.82	2.05	1.6819
**86b**	10.66	7.83	2.83	1.6814
**89b**	10.40	7.86	2.54	1.6799
**60**	7.96	7.86	0.10	1.6797
**68**	8.78	7.90	0.88	1.6783
**77**	9.89	7.92	1.97	1.6772
**97**	10.53	7.93	2.60	1.6767
**84a**	11.73	7.94	3.79	1.6763
**90a**	10.89	7.96	2.93	1.6755
**88**	10.64	7.96	2.68	1.6753
**92a**	10.45	7.97	2.48	1.6747
**84b**	11.78	8.02	3.76	1.6727

**91b**	10.98	8.07	2.91	1.6702
**92b**	10.77	8.08	2.69	1.6699
**122**	11.20	8.18	3.02	1.6654
**93**	10.72	8.21	2.51	1.6639
**101a**	11.25	8.27	2.98	1.6614
**100c**	11.48	8.27	3.21	1.6612
**58**	9.17	8.30	0.87	1.6600
**121**	11.09	8.39	2.70	1.6562
**44**	11.28	8.48	2.80	1.6523
**‹–E_{HB}(MTA, FBA)›, ‹π-contribution›**	9.96	7.82	2.14	

Table S55. Energy of the hydrogen bonding E_{HB} (in kcal mol^{-1}) of MTA and FBA methods, π-contribution to the total energy of arom-AHB for compounds of the arom-AHB cluster within the $r_{O\cdots H}$ range of 1.60 – 1.65 Å

No compounds	–E_{HB}(MTA)	–E_{HB}(FBA)	π-contribution	$r_{O\cdots H}$
**123**	9.86	8.55	1.31	1.6490
**101b**	10.87	8.57	2.30	1.6484
**49**	8.39	8.65	-0.26	1.6447
**59**	9.90	8.95	0.95	1.6321
**50**	9.25	9.19	0.06	1.6218
**52**	9.56	9.21	0.35	1.6210
**51**	9.41	9.51	-0.10	1.6089
**69**	10.19	9.56	0.63	1.6071
**‹–E_{HB}(MTA, FBA)›, ‹π-contribution›**	9.68	9.02	0.66	

Table S56. Energy of the hydrogen bonding E_{HB} (in kcal mol^{-1}) of MTA and FBA methods, π-contribution to the total energy of arom-AHB for compounds of the arom-AHB cluster within the $r_{O\cdots H}$ range of 1.60 – 1.80 Å (total range).

No compounds	–E_{HB}(MTA)	–E_{HB}(FBA)	π-contribution	$r_{O\cdots H}$
**48**	7.72	5.69	2.03	1.7937
**23**	7.67	5.75	1.92	1.7898
**22**	7.74	5.81	1.93	1.7865
**20**	7.56	5.85	1.71	1.7840
**11**	7.83	5.85	1.98	1.7839
**21**	7.92	5.90	2.02	1.7808
**61**	6.36	5.91	0.45	1.7805
**19**	7.88	5.95	1.93	1.7781
**24**	7.84	5.97	1.87	1.7769
**25**	7.75	5.98	1.77	1.7763
**39**	7.82	5.98	1.84	1.7761
**26**	7.75	5.99	1.76	1.7757
**16**	5.53	5.99	-0.46	1.7755
**30**	8.11	6.01	2.10	1.7746
**15**	5.72	6.02	-0.30	1.7740
**1**	7.85	6.05	1.80	1.7724
**17**	6.64	6.06	0.58	1.772

**38**	7.87	6.07	1.80	1.7714
**29**	7.97	6.07	1.90	1.7713
**28**	7.87	6.07	1.80	1.7709
**32**	8.09	6.11	1.98	1.7690
**111**	8.25	6.11	2.14	1.769
**27**	7.74	6.12	1.62	1.7683
**71**	6.96	6.15	0.81	1.7669
**46**	8.07	6.15	1.92	1.7668
**37**	7.99	6.16	1.83	1.7662
**112**	8.36	6.16	2.20	1.7662
**72**	6.87	6.18	0.69	1.7650
**35**	8.28	6.18	2.10	1.7648
**36**	8.11	6.18	1.93	1.7647
**73**	6.87	6.19	0.68	1.7645
**7**	6.99	6.20	0.79	1.7637
**4**	6.82	6.25	0.57	1.7610
**9**	8.06	6.25	1.81	1.7610
**33**	8.71	6.27	2.44	1.7601
**31**	8.65	6.27	2.38	1.7597
**34**	8.83	6.31	2.52	1.7579
**101c**	5.60	6.33	-0.73	1.7569
**102**	5.47	6.33	-0.86	1.7568
**45**	8.05	6.40	1.65	1.7527
**5**	7.09	6.42	0.67	1.7516
**6**	7.23	6.50	0.73	1.7474
**12**	6.98	6.69	0.29	1.7375
**110**	8.24	6.76	1.48	1.7339
**113**	8.69	6.79	1.90	1.7323
**40**	8.85	6.81	2.04	1.7310
**107**	7.44	6.84	0.60	1.7298
**13**	7.20	6.85	0.35	1.7289
**42**	8.60	6.92	1.68	1.7257
**57**	8.32	6.92	1.40	1.7253
**47**	10.45	6.93	3.52	1.7251
**74**	8.31	6.94	1.37	1.7246
**76**	8.48	6.95	1.53	1.7240
**75**	8.68	6.99	1.69	1.7220
**3**	8.20	7.01	1.19	1.7207
**82**	8.37	7.05	1.32	1.7188
**43**	8.93	7.10	1.83	1.7164
**10**	7.99	7.11	0.88	1.7157
**81**	8.86	7.12	1.74	1.7156
**116**	9.32	7.16	2.16	1.7136
**64**	8.98	7.16	1.82	1.7133
**56**	8.48	7.22	1.26	1.7106
**114**	8.73	7.23	1.50	1.7100
**117**	9.32	7.23	2.09	1.7098
**67**	9.56	7.25	2.31	1.7089

**78c**	8.84	7.25	1.59	1.7089
**78a**	8.49	7.28	1.21	1.7075
**41**	9.44	7.30	2.14	1.7067
**98**	7.59	7.32	0.27	1.7054
**78b**	8.47	7.35	1.12	1.7040
**86a**	10.29	7.36	2.93	1.7038
**118**	9.84	7.37	2.47	1.7032
**109**	7.69	7.41	0.28	1.7014
**83**	10.46	7.41	3.05	1.7012
**90b**	10.15	7.42	2.73	1.7009
**2**	8.57	7.43	1.14	1.7003
**85a**	10.28	7.43	2.85	1.7001
**108**	7.55	7.43	0.12	1.7000
**54**	8.81	7.46	1.35	1.6988
**89a**	10.56	7.47	3.09	1.6981
**8**	8.63	7.48	1.15	1.6980
**85b**	10.71	7.49	3.22	1.6974
**63**	8.77	7.51	1.26	1.6965
**100b**	9.46	7.51	1.95	1.6964
**80**	8.67	7.53	1.14	1.6954
**106**	7.34	7.53	-0.19	1.6954
**100a**	9.26	7.53	1.73	1.6952
**120**	9.95	7.54	2.41	1.6951
**94**	8.82	7.56	1.26	1.6942
**79**	9.27	7.60	1.67	1.6923
**91a**	10.61	7.60	3.01	1.6923
**87**	10.62	7.60	3.02	1.6920
**96**	9.07	7.64	1.43	1.6904
**65**	9.36	7.65	1.71	1.6899
**115**	8.75	7.70	1.05	1.6876
**95**	10.03	7.71	2.32	1.6869
**53**	9.41	7.72	1.69	1.6866
**66**	9.57	7.78	1.79	1.6837
**55**	9.17	7.81	1.36	1.6823
**99**	9.87	7.82	2.05	1.6819
**86b**	10.66	7.83	2.83	1.6814
**89b**	10.4	7.86	2.54	1.6799
**60**	7.96	7.86	0.10	1.6797
**68**	8.78	7.90	0.88	1.6783
**77**	9.89	7.92	1.97	1.6772
**97**	10.53	7.93	2.60	1.6767
**84a**	11.73	7.94	3.79	1.6763
**90a**	10.89	7.96	2.93	1.6755
**88**	10.64	7.96	2.68	1.6753
**92a**	10.45	7.97	2.48	1.6747
**84b**	11.78	8.02	3.76	1.6727
**91b**	10.98	8.07	2.91	1.6702
**92b**	10.77	8.08	2.69	1.6699

**122**	11.2	8.18	3.02	1.6654
**93**	10.72	8.21	2.51	1.6639
**101a**	11.25	8.27	2.98	1.6614
**100c**	11.48	8.27	3.21	1.6612
**58**	9.17	8.30	0.87	1.6600
**121**	11.09	8.39	2.70	1.6562
**44**	11.28	8.48	2.80	1.6523
**123**	9.86	8.55	1.31	1.6490
**101b**	10.87	8.57	2.30	1.6484
**49**	8.39	8.65	-0.26	1.6447
**59**	9.90	8.95	0.95	1.6321
**50**	9.25	9.19	0.06	1.6218
**52**	9.56	9.21	0.35	1.6210
**51**	9.41	9.51	-0.10	1.6089
**69**	10.19	9.56	0.63	1.6071
**‹$-E_{HB}$(MTA, FBA)›, ‹π-contribution›**	8.80	7.13	1.67	

Table S57. Energy of the hydrogen bonding E_{HB} (in kcal mol^{-1}) of MTA and FBA methods, π-contribution to the total energy of arom-AHB interaction for compounds of the arom-AHB cluster within the ρ_{BCP} range of 0.030 – 0.036 a.u.

**No compounds**	**$-E_{HB}$(MTA)**	**$-E_{HB}$(FBA)**	**π-contribution**	**ρ_{BCP}**
**18**	5.38	4.38	1.00	0.0322
**14**	5.69	4.59	1.10	0.0333
**46**	8.07	4.79	3.28	0.0343
**3**	8.20	4.90	3.30	0.0349
**48**	7.72	5.01	2.71	0.0355
**23**	7.67	5.07	2.60	0.0358
**22**	7.74	5.11	2.63	0.0360
**‹$-E_{HB}$(MTA, FBA)›, ‹π-contribution›**	7.21	4.84	2.37	

Table S58. Energy of the hydrogen bonding E_{HB} (in kcal mol^{-1}) of MTA and FBA methods, π-contribution to the total energy of arom-AHB interaction for compounds of the arom-AHB cluster within the ρ_{BCP} range of 0.036 – 0.041 a.u.

**No compounds**	**$-E_{HB}$(MTA)**	**$-E_{HB}$(FBA)**	**π-contribution**	**ρ_{BCP}**
**61**	6.36	5.13	1.23	0.0361
**20**	7.56	5.15	2.41	0.0362
**11**	7.83	5.17	2.66	0.0363
**21**	7.92	5.21	2.71	0.0365
**16**	5.53	5.24	0.29	0.0367
**19**	7.88	5.26	2.62	0.0368
**24**	7.84	5.26	2.58	0.0368
**15**	5.72	5.28	0.44	0.0369
**25**	7.75	5.28	2.47	0.0369
**39**	7.82	5.28	2.54	0.0369
**26**	7.75	5.30	2.45	0.0370
**17**	6.64	5.32	1.32	0.0371

**30**	8.11	5.32	2.79	0.0371
**1**	7.85	5.34	2.51	0.0372
**29**	7.97	5.36	2.61	0.0373
**38**	7.87	5.36	2.51	0.0373
**28**	7.87	5.38	2.49	0.0374
**111**	8.25	5.38	2.87	0.0374
**71**	6.96	5.40	1.56	0.0375
**27**	7.74	5.42	2.32	0.0376
**32**	8.09	5.42	2.67	0.0376
**112**	8.36	5.42	2.94	0.0376
**72**	6.87	5.44	1.43	0.0377
**73**	6.87	5.44	1.43	0.0377
**37**	7.99	5.45	2.54	0.0378
**7**	6.99	5.47	1.52	0.0379
**35**	8.28	5.47	2.81	0.0379
**36**	8.11	5.47	2.64	0.0379
**4**	6.82	5.49	1.33	0.0380
**9**	8.06	5.53	2.53	0.0382
**102**	5.47	5.53	-0.06	0.0382
**101c**	5.60	5.55	0.05	0.0383
**31**	8.65	5.57	3.08	0.0384
**33**	8.71	5.57	3.14	0.0384
**34**	8.83	5.63	3.20	0.0387
**45**	8.05	5.67	2.38	0.0389
**5**	7.09	5.68	1.41	0.0390
**6**	7.23	5.76	1.47	0.0394
**12**	6.98	5.91	1.07	0.0402
**110**	8.24	6.03	2.21	0.0408
**107**	7.44	6.05	1.39	0.0409
**113**	8.69	6.07	2.62	0.0410
**‹−E_{HB}(MTA, FBA)›, ‹π-contribution›**	7.54	5.46	2.08	

Table S59. Energy of the hydrogen bonding E_{HB} (in kcal mol^{-1}) of MTA and FBA methods, π-contribution to the total energy of arom-AHB interaction for compounds of the arom-AHB cluster within the ρ_{BCP} range of 0.041 – 0.046 a.u.

**No compounds**	**−E_{HB}(MTA)**	**−E_{HB}(FBA)**	**π-contribution**	**ρ_{BCP}**
**13**	7.20	6.09	1.11	0.0411
**40**	8.85	6.09	2.76	0.0411
**57**	8.32	6.18	2.14	0.0416
**74**	8.31	6.18	2.13	0.0416
**42**	8.60	6.20	2.40	0.0417
**76**	8.48	6.20	2.28	0.0417
**47**	10.45	6.26	4.19	0.0420
**75**	8.68	6.26	2.42	0.0420
**82**	8.37	6.32	2.05	0.0423
**10**	7.99	6.34	1.65	0.0424
**43**	8.93	6.37	2.56	0.0426

**81**	8.86	6.37	2.49	0.0426
**116**	9.32	6.41	2.91	0.0428
**64**	8.98	6.43	2.55	0.0429
**108**	7.55	6.45	1.10	0.0430
**56**	8.48	6.47	2.01	0.0431
**78c**	8.84	6.47	2.37	0.0431
**109**	7.69	6.47	1.22	0.0431
**98**	7.59	6.49	1.10	0.0432
**67**	9.56	6.51	3.05	0.0433
**78a**	8.49	6.51	1.98	0.0433
**117**	9.32	6.53	2.79	0.0434
**114**	8.73	6.57	2.16	0.0436
**41**	9.44	6.58	2.86	0.0437
**78b**	8.47	6.58	1.89	0.0437
**86a**	10.29	6.58	3.71	0.0437
**90b**	10.15	6.62	3.53	0.0439
**83**	10.46	6.64	3.82	0.0440
**85a**	10.28	6.64	3.64	0.0440
**100b**	9.46	6.66	2.80	0.0441
**118**	9.84	6.66	3.18	0.0441
**100a**	9.26	6.68	2.58	0.0442
**2**	8.57	6.70	1.87	0.0443
**8**	8.63	6.70	1.93	0.0443
**89a**	10.56	6.70	3.86	0.0443
**85b**	10.71	6.72	3.99	0.0444
**54**	8.81	6.74	2.07	0.0445
**63**	8.77	6.78	1.99	0.0447
**80**	8.67	6.78	1.89	0.0447
**94**	8.82	6.79	2.03	0.0448
**87**	10.62	6.81	3.81	0.0449
**91a**	10.61	6.81	3.80	0.0449
**106**	7.34	6.81	0.53	0.0449
**120**	9.95	6.81	3.14	0.0449
**79**	9.27	6.83	2.44	0.0450
**115**	8.75	6.85	1.90	0.0451
**96**	9.07	6.87	2.20	0.0452
**65**	9.36	6.91	2.45	0.0454
**95**	10.03	6.95	3.08	0.0456
**99**	9.87	6.97	2.90	0.0457
**53**	9.41	6.99	2.42	0.0458
**60**	7.96	6.99	0.97	0.0458
**‹$-E_{HB}$(MTA, FBA)›, ‹π-contribution›**	9.06	6.58	2.48	

Table S60. Energy of the hydrogen bonding E_{HB} (in kcal mol^{-1}) of MTA and FBA methods, π-contribution to the total energy of arom-AHB interaction for compounds of the arom-AHB cluster within the ρ_{BCP} range of 0.046 – 0.051 a.u.

No comp	$-E_{HB}$(MTA)	$-E_{HB}$(FBA)	π-contribution	ρ_{BCP}

**66**	9.57	7.04	2.53	0.0461
**86b**	10.66	7.04	3.62	0.0461
**55**	9.17	7.06	2.11	0.0462
**89b**	10.4	7.06	3.34	0.0462
**68**	8.78	7.16	1.62	0.0467
**90a**	10.89	7.16	3.73	0.0467
**92b**	10.77	7.16	3.61	0.0467
**97**	10.53	7.16	3.37	0.0467
**77**	9.89	7.18	2.71	0.0468
**84a**	11.73	7.18	4.55	0.0468
**88**	10.64	7.18	3.46	0.0468
**84b**	11.78	7.23	4.55	0.0471
**91b**	10.98	7.27	3.71	0.0473
**92a**	10.45	7.29	3.16	0.0474
**93**	10.72	7.41	3.31	0.0480
**122**	11.20	7.43	3.77	0.0481
**101a**	11.25	7.48	3.77	0.0484
**100c**	11.48	7.50	3.98	0.0485
**58**	9.17	7.54	1.63	0.0487
**121**	11.09	7.68	3.41	0.0494
**44**	11.28	7.75	3.53	0.0498
**101b**	10.87	7.81	3.06	0.0501
**123**	9.86	7.81	2.05	0.0501
**49**	8.39	7.89	0.50	0.0505
**‹−E_{HB}(MTA, FBA)›, ‹π-contribution›**	10.48	7.35	3.13	

Table S61. Energy of the hydrogen bonding E_{HB} (in kcal mol^{-1}) of MTA and FBA methods, π-contribution to the total energy of arom-AHB interaction for arom-AHB structures within the ρ_{BCP} range of 0.030 – 0.051 a.u. (total range).

**No compounds**	**−E_{HB}(MTA)**	**−E_{HB}(FBA)**	**π-contribution**	**ρ_{BCP}**
**18**	5.38	4.38	1.00	0.0322
**14**	5.69	4.59	1.10	0.0333
**46**	8.07	4.79	3.28	0.0343
**3**	8.20	4.90	3.30	0.0349
**48**	7.72	5.01	2.71	0.0355
**23**	7.67	5.07	2.60	0.0358
**22**	7.74	5.11	2.63	0.0360
**61**	6.36	5.13	1.23	0.0361
**20**	7.56	5.15	2.41	0.0362
**11**	7.83	5.17	2.66	0.0363
**21**	7.92	5.21	2.71	0.0365
**16**	5.53	5.24	0.29	0.0367
**19**	7.88	5.26	2.62	0.0368
**24**	7.84	5.26	2.58	0.0368
**15**	5.72	5.28	0.44	0.0369
**25**	7.75	5.28	2.47	0.0369
**39**	7.82	5.28	2.54	0.0369

**26**	7.75	5.30	2.45	0.0370
**17**	6.64	5.32	1.32	0.0371
**30**	8.11	5.32	2.79	0.0371
**1**	7.85	5.34	2.51	0.0372
**29**	7.97	5.36	2.61	0.0373
**38**	7.87	5.36	2.51	0.0373
**28**	7.87	5.38	2.49	0.0374
**111**	8.25	5.38	2.87	0.0374
**71**	6.96	5.40	1.56	0.0375
**27**	7.74	5.42	2.32	0.0376
**32**	8.09	5.42	2.67	0.0376
**112**	8.36	5.42	2.94	0.0376
**72**	6.87	5.44	1.43	0.0377
**73**	6.87	5.44	1.43	0.0377
**37**	7.99	5.45	2.54	0.0378
**7**	6.99	5.47	1.52	0.0379
**35**	8.28	5.47	2.81	0.0379
**36**	8.11	5.47	2.64	0.0379
**4**	6.82	5.49	1.33	0.0380
**9**	8.06	5.53	2.53	0.0382
**102**	5.47	5.53	-0.06	0.0382
**101c**	5.60	5.55	0.05	0.0383
**31**	8.65	5.57	3.08	0.0384
**33**	8.71	5.57	3.14	0.0384
**34**	8.83	5.63	3.20	0.0387
**45**	8.05	5.67	2.38	0.0389
**5**	7.09	5.68	1.41	0.0390
**6**	7.23	5.76	1.47	0.0394
**12**	6.98	5.91	1.07	0.0402
**110**	8.24	6.03	2.21	0.0408
**107**	7.44	6.05	1.39	0.0409
**113**	8.69	6.07	2.62	0.0410
**13**	7.20	6.09	1.11	0.0411
**40**	8.85	6.09	2.76	0.0411
**57**	8.32	6.18	2.14	0.0416
**74**	8.31	6.18	2.13	0.0416
**42**	8.60	6.20	2.40	0.0417
**76**	8.48	6.20	2.28	0.0417
**47**	10.45	6.26	4.19	0.0420
**75**	8.68	6.26	2.42	0.0420
**82**	8.37	6.32	2.05	0.0423
**10**	7.99	6.34	1.65	0.0424
**43**	8.93	6.37	2.56	0.0426
**81**	8.86	6.37	2.49	0.0426
**116**	9.32	6.41	2.91	0.0428
**64**	8.98	6.43	2.55	0.0429
**108**	7.55	6.45	1.10	0.0430
**56**	8.48	6.47	2.01	0.0431

**78c**	8.84	6.47	2.37	0.0431
**109**	7.69	6.47	1.22	0.0431
**98**	7.59	6.49	1.10	0.0432
**67**	9.56	6.51	3.05	0.0433
**78a**	8.49	6.51	1.98	0.0433
**117**	9.32	6.53	2.79	0.0434
**114**	8.73	6.57	2.16	0.0436
**41**	9.44	6.58	2.86	0.0437
**78b**	8.47	6.58	1.89	0.0437
**86a**	10.29	6.58	3.71	0.0437
**90b**	10.15	6.62	3.53	0.0439
**83**	10.46	6.64	3.82	0.0440
**85a**	10.28	6.64	3.64	0.0440
**100b**	9.46	6.66	2.80	0.0441
**118**	9.84	6.66	3.18	0.0441
**100a**	9.26	6.68	2.58	0.0442
**2**	8.57	6.70	1.87	0.0443
**8**	8.63	6.70	1.93	0.0443
**89a**	10.56	6.70	3.86	0.0443
**85b**	10.71	6.72	3.99	0.0444
**54**	8.81	6.74	2.07	0.0445
**63**	8.77	6.78	1.99	0.0447
**80**	8.67	6.78	1.89	0.0447
**94**	8.82	6.79	2.03	0.0448
**87**	10.62	6.81	3.81	0.0449
**91a**	10.61	6.81	3.80	0.0449
**106**	7.34	6.81	0.53	0.0449
**120**	9.95	6.81	3.14	0.0449
**79**	9.27	6.83	2.44	0.0450
**115**	8.75	6.85	1.90	0.0451
**96**	9.07	6.87	2.20	0.0452
**65**	9.36	6.91	2.45	0.0454
**95**	10.03	6.95	3.08	0.0456
**99**	9.87	6.97	2.90	0.0457
**53**	9.41	6.99	2.42	0.0458
**60**	7.96	6.99	0.97	0.0458
**66**	9.57	7.04	2.53	0.0461
**86b**	10.66	7.04	3.62	0.0461
**55**	9.17	7.06	2.11	0.0462
**89b**	10.4	7.06	3.34	0.0462
**68**	8.78	7.16	1.62	0.0467
**90a**	10.89	7.16	3.73	0.0467
**92b**	10.77	7.16	3.61	0.0467
**97**	10.53	7.16	3.37	0.0467
**77**	9.89	7.18	2.71	0.0468
**84a**	11.73	7.18	4.55	0.0468
**88**	10.64	7.18	3.46	0.0468
**84b**	11.78	7.23	4.55	0.0471

**91b**	10.98	7.27	3.71	0.0473
**92a**	10.45	7.29	3.16	0.0474
**93**	10.72	7.41	3.31	0.0480
**122**	11.20	7.43	3.77	0.0481
**101a**	11.25	7.48	3.77	0.0484
**100c**	11.48	7.50	3.98	0.0485
**58**	9.17	7.54	1.63	0.0487
**121**	11.09	7.68	3.41	0.0494
**44**	11.28	7.75	3.53	0.0498
**101b**	10.87	7.81	3.06	0.0501
**123**	9.86	7.81	2.05	0.0501
**49**	8.39	7.89	0.50	0.0505
**‹–E_{HB}(MTA, FBA)›, ‹π-contribution›**	8.72	6.26	2.46	

Table S62. Energy of the hydrogen bonding E_{HB} (in kcal mol^{-1}) of MTA and FBA methods, π-contribution to the total energy of arom-AHB interaction for compounds of the arom-AHB cluster within the V_{BCP} range of –0.030 ÷ –0.036 a.u.

No compounds	–E_{HB}(MTA)	–E_{HB}(FBA)	π-contribution	V_{BCP}
**14**	5.69	4.92	0.77	–0.0309
**48**	7.72	5.34	2.38	–0.0333
**23**	7.67	5.39	2.28	–0.0336
**22**	7.74	5.44	2.30	–0.0339
**11**	7.83	5.48	2.35	–0.0341
**20**	7.56	5.48	2.08	–0.0341
**61**	6.36	5.51	0.85	–0.0343
**21**	7.92	5.53	2.39	–0.0344
**19**	7.88	5.58	2.30	–0.0347
**24**	7.84	5.60	2.24	–0.0348
**25**	7.75	5.60	2.15	–0.0348
**26**	7.75	5.62	2.13	–0.0349
**39**	7.82	5.62	2.20	–0.0349
**30**	8.11	5.63	2.48	–0.0350
**37**	7.99	5.63	2.36	–0.0350
**16**	5.53	5.65	-0.12	–0.0351
**1**	7.85	5.67	2.18	–0.0352
**15**	5.72	5.67	0.05	–0.0352
**111**	8.25	5.67	2.58	–0.0352
**29**	7.97	5.69	2.28	–0.0353
**38**	7.87	5.69	2.18	–0.0353
**28**	7.87	5.70	2.17	–0.0354
**112**	8.36	5.70	2.66	–0.0354
**17**	6.64	5.72	0.92	–0.0355
**32**	8.09	5.72	2.37	–0.0355
**27**	7.74	5.74	2.00	–0.0356
**46**	8.07	5.74	2.33	–0.0356
**71**	6.96	5.76	1.20	–0.0357
**35**	8.28	5.79	2.49	–0.0359

**72**	6.87	5.79	1.08	–0.0359
**73**	6.87	5.79	1.08	–0.0359
**‹–E_{HB}(MTA, FBA)›, ‹π-contribution›**	7.50	5.61	1.89	

Table S63. Energy of the hydrogen bonding E_{HB} (in kcal mol^{-1}) of MTA and FBA methods, π-contribution to the total energy of arom-AHB interaction for compounds of the arom-AHB cluster within the V_{BCP} range of –0.036 ÷ –0.041 a.u.

**No compounds**	**–E_{HB}(MTA)**	**–E_{HB}(FBA)**	**π-contribution**	**V_{BCP}**
**36**	8.11	5.81	2.30	–0.0360
**7**	6.99	5.84	1.15	–0.0362
**4**	6.82	5.86	0.96	–0.0363
**9**	8.06	5.86	2.20	–0.0363
**31**	8.65	5.88	2.77	–0.0364
**33**	8.71	5.88	2.83	–0.0364
**34**	8.83	5.93	2.90	–0.0367
**101c**	5.60	5.96	-0.36	–0.0369
**45**	8.05	5.98	2.07	–0.0370
**102**	5.47	5.98	-0.51	–0.0370
**5**	7.09	6.05	1.04	–0.0374
**6**	7.23	6.14	1.09	–0.0379
**110**	8.24	6.40	1.84	–0.0394
**40**	8.85	6.43	2.42	–0.0396
**107**	7.44	6.43	1.01	–0.0396
**113**	8.69	6.45	2.24	–0.0397
**12**	6.98	6.50	0.48	–0.0400
**13**	7.20	6.50	0.70	–0.0400
**47**	10.45	6.54	3.91	–0.0402
**42**	8.60	6.57	2.03	–0.0404
**57**	8.32	6.59	1.73	–0.0405
**74**	8.31	6.61	1.70	–0.0406
**76**	8.48	6.61	1.87	–0.0406
**75**	8.68	6.66	2.02	–0.0409
**‹–E_{HB}(MTA, FBA)›, ‹π-contribution›**	7.91	6.23	1.68	

Table S64. Energy of the hydrogen bonding E_{HB} (in kcal mol^{-1}) of MTA and FBA methods, π-contribution to the total energy of arom-AHB interaction for compounds of the arom-AHB cluster within the V_{BCP} range of –0.041 ÷ –0.046 a.u.

**No compounds**	**–E_{HB}(MTA)**	**–E_{HB}(FBA)**	**π-contribution**	**V_{BCP}**
**3**	8.20	6.68	1.52	–0.0410
**10**	7.99	6.75	1.24	–0.0414
**43**	8.93	6.75	2.18	–0.0414
**82**	8.37	6.75	1.62	–0.0414
**81**	8.86	6.78	2.08	–0.0416
**64**	8.98	6.80	2.18	–0.0417
**116**	9.32	6.85	2.47	–0.0420
**117**	9.32	6.87	2.45	–0.0421

**56**	8.48	6.89	1.59	–0.0422
**114**	8.73	6.89	1.84	–0.0422
**108**	7.55	6.92	0.63	–0.0424
**109**	7.69	6.92	0.77	–0.0424
**41**	9.44	6.94	2.50	–0.0425
**67**	9.56	6.94	2.62	–0.0425
**78c**	8.84	6.94	1.90	–0.0425
**78a**	8.49	6.97	1.52	–0.0427
**98**	7.59	6.97	0.62	–0.0427
**118**	9.84	6.99	2.85	–0.0428
**86a**	10.29	7.02	3.27	–0.0430
**78b**	8.47	7.04	1.43	–0.0431
**83**	10.46	7.09	3.37	–0.0434
**90b**	10.15	7.11	3.04	–0.0435
**2**	8.57	7.13	1.44	–0.0436
**8**	8.63	7.13	1.50	–0.0436
**85a**	10.28	7.13	3.15	–0.0436
**100a**	9.26	7.13	2.13	–0.0436
**54**	8.81	7.15	1.66	–0.0437
**89a**	10.56	7.15	3.41	–0.0437
**100b**	9.46	7.16	2.30	–0.0438
**85b**	10.71	7.18	3.53	–0.0439
**120**	9.95	7.18	2.77	–0.0439
**106**	7.34	7.20	0.14	–0.0440
**63**	8.77	7.22	1.55	–0.0441
**80**	8.67	7.23	1.44	–0.0442
**94**	8.82	7.27	1.55	–0.0444
**79**	9.27	7.30	1.97	–0.0446
**91a**	10.61	7.30	3.31	–0.0446
**115**	8.75	7.32	1.43	–0.0447
**65**	9.36	7.34	2.02	–0.0448
**87**	10.62	7.34	3.28	–0.0448
**96**	9.07	7.35	1.72	–0.0449
**95**	10.03	7.37	2.66	–0.0450
**53**	9.41	7.42	1.99	–0.0453
**66**	9.57	7.48	2.09	–0.0456
**99**	9.87	7.49	2.38	–0.0457
**60**	7.96	7.51	0.45	–0.0458
**‹–E_{HB}(MTA, FBA)›, ‹π-contribution›**	9.13	7.10	2.03	

Table S65. Energy of the hydrogen bonding E_{HB} (in kcal mol^{-1}) of MTA and FBA methods, π-contribution to the total energy of arom-AHB interaction for compounds of the arom-AHB cluster within the V_{BCP} range of –0.046 ÷ –0.051 a.u.

No compounds	–E_{HB}(MTA)	–E_{HB}(FBA)	π-contribution	V_{BCP}
**55**	9.17	7.55	1.62	–0.0460
**86b**	10.66	7.55	3.11	–0.0460
**89b**	10.40	7.58	2.82	–0.0462

**77**	9.89	7.63	2.26	–0.0465
**68**	8.78	7.65	1.13	–0.0466
**84a**	11.73	7.65	4.08	–0.0466
**90a**	10.89	7.67	3.22	–0.0467
**88**	10.64	7.68	2.96	–0.0468
**97**	10.53	7.68	2.85	–0.0468
**92a**	10.45	7.70	2.75	–0.0469
**84b**	11.78	7.75	4.03	–0.0472
**92b**	10.77	7.81	2.96	–0.0475
**91b**	10.98	7.82	3.16	–0.0476
**122**	11.20	7.93	3.27	–0.0482
**93**	10.72	7.96	2.76	–0.0484
**100c**	11.48	8.05	3.43	–0.0489
**101a**	11.25	8.07	3.18	–0.0490
**58**	9.17	8.10	1.07	–0.0492
**121**	11.09	8.12	2.97	–0.0493
**44**	11.28	8.17	3.11	–0.0496
**123**	9.86	8.36	1.50	–0.0507
**101b**	10.87	8.40	2.47	–0.0509
**‹–E_{HB}(MTA, FBA)›, ‹π-contribution›**	10.62	7.86	2.76	

Table S66. Energy of the hydrogen bonding E_{HB} (in kcal mol^{-1}) of MTA and FBA methods, π-contribution to the total energy of arom-AHB interaction for compounds of the arom-AHB cluster within the V_{BCP} range of –0.030 ÷ –0.051 a.u. (total range).

**No compounds**	**–E_{HB}(MTA)**	**–E_{HB}(FBA)**	**π-contribution**	**V_{BCP}**
**14**	5.69	4.92	0.77	–0.0309
**48**	7.72	5.34	2.38	–0.0333
**23**	7.67	5.39	2.28	–0.0336
**22**	7.74	5.44	2.30	–0.0339
**11**	7.83	5.48	2.35	–0.0341
**20**	7.56	5.48	2.08	–0.0341
**61**	6.36	5.51	0.85	–0.0343
**21**	7.92	5.53	2.39	–0.0344
**19**	7.88	5.58	2.30	–0.0347
**24**	7.84	5.60	2.24	–0.0348
**25**	7.75	5.60	2.15	–0.0348
**26**	7.75	5.62	2.13	–0.0349
**39**	7.82	5.62	2.20	–0.0349
**30**	8.11	5.63	2.48	–0.0350
**37**	7.99	5.63	2.36	–0.0350
**16**	5.53	5.65	-0.12	–0.0351
**1**	7.85	5.67	2.18	–0.0352
**15**	5.72	5.67	0.05	–0.0352
**111**	8.25	5.67	2.58	–0.0352
**29**	7.97	5.69	2.28	–0.0353
**38**	7.87	5.69	2.18	–0.0353
**28**	7.87	5.70	2.17	–0.0354

**112**	8.36	5.70	2.66	–0.0354
**17**	6.64	5.72	0.92	–0.0355
**32**	8.09	5.72	2.37	–0.0355
**27**	7.74	5.74	2.00	–0.0356
**46**	8.07	5.74	2.33	–0.0356
**71**	6.96	5.76	1.20	–0.0357
**35**	8.28	5.79	2.49	–0.0359
**72**	6.87	5.79	1.08	–0.0359
**73**	6.87	5.79	1.08	–0.0359
**36**	8.11	5.81	2.30	–0.0360
**7**	6.99	5.84	1.15	–0.0362
**4**	6.82	5.86	0.96	–0.0363
**9**	8.06	5.86	2.20	–0.0363
**31**	8.65	5.88	2.77	–0.0364
**33**	8.71	5.88	2.83	–0.0364
**34**	8.83	5.93	2.90	–0.0367
**101c**	5.60	5.96	-0.36	–0.0369
**45**	8.05	5.98	2.07	–0.0370
**102**	5.47	5.98	-0.51	–0.0370
**5**	7.09	6.05	1.04	–0.0374
**6**	7.23	6.14	1.09	–0.0379
**110**	8.24	6.40	1.84	–0.0394
**40**	8.85	6.43	2.42	–0.0396
**107**	7.44	6.43	1.01	–0.0396
**113**	8.69	6.45	2.24	–0.0397
**12**	6.98	6.50	0.48	–0.0400
**13**	7.20	6.50	0.70	–0.0400
**47**	10.45	6.54	3.91	–0.0402
**42**	8.60	6.57	2.03	–0.0404
**57**	8.32	6.59	1.73	–0.0405
**74**	8.31	6.61	1.70	–0.0406
**76**	8.48	6.61	1.87	–0.0406
**75**	8.68	6.66	2.02	–0.0409
**3**	8.20	6.68	1.52	–0.0410
**10**	7.99	6.75	1.24	–0.0414
**43**	8.93	6.75	2.18	–0.0414
**82**	8.37	6.75	1.62	–0.0414
**81**	8.86	6.78	2.08	–0.0416
**64**	8.98	6.80	2.18	–0.0417
**116**	9.32	6.85	2.47	–0.0420
**117**	9.32	6.87	2.45	–0.0421
**56**	8.48	6.89	1.59	–0.0422
**114**	8.73	6.89	1.84	–0.0422
**108**	7.55	6.92	0.63	–0.0424
**109**	7.69	6.92	0.77	–0.0424
**41**	9.44	6.94	2.50	–0.0425
**67**	9.56	6.94	2.62	–0.0425
**78c**	8.84	6.94	1.90	–0.0425

**78a**	8.49	6.97	1.52	−0.0427
**98**	7.59	6.97	0.62	−0.0427
**118**	9.84	6.99	2.85	−0.0428
**86a**	10.29	7.02	3.27	−0.0430
**78b**	8.47	7.04	1.43	−0.0431
**83**	10.46	7.09	3.37	−0.0434
**90b**	10.15	7.11	3.04	−0.0435
**2**	8.57	7.13	1.44	−0.0436
**8**	8.63	7.13	1.50	−0.0436
**85a**	10.28	7.13	3.15	−0.0436
**100a**	9.26	7.13	2.13	−0.0436
**54**	8.81	7.15	1.66	−0.0437
**89a**	10.56	7.15	3.41	−0.0437
**100b**	9.46	7.16	2.30	−0.0438
**85b**	10.71	7.18	3.53	−0.0439
**120**	9.95	7.18	2.77	−0.0439
**106**	7.34	7.20	0.14	−0.0440
**63**	8.77	7.22	1.55	−0.0441
**80**	8.67	7.23	1.44	−0.0442
**94**	8.82	7.27	1.55	−0.0444
**79**	9.27	7.30	1.97	−0.0446
**91a**	10.61	7.30	3.31	−0.0446
**115**	8.75	7.32	1.43	−0.0447
**65**	9.36	7.34	2.02	−0.0448
**87**	10.62	7.34	3.28	−0.0448
**96**	9.07	7.35	1.72	−0.0449
**95**	10.03	7.37	2.66	−0.0450
**53**	9.41	7.42	1.99	−0.0453
**66**	9.57	7.48	2.09	−0.0456
**99**	9.87	7.49	2.38	−0.0457
**60**	7.96	7.51	0.45	−0.0458
**55**	9.17	7.55	1.62	−0.0460
**86b**	10.66	7.55	3.11	−0.0460
**89b**	10.4	7.58	2.82	−0.0462
**77**	9.89	7.63	2.26	−0.0465
**68**	8.78	7.65	1.13	−0.0466
**84a**	11.73	7.65	4.08	−0.0466
**90a**	10.89	7.67	3.22	−0.0467
**88**	10.64	7.68	2.96	−0.0468
**97**	10.53	7.68	2.85	−0.0468
**92a**	10.45	7.70	2.75	−0.0469
**84b**	11.78	7.75	4.03	−0.0472
**92b**	10.77	7.81	2.96	−0.0475
**91b**	10.98	7.82	3.16	−0.0476
**122**	11.20	7.93	3.27	−0.0482
**93**	10.72	7.96	2.76	−0.0484
**100c**	11.48	8.05	3.43	−0.0489
**101a**	11.25	8.07	3.18	−0.0490

**58**	9.17	8.10	1.07	−0.0492
**121**	11.09	8.12	2.97	−0.0493
**44**	11.28	8.17	3.11	−0.0496
**123**	9.86	8.36	1.50	−0.0507
**101b**	10.87	8.40	2.47	−0.0509
**‹−E_{HB}(MTA, FBA)›, ‹π-contribution›**	8.75	6.69	2.06	

Table S67. Energy of the NBO hyperconjugative interactions (in kcal/mol), the ρ_{BCP} electron density at the hydrogen bond critical point (a.u.), the V_{BCP} potential energy density at the hydrogen bond critical point (a.u.), the $r_{O\cdots H}$ hydrogen bond length (Å), the n(LP-1) and n(LP-2) lone pairs orbital population (in *e*) and the n[σ*(O–H)] bond electronic population (in *e*) for sampling compounds from non-RAHB, RAHB, and arom-AHB clusters.

**No compounds**	Interaction energy		ρ_{BCP}	V_{BCP}	$r_{O\cdots H}$	n(LP-1)	n(LP-2)	Σ[n(LP-1) + n(LP-2)]	n[σ*(O–H)]
	LP-1(O)$_\sigma$ → σ*(O–H)	LP-2(O)$_\sigma$ → σ*(O–H)							
**compounds from non-RAHB cluster**									
**1-non-RAHB**	0	1.12	0.0152	–0.0121	2.2198	1.91758	1.98554	3.90312	0.00764
**8a-non-RAHB**	0.88	4.34	0.0249	–0.0197	2.0018	1.92056	1.97752	3.89808	0.01373
**11-non-RAHB**	0	1.89	0.0191	–0.0158	2.1029	1.92347	1.97947	3.90294	0.01035
**33a-non-RAHB**	2.88	12.31	0.0347	–0.0324	1.8001	1.88365	1.97549	3.85914	0.01039
**43-non-RAHB**	5.28	40.29	0.0651	–0.0712	1.5474	1.83861	1.97897	3.81758	0.06819
**62-non-RAHB**	2.75	5.98	0.0251	–0.0210	1.9012	1.91710	1.97432	3.89142	0.01885
**88b-non-RAHB**	4.79	12.51	0.0379	–0.0357	1.7350	1.91367	1.97407	3.88774	0.03248
**94-non-RAHB**	4.62	27.62	0.0499	–0.0489	1.6390	1.89937	1.97187	3.87124	0.05368
**compounds from RAHB cluster**									
**6-RAHB**	3.95	30.13	0.0516	–0.0523	1.6394	1.90943	1.97871	3.88814	0.01798
**17-RAHB**	5.07	50.39	0.0689	–0.0758	1.5249	1.88217	1.97289	3.85506	0.07597
**36-RAHB**	0.65	3.68	0.0173	–0.0137	2.1289	1.92003	1.98629	3.90632	0.01322
**42-RAHB**	2.33	11.69	0.0330	–0.0303	1.8270	1.85104	1.97437	3.82541	0.02793
**50-RAHB**	4.42	19.62	0.0411	–0.0391	1.7318	1.85595	1.97582	3.83177	0.03787
**78-RAHB**	9.26	93.91	0.0972	–0.1213	1.3953	1.83320	1.96771	3.80091	0.12071
**107-RAHB**	1.04	8.67	0.0252	–0.0213	1.9442	1.90698	1.97898	3.88596	0.02131
**compounds from arom-AHB cluster**									
**13-arom-AHB**	3.56	17.50	0.0411	–0.0400	1.7289	1.88426	1.97378	3.85804	0.03422
**14-arom-AHB**	2.80	11.41	0.0333	–0.0309	1.8195	1.86280	1.97834	3.84114	0.02418
**50-arom-AHB**	4.54	30.76	0.0536	–0.0555	1.6218	1.90166	1.97349	3.87515	0.05063
**62-arom-AHB**	0.92	6.76	0.0235	–0.0200	1.9741	1.90943	1.97871	3.88814	0.01798
**70-arom-AHB**	5.33	54.43	0.0733	–0.0821	1.5010	1.87665	1.97244	3.84909	0.08247
**104-arom-AHB**	0	1.20	0.0169	–0.0137	2.1172	1.91778	1.97968	3.89746	0.01150

Table S68. The R_1 and R_2 substituents and their Hammett constants for the studied compounds **1–54**.

No compounds	R_1 (R_2=NO_2)	No compounds	R_2 (R_1= $N(CH_3)_2$)	σ_p[a]
**1, 19, 37**	$N(CH_3)_2$	**10, 28, 46**	$N(CH_3)_2$	−0.83
**2, 20, 38**	NH_2	**11, 29, 47**	NH_2	−0.66
**3, 21, 39**	OH	**12, 30, 48**	OH	−0.37
**4, 22, 40**	CH_3	**13, 31, 49**	CH_3	−0.17
**5, 23, 41**	H	**14, 32, 50**	H	0
**6, 24, 42**	Cl	**15, 33, 51**	Cl	0.23
**7, 25, 43**	CHO	**16, 34, 52**	CHO	0.42
**8, 26, 44**	CN	**17, 35, 53**	CN	0.66
**9, 27, 45**	NO_2	**18, 36, 54**	NO_2	0.78

[a]Taken from Ref. 76

Table S69. Parameters of linear dependencies (S1)–(S30), $l = A\times[-E_\pi(PPE)] + B$, of bond lengths on the $-E_\pi$(PPE) values for subseries **Ia,b** – **IIIa,b** of studied compounds **1** – **54**.

N subseries	N compounds	R1 and R2	Bond length	N eqn	$A\times10^3$	B	r
**Ia**	**1 – 9**	R_1 varies, R_2=NO_2	$l(C_2–C_6)$	10	−2.94	1.4391	0.995
			$l(C_6=C_7)$	12	1.99	1.3483	0.999
			$l(C_7–C_8)$	11	−2.57	1.4562	0.997
			$l(C_2=O_9)$	13	0.73	1.1853	0.996
**Ib**	**10 – 18**	R_1=$N(CH_3)_2$; R_2 varies	$l(C_2–C_6)$	S1	−2.30	1.4308	0.988
			$l(C_6=C_7)$	S2	2.09	1.3479	0.982
			$l(C_7–C_8)$	S3	−5.41	1.4925	0.957
			$l(C_2=O_9)$	[a]	-	-	-
**IIa**	**19 – 27**	R_1 varies, R_2=NO_2	$l(C_2–C_6)$	S4	−4.77	1.4562	0.979
			$l(C_6=C_7)$	S5	2.21	1.4093	0.991
			$l(C_7–C_8)$	S6	−1.39	1.3813	0.989
			$l(C_8–C_9)$	S7	1.49	1.4056	0.988
			$l(C_9–C_{10})$	S8	−3.65	1.4650	0.960
			$l(C_{10}=O_{11})$	S9	0.69	1.1917	0.820
			$l(C_9=C_{12})$	S10	0.78	1.4925	0.957
			$l(C_{12}–C_{13})$	S11	−0.93	1.3856	0.992
			$l(C_6=C_{13})$	S12	1.96	1.4069	0.987
**IIb**	**28 – 36**	R_1=$N(CH_3)_2$; R_2 varies	$l(C_2–C_6)$	S13	−5.34	1.4561	0.995
			$l(C_6=C_7)$	S14	2.80	1.4088	0.937
			$l(C_7–C_8)$	S15	−2.86	1.3852	0.958
			$l(C_8–C_9)$	S16	3.36	1.4010	0.919
			$l(C_9–C_{10})$	S17	−13.55	1.5000	0.916
			$l(C_{10}=O_{11})$	[a]	-	-	-
			$l(C_9=C_{12})$	S18	1.96	1.3983	0.809
			$l(C_{12}–C_{13})$	S19	−2.42	1.3907	0.979
			$l(C_6=C_{13})$	S20	2.22	1.4064	0.984
**IIIa**	**37 – 45**	R_1 varies, R_2=NO_2	$l(C_2–C_6)$	S21	−3.05	1.4603	0.928
			$l(C_6–C_7)$	S22	1.34	1.4065	0.956
			$l(C_7=C_8)$	S23	−0.35	1.3912	0.817
			$l(C_8–C_9)$	S24	0.39	1.3892	0.907
			$l(C_9=C_{10})$	S25	0.12	1.4021	0.819
			$l(C_{10}–C_{11})$	S26	−1.43	1.4706	0.998
			$l(C_{11}=O_{12})$	[a]	-	-	-
			$l(C_{10}–C_{13})$	S27	0.21	1.3991	0.859
			$l(C_6=C_{13})$	S28	1.01	1.4008	0.935
**IIIb**	**46 – 54**	R_1=$N(CH_3)_2$; R_2 varies	$l(C_2–C_6)$	S29	−2.64	1.4583	0.886
			$l(C_6–C_7)$	[a]	-	-	-
			$l(C_7=C_8)$	[a]	-	-	-

			$l(C_8–C_9)$	[a]	-	-	-
			$l(C_9=C_{10})$	[a]	-	-	-
			$l(C_{10}–C_{11})$	S30	−22.40	1.5135	0.883
			$l(C_{11}=O_{12})$	[a]	-	-	-
			$l(C_{10}–C_{13})$	[a]	-	-	-
			$l(C_6=C_{13})$	[a]	-	-	-

[a]no correlation found

Table S70. Parameters of linear dependencies (S31) and (S32), $\nu_{C=O} = A\times[-E_\pi(\mathrm{PPE})] + B$, of the C=O frequency on the $-E_\pi$(PPE) values for subseries **Ia,b** – **IIIa,b** of studied compounds **1** – **54**.

N subseries	N compounds	R_1 and R_2	Frequency	N eqn	*A*	*B*	*r*
**Ia**	**1 – 9**	R_1 varies, R_2=NO_2	$\nu_{C=O}$	14	−2.53	1873.8	0.996
**Ib**	**10 – 18**	R_1=$N(CH_3)_2$; R_2 varies	$\nu_{C=O}$	[a]	-	-	-
**IIa**	**19 – 27**	R_1 varies, R_2=NO_2	$\nu_{C=O}$	S31	−5.20	1833.8	0.991
**IIb**	**28 – 36**	R_1=$N(CH_3)_2$; R_2 varies	$\nu_{C=O}$	[a]	-	-	-
**IIIa**	**37 – 45**	R_1 varies, R_2=NO_2	$\nu_{C=O}$	S32	−1.42	1845.5	0.861
**IIIb**	**46 – 54**	R_1=$N(CH_3)_2$; R_2 varies	$\nu_{C=O}$	[a]	-	-	-

[a]no correlation found

Table S71. Parameters of linear dependencies (S33)–(S44), *E*(HOMO), *E*(LUMO) and Δ*E* (HOMO-LUMO) = $A\times[-E_\pi(\mathrm{PPE})] + B$, of molecular orbital energy on the $-E_\pi$(PPE) values for subseries **Ia,b** – **IIIa,b** of studied compounds **1** – **54**.

N subseries	N compounds	R_1 and R_2	Molecular orbital energy	N eqn	$A\times10^3$	*B*	*r*
**Ia**	**1 – 9**	R_1 varies, R_2=NO_2	*E*(HOMO)	15	7.31	−0.299	0.996
			E(LUMO)	16	5.58	−0.164	0.976
			$\Delta E_{HOMO-LUMO}$	19	−1.73	0.135	0.866
**Ib**	**10 – 18**	R_1=$N(CH_3)_2$; R_2 varies	*E*(HOMO)	17	−3.31	−0.173	0.930
			E(LUMO)	18	−6.54	−0.028	0.958
			$\Delta E_{HOMO-LUMO}$	20	−3.24	0.145	0.847
**IIa**	**19 – 27**	R_1 varies, R_2=NO_2	*E*(HOMO)	S33	14.68	−0.254	0.988
			E(LUMO)	S34	9.15	−0.123	0.975
			$\Delta E_{HOMO-LUMO}$	S35	−5.53	0.131	0.927
**IIb**	**28 – 36**	R_1=$N(CH_3)_2$; R_2 varies	*E*(HOMO)	S36	−3.82	−0.186	0.955
			E(LUMO)	S37	−16.49	−0.0475	0.910
			$\Delta E_{HOMO-LUMO}$	S38	−12.66	0.138	0.841
**IIIa**	**37 – 45**	R_1 varies, R_2=NO_2	*E*(HOMO)	S39	17.15	−0.238	0.979
			E(LUMO)	S40	6.21	−0.117	0.981
			$\Delta E_{HOMO-LUMO}$	S41	−10.94	0.122	0.959
**IIIb**	**46 – 54**	R_1=$N(CH_3)_2$; R_2 varies	*E*(HOMO)	S42	−8.69	−0.179	0.904
			E(LUMO)	S43	−40.19	−0.032	0.807
			$\Delta E_{HOMO-LUMO}$	S44	−31.50	0.147	0.759

Atoms coordinates for newly calculated compounds 1 – 54.

1

0 1

6	2.677	-0.164	-0.074
6	2.861	-1.561	-0.025
6	1.591	-2.142	0.002
6	0.625	-1.130	-0.019
7	1.334	0.078	-0.060
6	-0.775	-1.231	0.006
6	-1.681	-0.200	-0.011
6	-3.078	-0.485	0.022
7	-3.976	0.878	-0.005
7	3.605	0.836	-0.155
1	3.807	-2.074	-0.011
1	1.369	-3.198	0.039
1	0.905	0.987	-0.076
1	-1.167	-2.244	0.044
1	-1.392	0.840	-0.050
8	-3.700	-1.503	0.065
6	3.192	2.212	0.083
6	5.000	0.494	0.095
1	4.027	2.874	-0.144
1	2.372	2.491	-0.583
1	2.883	2.389	1.123
1	5.629	1.323	-0.228
1	5.198	0.290	1.155
1	5.279	-0.384	-0.489
8	-3.391	1.955	-0.051
8	-5.176	0.724	0.022

2

0 1

6	3.345	0.535	-0.001
6	3.718	-0.814	0.027
6	2.535	-1.565	0.019
6	1.444	-0.690	-0.008
7	1.987	0.602	-0.015
6	0.067	-0.980	-0.007
6	-0.968	-0.084	-0.001
6	-2.315	-0.562	-0.006
7	-3.395	0.660	0.007
7	4.128	1.663	-0.079
1	4.731	-1.183	0.055
1	2.455	-2.641	0.046
1	1.451	1.448	-0.124
1	-0.183	-2.038	-0.012
1	-0.826	0.986	0.010
8	-2.786	-1.658	-0.016
1	3.770	2.506	0.347
1	5.110	1.520	0.103
8	-2.964	1.808	0.017
8	-4.561	0.337	0.006

3

0 1

6	-3.346	0.547	0.000
6	-3.732	-0.791	0.000
6	-2.542	-1.540	0.000

6	-1.455	-0.661	0.000
7	-1.997	0.628	0.000
6	-0.074	-0.957	-0.000
6	0.963	-0.068	-0.000
6	2.309	-0.560	-0.000
8	2.762	-1.663	-0.000
8	-4.047	1.694	0.001
1	-4.744	-1.161	0.000
1	-2.459	-2.616	0.000
1	-1.482	1.495	0.000
1	0.170	-2.015	-0.000
1	0.829	1.003	-0.000
7	3.402	0.644	-0.000
8	4.564	0.306	-0.000
8	2.984	1.796	-0.000
1	-4.992	1.504	0.001

4

0 1

6	-3.355	0.492	-0.000
6	-3.698	-0.858	-0.000
6	-2.507	-1.603	-0.000
6	-1.435	-0.706	0.000
7	-1.993	0.567	-0.000
6	-0.046	-0.984	0.000
6	0.976	-0.080	0.000
6	2.332	-0.550	0.000
7	3.405	0.674	0.000
6	-4.218	1.708	0.001
1	-4.707	-1.242	-0.000
1	-2.410	-2.678	-0.000
1	-1.467	1.426	-0.001
1	0.214	-2.038	0.000
1	0.826	0.989	-0.000
8	2.804	-1.643	0.000
8	2.964	1.818	-0.000
8	4.572	0.357	-0.000
1	-4.032	2.334	-0.878
1	-4.046	2.323	0.890
1	-5.270	1.421	-0.010

5

0 1

6	3.649	-1.097	0.000
6	4.159	0.194	0.000
6	3.060	1.071	0.000
6	1.890	0.305	0.000
7	2.291	-1.023	0.000
6	0.541	0.747	-0.000
6	-0.579	-0.028	-0.000
6	-1.872	0.601	-0.000
8	-2.207	1.743	-0.000
1	4.157	-2.048	0.000
1	5.205	0.456	0.000
1	3.088	2.150	0.000
1	1.671	-1.816	0.000
1	0.408	1.825	-0.000
1	-0.557	-1.108	-0.000
7	-3.083	-0.484	-0.000

8	-4.203	-0.029	-0.000
8	-2.781	-1.672	0.000

6

0 1

6	-3.018	-0.091	0.000
6	-3.276	1.270	-0.000
6	-2.023	1.907	0.000
6	-1.025	0.930	0.000
7	-1.675	-0.299	0.000
6	0.385	1.100	-0.000
6	1.332	0.124	-0.000
6	2.723	0.493	-0.000
7	3.704	-0.801	0.000
17	-4.117	-1.412	-0.000
1	-4.253	1.724	-0.000
1	-1.841	2.971	0.000
1	-1.236	-1.206	0.000
1	0.722	2.133	-0.000
1	1.103	-0.932	-0.000
8	3.267	1.552	0.000
8	3.180	-1.908	0.000
8	4.890	-0.567	0.000

7

0 1

6	3.042	-0.213	0.000
6	3.305	1.158	0.000
6	2.072	1.817	0.000
6	1.057	0.846	0.000
7	1.679	-0.379	0.000
6	-0.355	1.049	0.000
6	-1.316	0.091	-0.000
6	-2.706	0.485	-0.000
8	-3.224	1.554	-0.000
6	3.956	-1.353	0.000
1	4.291	1.593	0.000
1	1.902	2.883	0.000
1	1.210	-1.273	-0.000
1	-0.672	2.088	0.000
1	-1.106	-0.968	-0.000
7	-3.708	-0.788	-0.000
8	-4.889	-0.533	-0.000
8	-3.201	-1.902	-0.000
8	5.163	-1.256	0.000
1	3.463	-2.349	0.000

8

0 1

6	3.169	0.138	0.000
6	3.414	-1.234	-0.000
6	2.166	-1.872	-0.000
6	1.171	-0.888	0.000
7	1.811	0.331	0.000
6	-0.246	-1.069	0.000
6	-1.192	-0.097	0.000
6	-2.588	-0.475	0.000
7	-3.576	0.807	-0.000
6	4.080	1.218	-0.000

1	4.390	-1.690	-0.000
1	1.982	-2.935	-0.000
1	1.364	1.236	0.000
1	-0.578	-2.103	0.000
1	-0.969	0.960	0.000
8	-3.116	-1.539	0.000
8	-3.056	1.916	0.000
8	-4.759	0.564	-0.000
7	4.826	2.102	-0.000

9

0 1

6	2.777	0.116	-0.000
6	2.987	1.486	-0.000
6	1.711	2.066	0.000
6	0.756	1.037	0.000
7	1.444	-0.153	0.000
6	-0.668	1.161	0.000
6	-1.572	0.153	0.000
6	-2.985	0.476	0.000
7	-3.924	-0.839	-0.000
7	3.721	-0.962	-0.000
1	3.948	1.972	-0.000
1	1.481	3.120	0.001
1	1.074	-1.093	0.000
1	-1.041	2.182	0.000
1	-1.306	-0.894	0.000
8	-3.549	1.521	0.000
8	3.256	-2.104	-0.000
8	4.910	-0.673	-0.000
8	-5.115	-0.640	-0.000
8	-3.362	-1.926	-0.000

10

0 1

7	-3.908	0.636	-0.069
6	-5.331	0.392	0.132
6	-3.499	2.031	-0.146
1	-5.911	0.826	-0.689
1	-5.497	-0.681	0.163
1	-5.677	0.836	1.074
1	-4.348	2.621	-0.498
1	-3.188	2.436	0.826
1	-2.690	2.172	-0.864
6	2.807	-0.166	-0.092
6	2.959	-1.552	-0.015
6	1.663	-2.111	0.008
6	0.729	-1.084	-0.042
7	1.463	0.104	-0.096
6	-0.693	-1.156	-0.019
6	-1.585	-0.136	-0.044
6	-3.033	-0.434	0.008
7	3.759	0.832	-0.202
1	3.892	-2.087	0.021
1	1.418	-3.160	0.068
1	1.054	1.019	-0.168
1	-1.107	-2.159	0.026
1	-1.251	0.893	-0.077
8	-3.452	-1.587	0.116

6	3.397	2.175	0.232
6	5.135	0.432	0.054
1	4.224	2.850	0.010
1	2.531	2.544	-0.323
1	3.177	2.234	1.309
1	5.799	1.254	-0.219
1	5.313	0.168	1.107
1	5.393	-0.427	-0.568

11

0 1

7	-4.454	1.466	-0.040
6	2.045	-0.214	-0.094
6	2.022	-1.607	0.007
6	0.667	-1.999	0.047
6	-0.131	-0.864	-0.016
7	0.745	0.222	-0.096
6	-1.550	-0.757	0.013
6	-2.312	0.361	-0.032
6	-3.783	0.260	0.014
7	3.114	0.654	-0.226
1	2.882	-2.254	0.050
1	0.293	-3.008	0.129
1	0.454	1.179	-0.190
1	-2.085	-1.700	0.081
1	-1.873	1.353	-0.094
8	-4.394	-0.796	0.122
6	2.944	2.026	0.233
6	4.433	0.075	-0.012
1	3.841	2.593	-0.016
1	2.112	2.511	-0.285
1	2.773	2.098	1.318
1	5.190	0.805	-0.300
1	4.605	-0.218	1.035
1	4.557	-0.805	-0.644
1	-3.990	2.306	-0.342
1	-5.456	1.421	-0.148

12

0 1

8	-4.328	1.523	-0.033
6	2.038	-0.222	-0.090
6	2.020	-1.618	0.005
6	0.669	-2.013	0.042
6	-0.134	-0.879	-0.017
7	0.739	0.211	-0.090
6	-1.549	-0.772	0.011
6	-2.301	0.358	-0.026
6	-3.755	0.280	0.011
7	3.103	0.645	-0.211
1	2.881	-2.263	0.044
1	0.298	-3.024	0.116
1	0.444	1.168	-0.168
1	-2.083	-1.716	0.069
1	-1.871	1.351	-0.082
8	-4.436	-0.725	0.073
6	2.904	2.043	0.148
6	4.425	0.092	0.051
1	3.813	2.598	-0.084

1	2.102	2.487	-0.447
1	2.672	2.183	1.215
1	5.179	0.819	-0.252
1	4.579	-0.157	1.112
1	4.573	-0.811	-0.543
1	-5.285	1.381	-0.005

13

0 1

6	-4.530	1.538	-0.027
6	2.053	-0.212	-0.091
6	2.050	-1.608	0.006
6	0.703	-2.018	0.043
6	-0.113	-0.893	-0.017
7	0.750	0.206	-0.092
6	-1.528	-0.803	0.010
6	-2.305	0.312	-0.026
6	-3.771	0.220	0.012
7	3.109	0.667	-0.214
1	2.919	-2.243	0.047
1	0.343	-3.032	0.119
1	0.444	1.160	-0.176
1	-2.053	-1.753	0.068
1	-1.872	1.308	-0.081
8	-4.370	-0.846	0.074
6	2.898	2.059	0.155
6	4.437	0.126	0.046
1	3.800	2.625	-0.078
1	2.088	2.499	-0.433
1	2.670	2.190	1.224
1	5.184	0.861	-0.256
1	4.594	-0.125	1.105
1	4.593	-0.774	-0.551
1	-5.602	1.345	-0.001
1	-4.251	2.166	0.825
1	-4.282	2.096	-0.936

14

0 1

6	1.618	0.249	-0.087
6	1.457	1.638	0.003
6	0.075	1.893	0.040
6	-0.611	0.681	-0.015
7	0.371	-0.314	-0.087
6	-2.004	0.432	0.009
6	-2.650	-0.767	-0.039
6	-4.103	-0.853	-0.005
8	-4.874	0.091	0.069
7	2.765	-0.503	-0.203
1	2.248	2.367	0.038
1	-0.398	2.861	0.110
1	0.174	-1.298	-0.146
1	-2.631	1.318	0.073
1	-2.114	-1.710	-0.107
1	-4.506	-1.888	-0.054
6	4.025	0.178	0.065
6	2.706	-1.920	0.128
1	4.848	-0.471	-0.238
1	4.086	1.092	-0.526

1	4.150	0.437	1.127
1	3.668	-2.376	-0.106
1	2.482	-2.102	1.190
1	1.957	-2.430	-0.482

15

0 1

17	-4.317	1.347	-0.017
6	2.421	-0.175	-0.083
6	2.540	-1.573	-0.018
6	1.241	-2.098	0.015
6	0.325	-1.045	-0.017
7	1.088	0.128	-0.071
6	-1.085	-1.084	0.011
6	-1.949	-0.025	-0.014
6	-3.371	-0.259	0.023
7	3.394	0.787	-0.182
1	3.461	-2.130	0.003
1	0.971	-3.142	0.065
1	0.701	1.055	-0.104
1	-1.517	-2.079	0.057
1	-1.620	1.004	-0.060
8	-3.986	-1.279	0.073
6	3.047	2.173	0.100
6	4.767	0.378	0.085
1	3.905	2.804	-0.131
1	2.226	2.505	-0.541
1	2.767	2.338	1.151
1	5.440	1.176	-0.230
1	4.945	0.163	1.148
1	5.010	-0.512	-0.497

16

0 1

6	-4.358	1.036	-0.001
6	2.300	-0.194	-0.083
6	2.422	-1.593	-0.024
6	1.124	-2.120	0.009
6	0.206	-1.068	-0.019
7	0.967	0.106	-0.069
6	-1.204	-1.102	0.010
6	-2.064	-0.041	-0.010
6	-3.495	-0.232	0.025
7	3.272	0.769	-0.180
1	3.345	-2.148	-0.006
1	0.855	-3.164	0.056
1	0.576	1.031	-0.099
1	-1.646	-2.094	0.053
1	-1.723	0.988	-0.053
8	-4.095	-1.305	0.075
6	2.923	2.154	0.108
6	4.646	0.363	0.086
1	3.778	2.788	-0.126
1	2.098	2.485	-0.529
1	2.647	2.315	1.160
1	5.317	1.163	-0.227
1	4.824	0.146	1.149
1	4.890	-0.526	-0.497
8	-3.928	2.163	-0.046

1	-5.443	0.812	0.027

17

0 1

6	-4.323	1.081	-0.009
6	2.272	-0.165	-0.079
6	2.375	-1.568	-0.021
6	1.073	-2.076	0.010
6	0.167	-1.011	-0.018
7	0.944	0.153	-0.066
6	-1.240	-1.035	0.008
6	-2.100	0.030	-0.015
6	-3.530	-0.174	0.020
7	3.255	0.782	-0.168
1	3.291	-2.134	-0.003
1	0.790	-3.117	0.055
1	0.568	1.086	-0.091
1	-1.689	-2.024	0.052
1	-1.753	1.057	-0.059
8	-4.122	-1.243	0.070
6	2.924	2.177	0.088
6	4.627	0.360	0.089
1	3.793	2.793	-0.140
1	2.116	2.510	-0.571
1	2.631	2.361	1.131
1	5.305	1.151	-0.232
1	4.809	0.147	1.151
1	4.857	-0.533	-0.492
7	-4.944	2.054	-0.024

18

0 1

7	-3.976	0.878	-0.005
6	2.678	-0.164	-0.074
6	2.861	-1.561	-0.026
6	1.591	-2.142	0.002
6	0.625	-1.130	-0.019
7	1.334	0.078	-0.060
6	-0.775	-1.231	0.007
6	-1.681	-0.200	-0.011
6	-3.078	-0.485	0.022
7	3.605	0.836	-0.155
1	3.808	-2.074	-0.011
1	1.369	-3.198	0.039
1	0.905	0.987	-0.075
1	-1.167	-2.244	0.044
1	-1.392	0.840	-0.049
8	-3.700	-1.503	0.065
6	3.193	2.212	0.083
6	5.000	0.493	0.094
1	4.027	2.874	-0.145
1	2.372	2.492	-0.583
1	2.884	2.389	1.123
1	5.630	1.322	-0.229
1	5.199	0.290	1.155
1	5.279	-0.385	-0.489
8	-5.176	0.723	0.022
8	-3.391	1.955	-0.051

19

0 1

6	3.761	0.060	-0.081
6	3.770	1.443	0.114
6	2.427	1.868	0.150
6	1.600	0.762	-0.002
7	2.452	-0.342	-0.123
6	0.166	0.644	0.003
7	4.806	-0.823	-0.261
1	4.642	2.064	0.227
1	2.094	2.881	0.312
1	2.148	-1.269	-0.367
6	-0.652	1.801	-0.041
6	-0.486	-0.610	0.053
6	-1.865	-0.708	0.054
6	-2.662	0.448	0.009
6	-2.025	1.706	-0.037
1	0.090	-1.526	0.117
1	-2.321	-1.689	0.108
6	-4.113	0.419	-0.018
1	-0.189	2.778	-0.093
1	-2.637	2.599	-0.078
8	-4.900	1.317	-0.066
7	-4.725	-1.029	0.021
6	6.140	-0.310	0.022
6	4.581	-2.231	0.039
1	6.878	-1.033	-0.327
1	6.301	0.622	-0.523
1	6.306	-0.124	1.093
1	5.478	-2.792	-0.221
1	4.350	-2.411	1.100
1	3.769	-2.636	-0.570
8	-5.181	-1.463	-1.020
8	-4.731	-1.587	1.107

20

0 1

6	4.450	-0.538	-0.050
6	4.596	0.823	0.169
6	3.297	1.379	0.204
6	2.371	0.360	0.022
7	3.113	-0.816	-0.120
6	0.929	0.380	0.017
7	5.389	-1.543	-0.267
1	5.531	1.343	0.311
1	3.059	2.414	0.398
1	2.749	-1.685	-0.475
6	0.228	1.607	-0.076
6	0.164	-0.803	0.103
6	-1.219	-0.771	0.091
6	-1.900	0.453	-0.003
6	-1.149	1.643	-0.083
1	0.654	-1.764	0.210
1	-1.767	-1.701	0.174
6	-3.350	0.560	-0.046
1	0.783	2.533	-0.161
1	-1.673	2.587	-0.162
8	-4.045	1.527	-0.131
7	-4.096	-0.820	0.031
8	-4.578	-1.244	-1.002

8	-4.165	-1.339	1.134
1	5.272	-2.368	0.308
1	6.342	-1.212	-0.224

21

0 1

6	4.444	-0.554	-0.046
6	4.611	0.812	0.097
6	3.309	1.367	0.135
6	2.380	0.341	0.020
7	3.118	-0.840	-0.082
6	0.936	0.366	0.019
1	5.550	1.338	0.171
1	3.078	2.413	0.257
1	2.755	-1.765	-0.243
6	0.241	1.597	-0.040
6	0.165	-0.815	0.078
6	-1.218	-0.776	0.072
6	-1.893	0.453	0.011
6	-1.136	1.640	-0.043
1	0.647	-1.783	0.150
1	-1.769	-1.706	0.132
6	-3.343	0.568	-0.025
1	0.798	2.523	-0.097
1	-1.656	2.589	-0.095
8	-4.032	1.542	-0.080
7	-4.096	-0.809	0.012
8	-4.583	-1.198	-1.032
8	-4.165	-1.360	1.100
8	5.311	-1.585	-0.152
1	6.214	-1.252	-0.142

22

0 1

6	4.456	-0.508	-0.048
6	4.581	0.859	0.137
6	3.281	1.411	0.184
6	2.366	0.375	0.034
7	3.115	-0.786	-0.097
6	0.918	0.387	0.026
6	5.492	-1.573	-0.188
1	5.514	1.393	0.231
1	3.036	2.450	0.339
1	2.735	-1.699	-0.285
6	0.214	1.610	-0.066
6	0.158	-0.799	0.109
6	-1.225	-0.773	0.094
6	-1.909	0.449	0.000
6	-1.164	1.642	-0.077
1	0.649	-1.759	0.213
1	-1.770	-1.705	0.174
6	-3.362	0.551	-0.046
1	0.766	2.538	-0.145
1	-1.692	2.584	-0.155
8	-4.058	1.516	-0.130
7	-4.102	-0.832	0.024
8	-4.576	-1.255	-1.014
8	-4.175	-1.352	1.125
1	5.424	-2.082	-1.155

1	5.403	-2.333	0.596
1	6.488	-1.134	-0.114

23

0 1

6	4.781	-1.007	-0.169
6	5.029	0.317	0.133
6	3.778	0.966	0.234
6	2.781	0.024	-0.001
7	3.426	-1.178	-0.235
6	1.336	0.156	0.003
1	6.002	0.762	0.269
1	3.616	2.002	0.485
1	2.973	-2.033	-0.510
6	0.736	1.433	-0.072
6	0.485	-0.965	0.084
6	-0.892	-0.826	0.080
6	-1.473	0.448	0.004
6	-0.636	1.577	-0.069
1	0.901	-1.961	0.181
1	-1.511	-1.711	0.159
6	-2.915	0.668	-0.029
1	1.363	2.312	-0.152
1	-1.086	2.560	-0.135
8	-3.529	1.689	-0.096
7	-3.764	-0.650	0.034
8	-4.274	-1.025	-1.004
8	-3.873	-1.170	1.132
1	5.456	-1.829	-0.344

24

0 1

6	4.098	-0.164	-0.002
6	4.175	1.194	0.211
6	2.841	1.666	0.248
6	1.983	0.589	0.062
7	2.787	-0.534	-0.079
6	0.534	0.525	0.035
1	5.080	1.765	0.332
1	2.540	2.686	0.425
1	2.476	-1.463	-0.313
6	-0.226	1.705	-0.111
6	-0.160	-0.695	0.156
6	-1.544	-0.741	0.126
6	-2.287	0.439	-0.019
6	-1.605	1.665	-0.135
1	0.380	-1.623	0.305
1	-2.041	-1.697	0.238
6	-3.745	0.465	-0.081
1	0.279	2.656	-0.224
1	-2.180	2.575	-0.255
8	-4.484	1.392	-0.209
7	-4.416	-0.947	0.041
8	-4.866	-1.432	-0.979
8	-4.463	-1.426	1.162
17	5.352	-1.334	-0.175

25

0 1

6	4.143	-0.301	-0.034
6	4.216	1.060	0.251
6	2.904	1.550	0.313
6	2.030	0.483	0.071
7	2.806	-0.628	-0.127
6	0.574	0.453	0.040
1	5.133	1.608	0.400
1	2.608	2.562	0.540
1	2.462	-1.537	-0.397
6	-0.154	1.647	-0.129
6	-0.144	-0.749	0.181
6	-1.529	-0.765	0.146
6	-2.241	0.430	-0.025
6	-1.535	1.638	-0.160
1	0.378	-1.683	0.354
1	-2.049	-1.706	0.274
6	-3.703	0.487	-0.095
1	0.374	2.583	-0.257
1	-2.090	2.558	-0.298
8	-4.415	1.431	-0.243
7	-4.406	-0.905	0.046
8	-4.865	-1.394	-0.968
8	-4.463	-1.365	1.174
6	5.187	-1.294	-0.228
8	6.378	-1.067	-0.162
1	4.817	-2.320	-0.449

26

0 1

6	4.256	-0.215	-0.008
6	4.315	1.144	0.271
6	2.992	1.617	0.322
6	2.136	0.543	0.080
7	2.927	-0.565	-0.109
6	0.680	0.490	0.041
1	5.220	1.709	0.426
1	2.685	2.626	0.546
1	2.603	-1.479	-0.383
6	-0.063	1.669	-0.161
6	-0.020	-0.719	0.206
6	-1.405	-0.755	0.165
6	-2.133	0.425	-0.037
6	-1.444	1.639	-0.197
1	0.515	-1.641	0.403
1	-1.912	-1.700	0.312
6	-3.595	0.461	-0.115
1	0.453	2.609	-0.311
1	-2.011	2.547	-0.361
8	-4.318	1.391	-0.289
7	-4.281	-0.936	0.059
8	-4.736	-1.453	-0.942
8	-4.330	-1.371	1.198
6	5.291	-1.158	-0.185
7	6.136	-1.935	-0.332

27

0 1

6	3.844	0.019	0.052
6	3.878	1.382	0.285

6	2.538	1.805	0.300
6	1.720	0.690	0.080
7	2.547	-0.393	-0.062
6	0.267	0.589	0.023
7	4.902	-0.924	-0.088
1	4.767	1.972	0.430
1	2.191	2.809	0.484
1	2.298	-1.344	-0.298
6	-0.508	1.735	-0.238
6	-0.394	-0.635	0.227
6	-1.776	-0.718	0.167
6	-2.538	0.430	-0.092
6	-1.887	1.659	-0.290
1	0.169	-1.530	0.462
1	-2.254	-1.676	0.323
6	-4.004	0.424	-0.142
1	-0.020	2.685	-0.417
1	-2.481	2.542	-0.492
8	-4.752	1.330	-0.339
7	-4.648	-0.980	0.097
8	6.053	-0.515	0.025
8	4.578	-2.095	-0.316
8	-4.587	-1.763	-0.836
8	-5.182	-1.164	1.174

28

0 1

6	-3.873	0.025	0.102
6	-3.882	1.410	0.079
6	-2.528	1.840	0.045
6	-1.713	0.723	0.035
7	-2.559	-0.382	0.054
6	-0.265	0.594	-0.030
7	-4.902	-0.915	0.216
1	-4.757	2.039	0.067
1	-2.190	2.863	-0.022
1	-2.264	-1.318	0.280
6	0.570	1.676	0.314
6	0.357	-0.598	-0.445
6	1.742	-0.712	-0.484
6	2.561	0.367	-0.135
6	1.949	1.570	0.240
1	-0.244	-1.437	-0.777
1	2.187	-1.641	-0.824
6	4.056	0.337	-0.273
1	0.124	2.605	0.648
1	2.575	2.422	0.476
8	4.642	1.280	-0.794
7	4.731	-0.787	0.149
6	-6.201	-0.351	0.558
6	-4.985	-1.907	-0.861
1	-6.890	-1.165	0.793
1	-6.105	0.286	1.438
1	-6.638	0.243	-0.261
1	-5.653	-2.715	-0.554
1	-5.367	-1.471	-1.798
1	-4.005	-2.338	-1.062
6	6.162	-0.861	-0.120
6	4.252	-1.703	1.178
1	6.445	-1.907	-0.267

1	6.392	-0.288	-1.014
1	6.750	-0.454	0.713
1	4.893	-1.634	2.066
1	3.236	-1.456	1.473
1	4.276	-2.739	0.823

29

0 1

6	-3.183	-0.139	-0.075
6	-3.076	-1.492	0.207
6	-1.691	-1.800	0.269
6	-0.971	-0.642	0.038
7	-1.909	0.371	-0.154
6	0.460	-0.391	0.028
7	-4.292	0.673	-0.325
1	-3.894	-2.174	0.373
1	-1.268	-2.762	0.514
1	-1.696	1.266	-0.563
6	1.375	-1.453	-0.114
6	0.988	0.906	0.168
6	2.359	1.132	0.148
6	3.259	0.070	0.002
6	2.741	-1.228	-0.113
1	0.325	1.748	0.334
1	2.721	2.143	0.296
6	4.745	0.237	-0.014
1	1.002	-2.463	-0.240
1	3.437	-2.052	-0.212
8	5.507	-0.694	0.198
7	5.219	1.507	-0.256
6	-5.555	-0.042	-0.445
6	-4.402	1.886	0.491
1	-6.320	0.649	-0.804
1	-5.454	-0.850	-1.170
1	-5.898	-0.467	0.512
1	-5.159	2.542	0.056
1	-4.684	1.661	1.532
1	-3.456	2.428	0.506
1	6.218	1.585	-0.375
1	4.648	2.187	-0.731

30

0 1

6	-3.178	-0.144	-0.064
6	-3.069	-1.486	0.270
6	-1.686	-1.792	0.340
6	-0.966	-0.643	0.064
7	-1.904	0.364	-0.163
6	0.463	-0.394	0.040
7	-4.289	0.650	-0.347
1	-3.887	-2.161	0.463
1	-1.263	-2.745	0.618
1	-1.690	1.243	-0.603
6	1.376	-1.466	-0.055
6	0.993	0.910	0.109
6	2.361	1.135	0.077
6	3.254	0.061	-0.015
6	2.740	-1.243	-0.076
1	0.329	1.761	0.219

1	2.744	2.146	0.136
6	4.719	0.247	-0.052
1	1.001	-2.479	-0.131
1	3.434	-2.072	-0.152
8	5.538	-0.643	-0.132
8	5.096	1.555	0.012
6	-5.556	-0.067	-0.392
6	-4.378	1.930	0.359
1	-6.325	0.600	-0.788
1	-5.470	-0.926	-1.057
1	-5.882	-0.419	0.600
1	-5.149	2.543	-0.112
1	-4.630	1.801	1.423
1	-3.436	2.474	0.297
1	6.063	1.561	-0.018

31

0 1

6	-3.190	-0.134	-0.063
6	-3.089	-1.476	0.275
6	-1.707	-1.790	0.344
6	-0.980	-0.646	0.064
7	-1.913	0.366	-0.165
6	0.450	-0.406	0.039
7	-4.296	0.667	-0.347
1	-3.911	-2.145	0.470
1	-1.290	-2.744	0.624
1	-1.695	1.242	-0.610
6	1.358	-1.484	-0.053
6	0.990	0.893	0.102
6	2.362	1.104	0.070
6	3.256	0.029	-0.018
6	2.722	-1.270	-0.074
1	0.333	1.749	0.209
1	2.732	2.121	0.125
6	4.737	0.203	-0.054
1	0.975	-2.495	-0.125
1	3.411	-2.103	-0.148
8	5.480	-0.761	-0.132
6	5.312	1.608	0.010
6	-5.567	-0.043	-0.399
6	-4.382	1.942	0.368
1	-6.331	0.629	-0.795
1	-5.483	-0.901	-1.068
1	-5.898	-0.398	0.590
1	-5.146	2.564	-0.103
1	-4.640	1.807	1.430
1	-3.435	2.480	0.315
1	4.963	2.214	-0.831
1	6.399	1.541	-0.023
1	5.008	2.116	0.930

32

0 1

6	2.806	0.194	-0.064
6	2.622	1.534	0.250
6	1.225	1.763	0.314
6	0.569	0.571	0.054
7	1.563	-0.385	-0.156

6	-0.842	0.245	0.034
7	3.960	-0.536	-0.336
1	3.400	2.256	0.434
1	0.750	2.695	0.578
1	1.400	-1.282	-0.584
6	-1.813	1.271	-0.052
6	-1.300	-1.087	0.097
6	-2.657	-1.376	0.069
6	-3.608	-0.352	-0.014
6	-3.162	0.980	-0.070
1	-0.593	-1.902	0.199
1	-2.989	-2.409	0.122
6	-5.041	-0.680	-0.040
1	-1.488	2.302	-0.122
1	-3.900	1.771	-0.138
8	-5.946	0.127	-0.113
6	5.190	0.245	-0.361
6	4.108	-1.820	0.352
1	5.996	-0.377	-0.754
1	5.066	1.105	-1.021
1	5.486	0.607	0.637
1	4.919	-2.383	-0.115
1	4.335	-1.696	1.422
1	3.199	-2.415	0.265
1	-5.264	-1.768	0.014

33

0 1

6	-3.531	-0.090	-0.069
6	-3.496	-1.458	0.189
6	-2.136	-1.841	0.233
6	-1.349	-0.719	0.023
7	-2.234	0.351	-0.139
6	0.084	-0.552	0.013
7	-4.605	0.756	-0.296
1	-4.348	-2.099	0.344
1	-1.769	-2.832	0.449
1	-1.963	1.257	-0.481
6	0.939	-1.676	-0.063
6	0.692	0.721	0.075
6	2.068	0.868	0.058
6	2.904	-0.254	-0.016
6	2.311	-1.532	-0.072
1	0.082	1.613	0.168
1	2.503	1.857	0.112
6	4.370	-0.198	-0.038
1	0.511	-2.668	-0.131
1	2.951	-2.403	-0.134
8	5.126	-1.115	-0.099
17	5.090	1.503	0.038
6	-5.922	0.156	-0.125
6	-4.497	2.135	0.173
1	-6.678	0.855	-0.485
1	-5.992	-0.755	-0.720
1	-6.144	-0.091	0.925
1	-5.358	2.697	-0.190
1	-4.465	2.208	1.271
1	-3.607	2.620	-0.231

34

0 1

6	-3.440	-0.103	-0.076
6	-3.400	-1.480	0.134
6	-2.039	-1.858	0.173
6	-1.255	-0.726	0.008
7	-2.144	0.345	-0.123
6	0.177	-0.552	0.011
7	-4.516	0.745	-0.276
1	-4.250	-2.129	0.258
1	-1.669	-2.855	0.353
1	-1.873	1.269	-0.413
6	1.038	-1.673	-0.078
6	0.780	0.721	0.099
6	2.157	0.875	0.093
6	3.002	-0.245	0.005
6	2.407	-1.523	-0.076
1	0.167	1.609	0.202
1	2.582	1.866	0.159
6	4.470	-0.168	0.008
1	0.611	-2.665	-0.164
1	3.054	-2.388	-0.146
8	5.198	-1.153	-0.030
6	-5.831	0.145	-0.094
6	-4.396	2.123	0.189
1	-6.591	0.850	-0.433
1	-5.912	-0.757	-0.701
1	-6.038	-0.118	0.955
1	-5.275	2.681	-0.136
1	-4.317	2.197	1.284
1	-3.528	2.614	-0.255
6	5.237	1.168	0.050
8	4.791	2.276	-0.100
1	6.320	0.981	0.204

35

0 1

6	-3.383	-0.098	-0.072
6	-3.337	-1.480	0.127
6	-1.978	-1.852	0.160
6	-1.196	-0.715	0.001
7	-2.090	0.354	-0.120
6	0.233	-0.538	-0.000
7	-4.463	0.743	-0.252
1	-4.184	-2.134	0.246
1	-1.604	-2.850	0.325
1	-1.824	1.289	-0.374
6	1.096	-1.662	-0.058
6	0.834	0.740	0.054
6	2.209	0.889	0.046
6	3.051	-0.232	-0.011
6	2.465	-1.514	-0.060
1	0.221	1.631	0.129
1	2.637	1.885	0.093
6	4.510	-0.117	-0.020
1	0.670	-2.656	-0.115
1	3.114	-2.380	-0.108
8	5.295	-1.048	-0.067
6	-5.775	0.171	0.020
6	-4.300	2.154	0.072

1	-6.542	0.867	-0.323
1	-5.895	-0.760	-0.534
1	-5.936	-0.033	1.089
1	-5.215	2.683	-0.194
1	-4.091	2.327	1.138
1	-3.495	2.601	-0.519
6	5.061	1.260	0.034
7	5.530	2.314	0.074

36

0 1

6	3.762	0.060	-0.061
6	3.768	1.446	0.118
6	2.425	1.870	0.135
6	1.599	0.762	-0.014
7	2.453	-0.343	-0.113
6	0.166	0.643	-0.023
7	4.809	-0.824	-0.219
1	4.639	2.069	0.232
1	2.090	2.885	0.280
1	2.153	-1.273	-0.348
6	-0.652	1.799	-0.084
6	-0.486	-0.611	0.028
6	-1.865	-0.710	0.015
6	-2.662	0.446	-0.044
6	-2.025	1.704	-0.090
1	0.089	-1.526	0.100
1	-2.321	-1.691	0.050
6	-4.113	0.418	-0.032
1	-0.189	2.776	-0.138
1	-2.637	2.597	-0.137
8	-4.901	1.317	-0.069
7	-4.725	-1.028	0.043
6	6.140	-0.307	0.072
6	4.582	-2.229	0.094
1	6.882	-1.033	-0.261
1	6.306	0.619	-0.480
1	6.294	-0.109	1.143
1	5.482	-2.792	-0.150
1	4.339	-2.397	1.154
1	3.776	-2.641	-0.520
8	-5.195	-1.373	1.111
8	-4.716	-1.677	-0.991

37

0 1

6	3.123	0.057	-0.037
6	3.408	1.342	0.403
6	2.179	2.050	0.461
6	1.163	1.196	0.075
7	1.761	-0.031	-0.208
6	-0.275	1.393	0.005
7	3.955	-1.011	-0.359
1	4.380	1.721	0.672
1	2.049	3.062	0.812
1	1.319	-0.766	-0.737
6	-0.825	2.688	-0.086
6	-1.167	0.310	0.020
6	-2.549	0.514	-0.065

6	-3.075	1.811	-0.148
6	-2.199	2.890	-0.151
1	-0.779	-0.696	0.116
1	-0.159	3.542	-0.122
1	-2.589	3.899	-0.219
6	5.381	-0.723	-0.284
6	3.599	-2.319	0.196
1	5.933	-1.558	-0.720
1	5.607	0.175	-0.860
1	5.734	-0.577	0.750
1	4.184	-3.090	-0.310
1	3.795	-2.379	1.278
1	2.545	-2.541	0.028
6	-3.505	-0.599	-0.039
8	-4.694	-0.581	-0.093
7	-2.840	-2.020	0.073
8	-2.321	-2.455	-0.943
8	-2.928	-2.585	1.145
1	-4.147	1.951	-0.208

38

0 1

6	3.619	-0.914	-0.051
6	4.166	0.279	0.382
6	3.109	1.227	0.438
6	1.938	0.598	0.055
7	2.274	-0.725	-0.231
6	0.570	1.086	-0.010
7	4.198	-2.143	-0.381
1	5.197	0.440	0.654
1	3.190	2.245	0.786
1	1.695	-1.366	-0.750
6	0.298	2.465	-0.113
6	-0.525	0.210	0.023
6	-1.836	0.692	-0.056
6	-2.085	2.069	-0.149
6	-1.006	2.945	-0.171
1	-0.351	-0.853	0.128
1	1.125	3.163	-0.163
1	-1.180	4.012	-0.248
1	3.824	-2.932	0.134
1	5.206	-2.130	-0.311
6	-3.002	-0.199	-0.012
8	-4.162	0.066	-0.051
7	-2.644	-1.725	0.097
8	-2.241	-2.258	-0.925
8	-2.830	-2.259	1.172
1	-3.105	2.426	-0.204

39

0 1

6	3.614	-0.942	-0.053
6	4.186	0.245	0.355
6	3.127	1.193	0.421
6	1.948	0.567	0.060
7	2.275	-0.761	-0.216
6	0.584	1.066	-0.007
1	5.226	0.409	0.589
1	3.220	2.218	0.743

1	1.668	-1.457	-0.619
6	0.327	2.448	-0.107
6	-0.521	0.202	0.024
6	-1.826	0.700	-0.054
6	-2.059	2.080	-0.145
6	-0.970	2.944	-0.165
1	-0.362	-0.864	0.125
1	1.162	3.137	-0.156
1	-1.132	4.012	-0.241
6	-3.004	-0.176	-0.011
8	-4.160	0.106	-0.047
7	-2.669	-1.707	0.090
8	-2.258	-2.236	-0.931
8	-2.880	-2.248	1.157
1	-3.075	2.448	-0.198
8	4.115	-2.174	-0.311
1	5.071	-2.160	-0.207

40

0 1

6	3.638	-0.873	-0.049
6	4.157	0.331	0.387
6	3.092	1.264	0.462
6	1.929	0.616	0.077
7	2.287	-0.690	-0.218
6	0.555	1.093	-0.003
1	5.192	0.512	0.632
1	3.160	2.287	0.800
1	1.670	-1.379	-0.619
6	0.276	2.468	-0.120
6	-0.534	0.210	0.033
6	-1.848	0.683	-0.061
6	-2.103	2.058	-0.171
6	-1.031	2.941	-0.192
1	-0.356	-0.851	0.150
1	1.099	3.171	-0.170
1	-1.211	4.006	-0.281
6	-3.009	-0.214	-0.017
8	-4.170	0.046	-0.063
7	-2.645	-1.738	0.103
8	-2.213	-2.268	-0.909
8	-2.854	-2.273	1.173
1	-3.125	2.407	-0.235
6	4.300	-2.182	-0.327
1	5.368	-2.109	-0.118
1	3.892	-2.986	0.294
1	4.187	-2.482	-1.375

41

0 1

6	3.706	-1.700	-0.222
6	4.434	-0.647	0.288
6	3.546	0.447	0.438
6	2.288	0.038	0.020
7	2.409	-1.283	-0.369
6	1.017	0.755	-0.016
1	5.484	-0.666	0.535
1	3.788	1.417	0.844
1	1.682	-1.826	-0.806

6	0.988	2.160	-0.085
6	-0.209	0.077	0.015
6	-1.419	0.778	-0.038
6	-1.426	2.179	-0.101
6	-0.215	2.859	-0.117
1	-0.219	-1.002	0.097
1	1.923	2.705	-0.131
1	-0.205	3.942	-0.170
6	-2.722	0.101	0.003
8	-3.817	0.566	-0.014
7	-2.634	-1.466	0.072
8	-2.306	-2.032	-0.959
8	-2.933	-1.988	1.127
1	-2.371	2.707	-0.135
1	4.004	-2.700	-0.494

42

0 1

6	3.413	-0.311	0.030
6	3.801	0.925	0.489
6	2.624	1.718	0.542
6	1.555	0.946	0.118
7	2.062	-0.309	-0.182
6	0.135	1.263	0.002
1	4.802	1.213	0.764
1	2.564	2.736	0.894
1	1.558	-1.066	-0.615
6	-0.289	2.596	-0.153
6	-0.845	0.263	0.041
6	-2.201	0.585	-0.085
6	-2.605	1.920	-0.231
6	-1.639	2.917	-0.257
1	-0.551	-0.768	0.187
1	0.453	3.384	-0.208
1	-1.935	3.953	-0.375
6	-3.259	-0.434	-0.036
8	-4.439	-0.301	-0.116
7	-2.735	-1.903	0.140
8	-2.208	-2.407	-0.840
8	-2.927	-2.430	1.217
1	-3.658	2.151	-0.321
17	4.349	-1.726	-0.288

43

0 1

6	3.437	-0.419	-0.028
6	3.820	0.820	0.474
6	2.668	1.620	0.556
6	1.584	0.862	0.106
7	2.071	-0.372	-0.233
6	0.168	1.211	0.001
1	4.827	1.085	0.753
1	2.612	2.628	0.934
1	1.536	-1.107	-0.671
6	-0.226	2.553	-0.142
6	-0.830	0.230	0.040
6	-2.179	0.579	-0.080
6	-2.554	1.923	-0.218
6	-1.569	2.902	-0.243

1	-0.555	-0.807	0.179
1	0.532	3.326	-0.193
1	-1.846	3.944	-0.354
6	-3.260	-0.418	-0.033
8	-4.435	-0.256	-0.105
7	-2.766	-1.899	0.129
8	-2.232	-2.395	-0.852
8	-2.984	-2.439	1.194
1	-3.603	2.177	-0.303
6	4.214	-1.609	-0.329
8	5.414	-1.710	-0.178
1	3.620	-2.464	-0.724

44

0 1

6	3.543	-0.403	0.024
6	3.919	0.830	0.537
6	2.759	1.628	0.598
6	1.690	0.873	0.127
7	2.184	-0.363	-0.210
6	0.274	1.218	-0.004
1	4.918	1.101	0.838
1	2.697	2.634	0.982
1	1.666	-1.101	-0.662
6	-0.117	2.556	-0.181
6	-0.723	0.237	0.045
6	-2.071	0.584	-0.096
6	-2.444	1.925	-0.267
6	-1.460	2.904	-0.303
1	-0.449	-0.797	0.209
1	0.642	3.327	-0.242
1	-1.735	3.942	-0.440
6	-3.154	-0.411	-0.037
8	-4.328	-0.247	-0.122
7	-2.665	-1.889	0.156
8	-2.123	-2.403	-0.810
8	-2.894	-2.409	1.229
1	-3.492	2.176	-0.368
6	4.319	-1.548	-0.257
7	4.955	-2.487	-0.488

45

0 1

6	3.224	-0.047	0.049
6	3.537	1.221	0.502
6	2.323	1.931	0.537
6	1.302	1.079	0.107
7	1.878	-0.130	-0.181
6	-0.134	1.326	-0.024
7	4.058	-1.171	-0.206
1	4.520	1.563	0.777
1	2.189	2.947	0.875
1	1.444	-0.951	-0.578
6	-0.608	2.631	-0.239
6	-1.066	0.285	0.071
6	-2.434	0.546	-0.060
6	-2.891	1.854	-0.272
6	-1.970	2.891	-0.354
1	-0.727	-0.720	0.285

1	0.101	3.445	-0.332
1	-2.312	3.905	-0.522
8	5.259	-1.057	0.018
8	3.506	-2.188	-0.640
6	-3.450	-0.518	0.008
8	-4.633	-0.428	-0.075
7	-2.865	-1.953	0.214
8	-2.823	-2.672	-0.764
8	-2.527	-2.234	1.351
1	-3.953	2.035	-0.369

46

0 1

7	3.309	-1.611	0.226
6	4.226	-2.676	-0.165
6	2.669	-1.793	1.523
1	3.726	-3.641	-0.049
1	4.521	-2.534	-1.202
1	5.130	-2.674	0.456
1	3.425	-2.015	2.287
1	2.137	-0.893	1.820
1	1.963	-2.630	1.494
6	3.392	-0.411	-0.442
8	4.234	-0.209	-1.309
6	-3.277	-0.117	0.010
6	-3.646	1.197	-0.218
6	-2.454	1.975	-0.223
6	-1.380	1.133	-0.009
7	-1.905	-0.150	0.118
6	0.052	1.401	0.046
7	-4.026	-1.287	0.185
1	-4.648	1.556	-0.385
1	-2.391	3.034	-0.419
1	-1.402	-0.937	0.493
6	0.527	2.701	0.297
6	1.003	0.385	-0.152
6	2.373	0.653	-0.121
6	2.820	1.959	0.091
6	1.889	2.974	0.310
1	0.676	-0.623	-0.384
1	-0.182	3.499	0.486
1	2.230	3.986	0.495
6	-5.452	-1.067	0.383
6	-3.753	-2.362	-0.775
1	-5.919	-2.009	0.678
1	-5.605	-0.341	1.183
1	-5.960	-0.703	-0.525
1	-4.203	-3.289	-0.413
1	-4.160	-2.139	-1.775
1	-2.680	-2.523	-0.875
1	3.882	2.171	0.073

47

0 1

7	3.143	2.463	0.617
6	3.690	1.370	-0.016
8	4.787	1.420	-0.545
6	-2.851	-0.073	-0.052
6	-2.988	-1.348	0.468

6	-1.683	-1.908	0.563
6	-0.772	-0.972	0.116
7	-1.505	0.158	-0.239
6	0.684	-1.010	0.035
7	-3.793	0.886	-0.437
1	-3.912	-1.817	0.766
1	-1.437	-2.875	0.973
1	-1.152	0.891	-0.833
6	1.364	-2.236	-0.081
6	1.452	0.165	0.065
6	2.846	0.127	-0.036
6	3.498	-1.102	-0.169
6	2.750	-2.277	-0.175
1	0.951	1.122	0.162
1	0.794	-3.156	-0.126
1	3.251	-3.234	-0.272
6	-5.170	0.412	-0.447
6	-3.654	2.207	0.181
1	-5.802	1.168	-0.919
1	-5.239	-0.508	-1.030
1	-5.563	0.219	0.564
1	-4.279	2.922	-0.358
1	-3.955	2.205	1.241
1	-2.622	2.552	0.122
1	4.576	-1.117	-0.266
1	2.422	2.341	1.310
1	3.757	3.256	0.730

48

0 1

8	-4.893	-1.314	-0.017
6	-3.543	-1.450	0.022
8	-3.015	-2.538	0.081
6	2.865	0.071	-0.055
6	3.005	1.393	0.332
6	1.700	1.954	0.396
6	0.785	0.974	0.062
7	1.520	-0.180	-0.198
6	-0.670	0.997	0.005
7	3.806	-0.920	-0.349
1	3.932	1.893	0.562
1	1.459	2.958	0.709
1	1.153	-0.994	-0.664
6	-1.368	2.217	-0.089
6	-1.427	-0.182	0.043
6	-2.823	-0.151	-0.019
6	-3.498	1.071	-0.104
6	-2.756	2.250	-0.134
1	-0.954	-1.151	0.146
1	-0.810	3.144	-0.143
1	-3.265	3.205	-0.207
6	5.183	-0.449	-0.414
6	3.671	-2.177	0.394
1	5.811	-1.248	-0.813
1	5.247	0.408	-1.086
1	5.582	-0.157	0.571
1	4.295	-2.939	-0.077
1	3.977	-2.071	1.447
1	2.640	-2.528	0.375
1	-4.579	1.093	-0.148

1	-5.262	-2.209	0.012

49

0 1

6	2.879	0.061	-0.058
6	3.028	1.389	0.311
6	1.727	1.958	0.373
6	0.805	0.977	0.056
7	1.532	-0.182	-0.193
6	-0.650	1.006	0.005
7	3.814	-0.938	-0.341
1	3.958	1.887	0.531
1	1.493	2.967	0.672
1	1.156	-1.005	-0.636
6	-1.343	2.232	-0.080
6	-1.413	-0.169	0.037
6	-2.811	-0.142	-0.019
6	-3.474	1.089	-0.094
6	-2.730	2.269	-0.120
1	-0.944	-1.140	0.130
6	-3.542	-1.452	0.015
1	-0.783	3.158	-0.129
1	-3.238	3.224	-0.185
6	5.194	-0.477	-0.408
6	3.669	-2.188	0.410
1	5.818	-1.283	-0.798
1	5.265	0.374	-1.086
1	5.593	-0.178	0.576
1	4.291	-2.957	-0.053
1	3.971	-2.076	1.464
1	2.636	-2.535	0.389
1	-4.554	1.137	-0.135
8	-2.931	-2.505	0.054
6	-5.059	-1.446	0.000
1	-5.414	-2.475	0.035
1	-5.456	-0.895	0.857
1	-5.438	-0.962	-0.905

50

0 1

6	-2.523	-0.162	-0.058
6	-2.560	-1.501	0.296
6	-1.215	-1.957	0.356
6	-0.379	-0.899	0.052
7	-1.202	0.198	-0.187
6	1.073	-0.805	0.006
7	-3.540	0.755	-0.335
1	-3.444	-2.079	0.508
1	-0.898	-2.947	0.645
1	-0.898	1.052	-0.624
6	1.865	-1.972	-0.069
6	1.737	0.428	0.032
6	3.133	0.499	-0.021
6	3.897	-0.669	-0.087
6	3.252	-1.905	-0.106
1	1.194	1.362	0.118
6	3.813	1.813	0.002
1	1.379	-2.939	-0.112
1	3.830	-2.820	-0.163

6	-4.876	0.177	-0.407
6	-3.504	2.009	0.423
1	-5.566	0.931	-0.791
1	-4.873	-0.670	-1.094
1	-5.248	-0.164	0.573
1	-4.193	2.722	-0.034
1	-3.792	1.865	1.477
1	-2.506	2.446	0.401
1	4.980	-0.608	-0.124
8	3.253	2.887	0.049
1	4.922	1.758	-0.030

51

0 1

6	-3.222	-1.217	0.034
8	-2.755	-2.308	0.095
6	3.224	-0.006	-0.054
6	3.428	1.310	0.330
6	2.153	1.934	0.390
6	1.192	0.999	0.056
7	1.868	-0.192	-0.197
6	-0.259	1.096	0.003
7	4.118	-1.037	-0.347
1	4.377	1.764	0.563
1	1.962	2.949	0.702
1	1.465	-0.983	-0.673
6	-0.895	2.350	-0.096
6	-1.076	-0.041	0.046
6	-2.473	0.062	-0.013
6	-3.083	1.317	-0.102
6	-2.279	2.455	-0.138
1	-0.647	-1.030	0.153
1	-0.291	3.248	-0.154
1	-2.739	3.433	-0.213
6	5.519	-0.638	-0.379
6	3.902	-2.305	0.356
1	6.111	-1.461	-0.783
1	5.641	0.227	-1.032
1	5.914	-0.387	0.619
1	4.500	-3.084	-0.122
1	4.185	-2.244	1.418
1	2.856	-2.605	0.300
1	-4.159	1.402	-0.145
17	-5.048	-1.062	-0.011

52

0 1

6	3.161	0.025	-0.079
6	3.343	1.348	0.288
6	2.055	1.944	0.369
6	1.108	0.984	0.065
7	1.807	-0.192	-0.193
6	-0.346	1.046	0.038
7	4.071	-0.991	-0.379
1	4.286	1.827	0.495
1	1.847	2.959	0.672
1	1.410	-1.002	-0.641
6	-1.011	2.288	-0.038
6	-1.137	-0.107	0.088

6	-2.538	-0.040	0.051
6	-3.176	1.208	-0.016
6	-2.396	2.363	-0.054
1	-0.690	-1.089	0.184
6	-3.281	-1.319	0.131
1	-0.427	3.199	-0.098
1	-2.878	3.332	-0.113
6	5.461	-0.559	-0.453
6	3.904	-2.248	0.356
1	6.063	-1.376	-0.856
1	5.546	0.296	-1.124
1	5.874	-0.279	0.530
1	4.508	-3.023	-0.122
1	4.213	-2.158	1.409
1	2.864	-2.574	0.334
1	-4.253	1.274	-0.052
8	-2.738	-2.404	0.263
6	-4.819	-1.401	0.030
8	-5.548	-0.587	-0.474
1	-5.186	-2.371	0.420

53

0 1

6	3.102	0.013	-0.057
6	3.281	1.341	0.298
6	1.994	1.939	0.353
6	1.050	0.976	0.047
7	1.751	-0.205	-0.187
6	-0.401	1.040	0.003
7	4.018	-1.000	-0.336
1	4.221	1.821	0.514
1	1.784	2.956	0.645
1	1.360	-1.016	-0.637
6	-1.065	2.285	-0.081
6	-1.196	-0.112	0.042
6	-2.594	-0.031	-0.010
6	-3.230	1.214	-0.085
6	-2.449	2.368	-0.116
1	-0.756	-1.097	0.137
6	-3.364	-1.291	0.029
1	-0.477	3.193	-0.133
1	-2.926	3.339	-0.181
6	5.412	-0.577	-0.355
6	3.816	-2.276	0.355
1	6.023	-1.391	-0.751
1	5.526	0.288	-1.009
1	5.792	-0.316	0.646
1	4.439	-3.039	-0.117
1	4.078	-2.217	1.423
1	2.778	-2.600	0.278
1	-4.311	1.280	-0.122
8	-2.882	-2.403	0.086
6	-4.841	-1.169	-0.006
7	-5.993	-1.121	-0.032

54

0 1

6	3.123	0.057	-0.037
6	3.408	1.342	0.402

6	2.179	2.050	0.461
6	1.163	1.196	0.075
7	1.761	-0.031	-0.208
6	-0.275	1.393	0.005
7	3.955	-1.011	-0.359
1	4.380	1.721	0.672
1	2.049	3.062	0.812
1	1.319	-0.766	-0.737
6	-0.825	2.688	-0.086
6	-1.167	0.310	0.021
6	-2.549	0.514	-0.065
6	-3.075	1.811	-0.148
6	-2.199	2.890	-0.151
1	-0.779	-0.696	0.116
1	-0.159	3.542	-0.122
1	-2.589	3.899	-0.219
6	5.381	-0.723	-0.284
6	3.599	-2.319	0.196
1	5.933	-1.558	-0.720
1	5.607	0.175	-0.861
1	5.734	-0.577	0.749
1	4.184	-3.090	-0.309
1	3.795	-2.379	1.278
1	2.545	-2.541	0.029
1	-4.147	1.951	-0.208
6	-3.505	-0.599	-0.039
8	-4.694	-0.581	-0.093
7	-2.840	-2.019	0.073
8	-2.321	-2.455	-0.943
8	-2.928	-2.585	1.145
